# IFNAR1 stabilization sustains CD8^+^ T cell persistence in IFN-high colorectal cancer liver metastases

**DOI:** 10.1126/sciadv.aei0181

**Published:** 2026-11-04

**Authors:** Andrea Castiglioni, Andrea Monestiroli, Gloria Delfanti, Paola Zordan, Cristina Giordano, Leonardo Andreoni, Barbara Clissi, Charlotte B. Ekalle-Soppo, Lorena M. Ferreira, Francesca Caruso, Valentina Buttiglione, Iris Stefanetta, Federica Pedica, Claudio Doglioni, Federica Cipriani, Francesca Ratti, Ugo Elmore, Riccardo Rosati, Chiara Gnasso, Antonio Esposito, Vito Lampasona, Ilaria Marzinotto, Oronza A. Botrugno, Gabriele Metalli, Giovanni Tonon, Giulia M. Scotti, Lucrezia Pierfederici, Chiara Balestrieri, Chiara Bonini, Daniele V.F. Tauriello, Eduard Batlle, Andrea Brendolan, Giulia Casorati, Paolo Dellabona, Luca G. Guidotti, Giovanni Sitia

**Affiliations:** ^1^ Institute of Immunology and Infectious Diseases, IRCCS San Raffaele Scientific Institute, Milan, Italy.; ^2^ Pathology Unit, IRCCS San Raffaele Scientific Institute, Milan, Italy.; ^3^ Vita-Salute San Raffaele University, Milan, Italy.; ^4^ Hepatobiliary Surgery Division, IRCCS San Raffaele Hospital, Milan, Italy.; ^5^ Department of Gastrointestinal Surgery, IRCCS San Raffaele Hospital, Milan, Italy.; ^6^ Experimental Imaging Center, IRCCS San Raffaele Scientific Institute, Milan, Italy.; ^7^ Diabetes Research Institute, IRCCS San Raffaele Scientific Institute, Milan, Italy.; ^8^ Comprehensive Cancer Center, IRCCS San Raffaele Scientific Institute, Milan, Italy.; ^9^ Center for Omics Sciences, IRCCS San Raffaele Hospital, Milan, Italy.; ^10^ Institute for Research in Biomedicine (IRB Barcelona), The Barcelona Institute of Science and Technology (BIST), Barcelona, Spain.; ^11^ Departments of Medical Oncology and of Surgical Oncology and Gastrointestinal Surgery, Erasmus MC Cancer Institute, University Medical Center Rotterdam, Rotterdam, Netherlands.; ^12^ Centro de Investigación Biomédica en Red de Cáncer (CIBERONC), Barcelona, Spain.; ^13^ Institució Catalana de Recerca i Estudis Avançats (ICREA), Barcelona, Spain.

## Abstract

Colorectal cancer liver metastases (CRC-LMs) are associated with poor survival and frequently resist immunotherapy, yet the mechanisms underlying this immune refractoriness remain incompletely defined. Here, we identify a tumor-intrinsic type I interferon (IFN-I)–high state in CRC-LMs associated with loss of the IFN-α/β receptor subunit 1 (IFNAR1) on effector T lymphocytes within immune-enriched perimetastatic niches. Reduced IFNAR1 expression stratifies patients with poor clinical outcomes. Transcriptional profiling of patient-derived organoids and complementary preclinical models revealed heterogeneous but recurrent tumor-derived IFN-I production in CRC-LMs. In IFN-I^high^ contexts, sustained signaling promoted IFNAR1 internalization on effector CD8^+^ T cells, impairing their persistence, survival, and effector function. Pharmacologic or genetic stabilization of IFNAR1 on cell surface restored CD8^+^ T cell fitness and selectively enhanced T cell receptor– and chimeric antigen receptor–based immunotherapies in IFN-I^high^ tumors. These findings define a context-dependent IFN-I^high^ / IFNAR1^low^ axis that constrains antitumor immunity in CRC-LMs and identify IFNAR1 stabilization as a strategy to convert chronic IFN signaling from a suppressive barrier into a therapeutic vulnerability.

## INTRODUCTION

Colorectal cancer (CRC) is among the most prevalent malignancies worldwide and remains a leading cause of cancer-related mortality (*1*). Metastatic spread to the liver occurs in up to 50 to 60% of patients during disease progression, with ∼20% presenting with synchronous liver metastases (LMs) at diagnosis. Despite advances in systemic therapies, including combination chemotherapy and targeted agents (*2*), long-term survival for patients with CRC-LMs remains limited, particularly in the setting of unresectable disease (*3*).

Adoptive cell therapies (ACTs), including T cell receptor (TCR)–engineered and chimeric antigen receptor T cells (CAR T cells), have achieved durable responses in several hematologic and selected solid malignancies (*4*, *5*), but clinical activity in CRC-LM has been limited (*6*). This limited efficacy likely reflects, at least in part, unique features of the liver metastatic microenvironment (mTME), which can impose immunoregulatory constraints on T cell infiltration, persistence, and function (*7*–*9*). A clearer understanding of metastasis-specific immune regulation in CRC-LM is therefore required to inform strategies that enhance ACT efficacy in this setting. Among candidate pathways, inflammatory signaling networks that shape antigen-driven T cell responses, such as type I interferon (IFN-I) sensing, may represent critical determinants of therapeutic responsiveness.

IFN-I are cytokines best known for antiviral defense (*10*) but also exert pleiotropic effects in cancer immunity that are highly context and time dependent (*11*–*15*). Acute IFN-I signaling promotes antitumor surveillance by enhancing cross-priming of tumor-specific CD8^+^ T cells and up-regulating major histocompatibility complex class I (MHC-I) expression on tumor cells (*16*–*19*). Consistent with this protective role, perioperative or targeted IFN-α administration can limit CRC seeding of the liver through effects on the hepatic vascular niche (*20*, *21*). However, sustained IFN-I exposure can instead drive adaptive immune resistance, including induction of inhibitory pathways (e.g., PD-1/PD-L1 and other IFN-stimulated suppressive programs) (*22*–*24*), expansion of regulatory T cells (*25*, *26*), and progressive CD8^+^ T cell dysfunction culminating in terminal exhaustion (*24*, *27*–*30*).

Cellular responsiveness to IFN-I is critically governed by the stability of IFN-α/β receptor subunit 1 (IFNAR1), whose surface expression can be reduced by tumor-associated stress cues that promote receptor internalization and proteasomal degradation (*31*–*33*). Loss of IFNAR1 has been associated with immune evasion in primary CRC (*34*–*36*), suggesting that receptor destabilization may represent a tumor-exploited mechanism to blunt effective immune responses. Whether chronic IFN-I signaling in CRC-LM similarly enforces IFNAR1 destabilization on tumor-infiltrating T cells, and whether preventing this preserves antitumor function, remains unknown.

Here, we sought to determine whether chronic IFN-I signaling in CRC-LMs enforces IFNAR1 destabilization on tumor-infiltrating T cells and thereby constrains effective antitumor immunity. We identify a previously unrecognized state of the mTME in CRC-LMs, characterized by chronic IFN-I signaling and loss of IFNAR1 on infiltrating lymphocytes, and associated with adverse clinical outcome. We demonstrate that CRC-LMs frequently sustain elevated IFN-I levels. Prolonged tumor-derived IFN-I exposure drives IFNAR1 down-regulation on effector CD8^+^ T cells, resulting in functional impairment, exhaustion, apoptotic loss, and exclusion from metastatic foci. Notably, stabilizing IFNAR1 preserves CD8^+^ T cell persistence and function and enhances the efficacy of TCR- and CAR-based ACTs in preclinical models. Together, these findings define a tumor-driven mechanism of immune evasion in CRC-LM and establish IFNAR1 stabilization as a strategy to convert chronic IFN-I signaling from an immunosuppressive liability into a therapeutic vulnerability.

## RESULTS

### Low IFNAR1 expression at the invasive margin in perimetastatic liver T lymphocytes associates with poor prognosis

To assess whether IFNAR1 expression in CRC-LM is spatially regulated and clinically relevant, we performed immunohistochemical staining for IFNAR1 in a retrospective cohort of 44 patients who underwent combined resection of primary CRC and synchronous LMs (human synchronous cohort) (table S1 and fig. S1A). In normal colon mucosa, IFNAR1 was detected in immune cells within the lamina propria, whereas epithelial and tumor cells showed low and predominantly nonspecific/background staining (fig. S1, B and C), consistent with prior reports (*34*). In paired liver specimens, IFNAR1^+^ cells were rare in uninvolved parenchyma and intrametastatic regions but were spatially enriched at the perimetastatic invasive margin, with variable extent of staining across cases (Fig. 1, A and B).

**Fig. 1.**
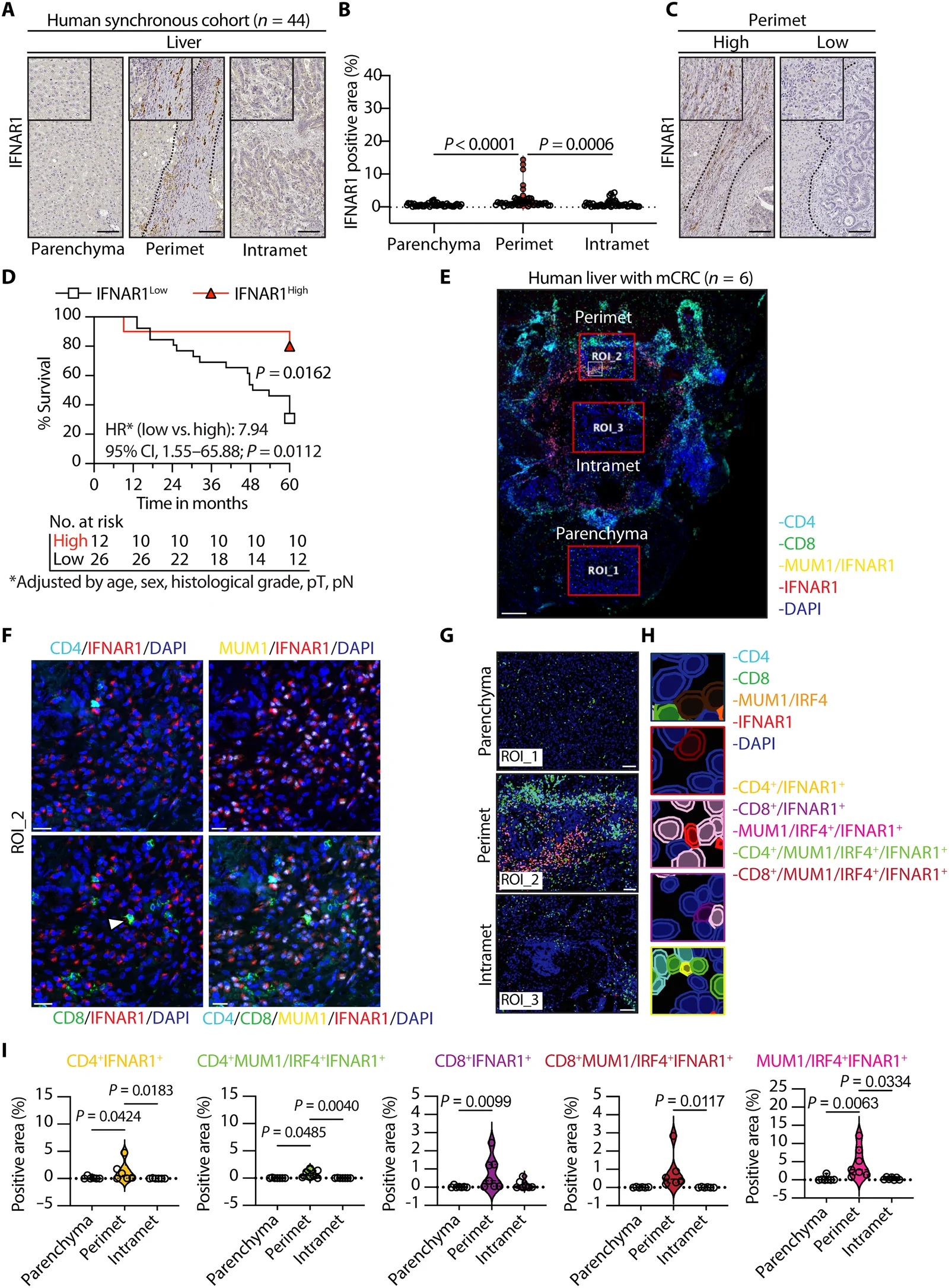
Perimetastatic IFNAR1 loss in CRC-LMs associates with poor outcome. IFNAR1 was assessed by immunohistochemistry (IHC) in paired CRCs and LMs from 44 patients. (**A**) IFNAR1 immunohistochemical images of uninvolved liver parenchyma, the perimetastatic invasive front (perimet), and the intrametastatic region (intramet). Dashed lines indicate the invasive front. Scale bar, 100 μm; insets show magnified regions. (**B**) IFNAR1-positive area in the regions shown in (A). Each dot represents one patient (*n* = 42 per region; *n* = 41 complete matched cases). (**C**) Representative perimetastatic IFNAR1^high^ and IFNAR1^low^ cases, classified using the mean perimetastatic IFNAR1-positive area as the cutoff (2.31%). Scale bar, 100 μm; insets show magnified regions. (**D**) Kaplan-Meier analysis of patients with survival follow-up (IFNAR1^high^, *n* = 11; IFNAR1^low^, *n* = 26). Complete-case multivariable Cox analysis adjusted for age, sex, histologic grade, pT, and pN showed increased mortality associated with IFNAR1^low^ status [adjusted hazard ratio (HR), 7.94; 95% profile-likelihood confidence interval (CI), 1.55 to 65.88; likelihood-ratio *P* = 0.0112; *n* = 28 patients, 17 events]. (**E**) Multiplex immunofluorescence of LMs stained for CD4, CD8, IFNAR1, MUM1/IRF4, and 4′,6-diamidino-2-phenylindole (DAPI) (*n* = 6 patients, 7 sections). Boxes indicate parenchyma, perimet, and intramet regions analyzed in (G); the white box indicates the area shown in (F). Scale bar, 2 mm. (**F**) High-magnification images showing IFNAR1 colocalization with CD4, CD8, and MUM1/IRF4. The arrowhead indicates a CD8^+^IFNAR1^+^ cell. Scale bar, 20 μm. (**G** and **H**) Representative regions and QuPath/StarDist-based phenotyping of IFNAR1-positive immune-cell populations. (**I**) Frequencies of the indicated populations across regions. Each dot represents one section; one patient contributed two sections. Statistical significance was assessed using Friedman’s test with Dunn’s multiple-comparisons correction [(B) and (I)], the two-sided log-rank test (D), and multivariable Cox proportional-hazards analysis (D). Exact *P* values are shown.

To examine the clinical relevance of this spatial pattern, we stratified patients with available IFNAR1 measurements into IFNAR1^high^ (*n* = 12) and IFNAR1^low^ (*n* = 30) groups on the basis of the cohort mean percentage of IFNAR1^+^ area detected at the invasive margin (Fig. 1, B and C, and fig. S1A). IFNAR1^high^ patients exhibited improved estimated 5-year overall survival compared with IFNAR1^low^ patients (81.8% versus 30.8%; log-rank *P* = 0.0162; Fig. 1D). In an exploratory complete-case multivariable Cox proportional hazards model adjusted for age, sex, histologic grade, pT, and pN, IFNAR1^low^ status was associated with an increased risk of death compared with IFNAR1^high^ status (adjusted hazard ratio, 7.94; 95% profile-likelihood confidence interval, 1.55 to 65.88; likelihood-ratio *P* = 0.0112).

IFNAR1^high^ patients were more frequently treatment naïve than IFNAR1^low^ patients (9 of 12, 75% versus 9 of 30, 30%; two-sided Fisher’s exact test, *P* = 0.0144; fig. S1D). Because IFNAR1 expression was assessed retrospectively only in surgical specimens, the observed treatment imbalance does not permit causal inference regarding the effects of neoadjuvant chemotherapy on IFNAR1 expression or clinical outcome.

Because the tumor invasive margin represents a site of heightened immune activity in CRC-LMs (*37*) and lymphocyte density correlates with improved clinical outcome (*38*), we performed multiplex immunofluorescence analysis (Akoya Phenoptics platform) on six top representative IFNAR1^High^ cases, with optimal tissue quality, to characterize the cellular distribution of IFNAR1 within the metastatic niche (Fig. 1E and fig. S1E). IFNAR1 expression localized to distinct lymphocyte lineage and activation states. Using CD4 and CD8 to delineate T cell subsets and MUM1/IRF4 as a marker of late lymphocyte activation (*39*, *40*), we identified four IFNAR1^+^ T cell populations: CD4^+^IFNAR1^+^, CD4^+^MUM1/IRF4^+^IFNAR1^+^, CD8^+^IFNAR1^+^, and CD8^+^MUM1/IRF4^+^IFNAR1^+^ cells (Fig. 1, F to H). In addition, a fifth IFNAR1^+^ population composed of MUM1/IRF4^+^IFNAR1^+^ lymphoid cells was detected (Fig. 1, G to I), consistent with the known expression of MUM1/IRF4 in activated B-lineage cells (*39*). All five IFNAR1^+^ subsets were spatially enriched within immune-dense perimetastatic regions at the invasive front compared with adjacent noninvolved liver parenchyma and intrametastatic areas (Fig. 1I). These findings indicate that IFNAR1 expression in CRC-LM is spatially restricted to defined lymphocyte populations within perimetastatic immune niches.

### A subset of human CRC-LMs produces high levels of IFN-Is

The association between reduced IFNAR1 expression at the invasive margin and poor prognosis prompted us to investigate potential upstream drivers of IFNAR1 destabilization. As surface IFNAR1 levels are regulated by multiple stress-related signals, including sustained exposure to IFN-Is themselves (*31*–*33*, *41*), we hypothesized that CRC-LMs may produce endogenous IFN-I subtypes within the TME. As an initial, hypothesis-generating step, we examined whether activation of IFN-I–related transcriptional programs associates with clinical outcome in CRC. We analyzed primary tumor RNA-seq data and clinical outcomes from the TCGA Colon Adenocarcinoma cohort (TCGA-COAD). Of 453 available cases, 442 were included after excluding patients with missing covariates required for survival modeling. In multivariable Cox models adjusted for age, year of diagnosis, and stage, high IRF7 and IFN ε (IFNE) expression were associated with increased risk of death, whereas other IFN-I subtypes and ISG regulators did not correlate with outcome (Fig. 2A). These findings indicate that elevated expression of selected IFN-I pathway components associates with poorer clinical outcome in CRC, supporting the possibility that sustained IFN-I signaling, classically associated with antitumor immunity, may instead correlate with adverse disease biology in CRC.

**Fig. 2.**
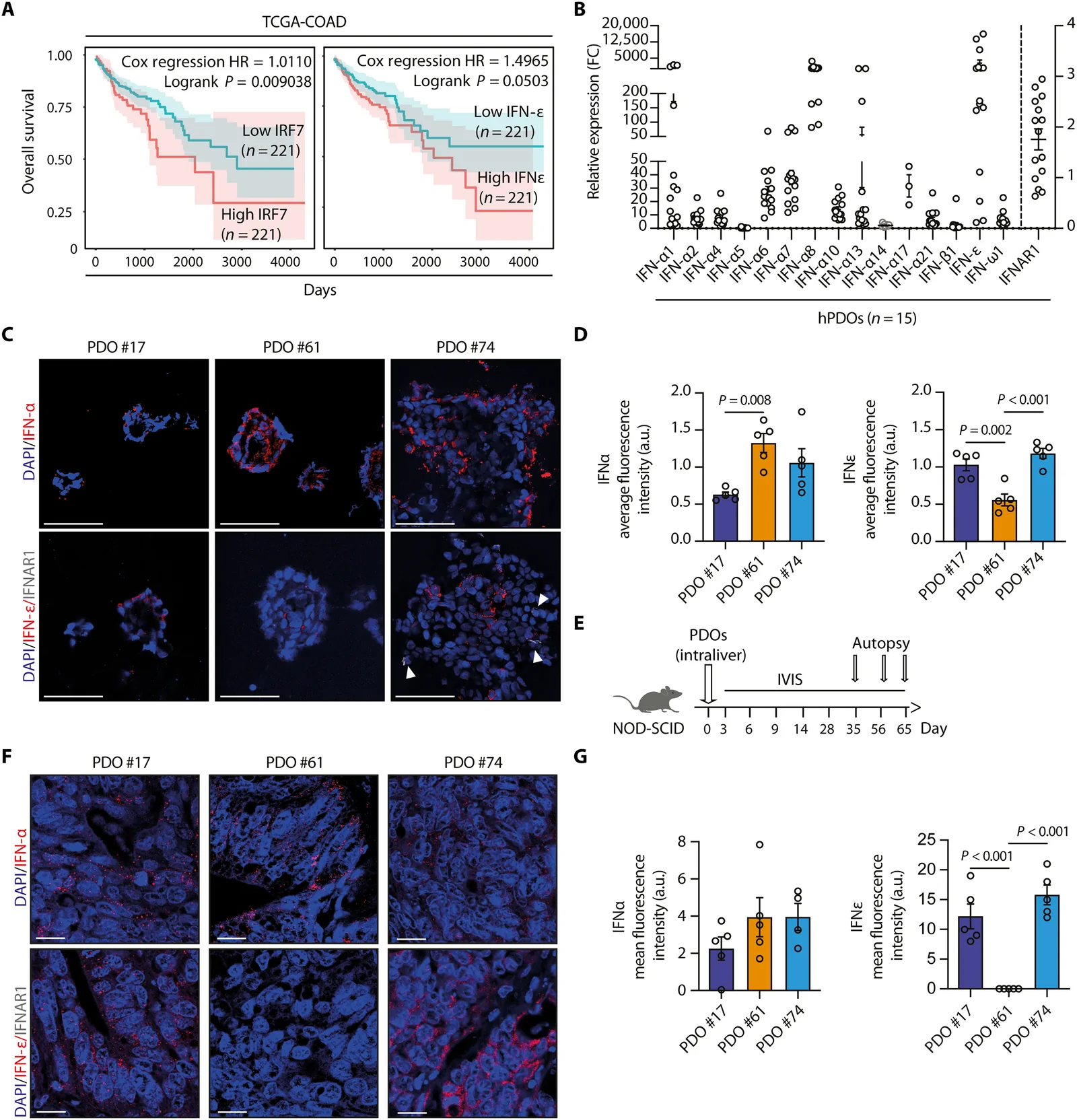
IFN-I expression by PDOs derived from CRC-LMs. (**A**) Kaplan-Meier analysis of overall survival in the TCGA-COAD cohort stratified by IRF7 or IFNε mRNA expression (*n* = 221 per group). Hazard ratios were estimated by multivariable Cox regression adjusted for age, year of diagnosis, and tumor stage. (**B**) Relative mRNA expression of IFN-Is and IFNAR1 in PDOs from CRC-LMs (*n* = 15 independent lines), normalized to glyceraldehyde-3-phosphate dehydrogenase and expressed relative to normal colonic crypts. Each dot represents one PDO line; bars indicate means ± SEM. (**C**) Representative immunofluorescence images of pan–IFN-α or IFNε, with IFNAR1 and DAPI, in PDO #17, #61, and #74. Pan–IFN-α staining does not distinguish individual IFN-α subtypes. Arrowheads indicate IFNAR1-positive cells. Scale bars, 50 μm. (**D**) Mean fluorescence intensity (MFI; arbitrary units) of pan–IFN-α and IFNε in the PDOs shown in (C). Each dot represents one field; five fields per PDO were analyzed. Bars indicate means ± SEM. (**E**) Orthotopic intrahepatic implantation of luciferase-expressing PDOs into NOD-SCID mice, with IVIS monitoring and tissue collection at the indicated time points (*n* = 3 mice per PDO line). (**F**) Representative immunofluorescence images of liver lesions generated by PDO #17, #61, and #74, stained for pan–IFN-α or IFNε, with IFNAR1 and DAPI. Scale bars, 50 μm. (**G**) MFI of pan–IFN-α and IFNε in mouse liver lesions generated by the indicated PDO lines. Four to five fields from one to two lesions were analyzed per line. Each dot represents one field; bars indicate means ± SEM. Statistical significance was assessed using two-sided log-rank tests and multivariable Cox regression (A) or ordinary one-way analysis of variance (ANOVA) with Tukey’s multiple-comparisons test [(D) and (G)]. Exact or adjusted *P* values are shown.

As TCGA transcriptomic data derive from primary tumors rather than metastatic lesions, we next examined IFN-I expression directly in CRC-LMs using 15 patient-derived organoids (hPDOs) from the ACC_ORG-LiMeT cohort (table S2 and fig. S2A). Across the cohort, hPDOs displayed a broad dynamic range of IFN-I transcript expression. *IFNα8* was consistently up-regulated, whereas *IFNα1*, *IFNα13*, and *IFN*ε were elevated in a subset of samples (Fig. 2B). By contrast, *IFNα5* and *IFNα14* were expressed at uniformly low levels. *IFNAR1* mRNA was detected at low levels across all organoids (Fig. 2B), with only three patients displaying IFNAR1-high levels exclusively at the perimetastatic invasive front (fig. S2A). These data indicate that CRC-LM–derived organoids display heterogeneous but frequently elevated IFN-I transcriptional programs.

To determine whether transcriptional activation corresponded to protein production, we performed immunofluorescence analysis on three representative PDOs (#17, #61, and #74). Consistent with transcript data, PDOs #61 and #74 showed higher pan–IFN-α and IFNε staining compared with PDO #17, which displayed weak and sparse signal (Fig. 2C). Spatially, IFN-I staining patterns differed between organoids: in PDO #61, signal was broadly distributed, whereas in PDO #74 it was more regionally restricted (Fig. 2, C and D). Across all three models, IFNAR1 protein was largely undetectable, with only rare IFNAR1^+^ cells observed in PDO #74 (Fig. 2C). Together, these findings support the presence of IFN-I–producing CRC-LM organoids in which IFNAR1 protein expression is limited.

To determine whether PDOs retain IFN-I production in vivo, we orthotopically implanted them into immunodeficient nonobese diabetic mice–severe combined immunodeficient (NOD-SCID) mice via intrahepatic injection (Fig. 2E). All PDOs successfully engrafted, exhibited distinct growth kinetics (fig. S2, B and C), and generated macroscopically visible hepatic lesions (fig. S2D). Histopathological analysis confirmed that murine lesions recapitulated key morphological features of the corresponding patient-derived metastases (fig. S2E). Model-specific histopathologic characteristics were observed, including differential recruitment of F4/80^+^ myeloid cells, while human carcinoembryonic antigen (hCEA) expression was maintained across models (fig. S2, F and G). Immunofluorescence analysis of whole mouse liver sections confirmed robust human IFN-α and IFNε protein expression within tumor regions, although IFNε was undetectable in PDO #61–derived lesions (Fig. 2, F and G). IFNAR1 protein remained largely at background levels across models (Fig. 2F). Last, we examined established human CRC cell lines derived from metastatic lesions (COLO205, MSS; SW620, MSS) (fig. S2H). Both cell lines exhibited low IFNAR1 protein levels, consistent with patient findings, and elevated IFN-α and IFNε expression (fig. S2, H and I). Collectively, these data demonstrate that a subset of CRC models, including LM-derived organoids, exhibits elevated IFN-I production sustaining the idea that this pathway contributes to the establishment of receptor-low state in mTME of CRC-LMs.

### IFNAR1^low^ MC38 CRC cells generate an IFN-enriched mTME

To mechanistically interrogate tumor-intrinsic IFN-I production in CRC-LMs, we used the immunogenic, microsatellite-unstable (MSI-like), murine MC38 CRC cell line (H2^b^-restricted) (*42*, *43*), which was injected via the superior mesenteric vein into C57BL/6 mice to establish experimental CRC-LMs (fig. S3A). Mice were euthanized at 7 and 21 days after tumor injection to assess temporal changes in the transcriptional levels of IFN-I subtypes and *Ifnar1* within metastatic livers. Quantitative analysis revealed that increasing tumor burden was associated with notable up-regulation of *Ifnα1/6/5*, *Ifnα13*, *Ifnβ*, and *Ifnε*, whereas *Ifnα11/12* and *Ifnαβ* showed upward trends that did not reach statistical significance (fig. S3B). By contrast, *Ifnar1* transcript levels remained comparable to sham livers, indicating that enhanced ligand expression was not accompanied by increased receptor transcription in this model.

To determine whether IFN-I production differed across CRC genotypes, we compared MC38 with the genetically defined MSS-like CRC organoid line MTO140 (H^2b^-restricted; *Apc*^−/−^, *Kras*^LSL-G12D^, *Tgfbr2*^−/−^, and *Trp53*^−/−^) (*44*). Guided by the in vivo screen, we focused on the most differentially expressed IFN-I subtypes and quantified tumor cell–intrinsic expression by digital polymerase chain reaction (PCR) (Fig. 3A). Relative to normal intestinal crypt (IC) epithelium (fig. S3C), MC38 cells expressed markedly higher levels of *Ifn*α*1/6/5*, *Ifnα13*, and *Ifnβ* compared with MTO140, whereas *Ifnε* displayed an inverse pattern (Fig. 3A). These transcriptional differences were corroborated at the protein and functional levels. Immunofluorescence analysis demonstrated higher intracellular pan–IFN-α signal in MC38 compared with MTO140 cells (Fig. 3, B and C). Consistently, MC38 released increasing amounts of IFN-Is into culture supernatant over time, with measurable bioactivity, whereas MTO140 secreted only low IFN-α concentrations at later time points and exhibited minimal functional activity (Fig. 3D).

**Fig. 3.**
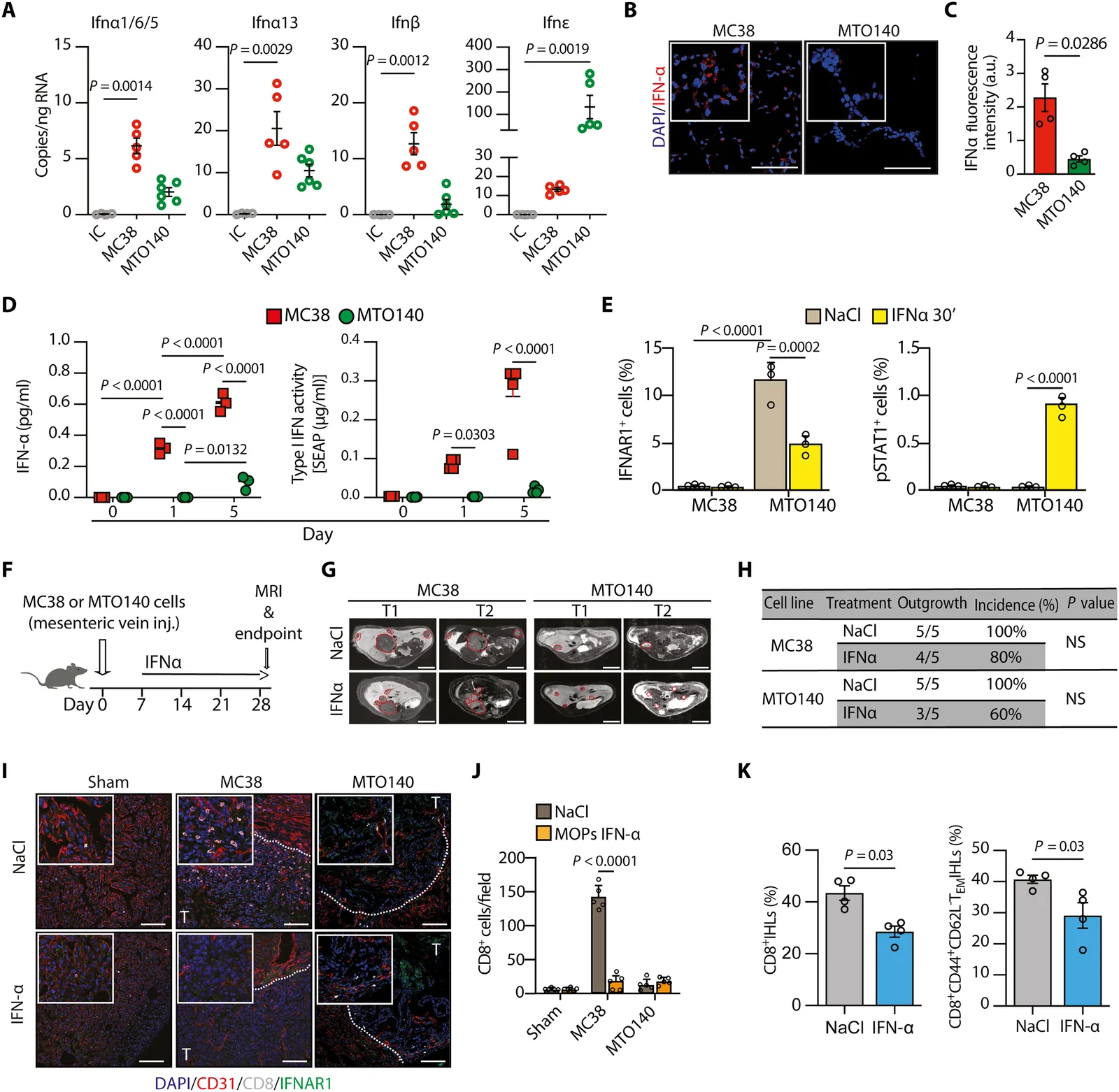
Murine CRC liver metastasis models with distinct tumor-intrinsic IFN-I. (**A**) Absolute IFN-I transcripts measured by digital PCR in MC38, MTO140, and IC epithelial cells (copies/ng RNA; IC, *n* = 4; MC38, *n* = 5; MTO140, *n* = 6). Representative of at least two experiments. (**B** and **C**) Pan–IFN-α staining in MC38 and MTO140 cells and MFI. Scale bar, 50 μm; insets show magnified regions; *n* = 4 fields. (**D**) Secreted pan–IFN-α and functional IFN-I activity in MC38- and MTO140-conditioned media measured by ELISA (*n* = 3) and B16-Blue reporter assay (*n* = 4), respectively. (**E**) Surface IFNAR1^+^ and pSTAT1^+^ cells after 30 min with IFN-α (200 pg/ml) or NaCl (*n* = 3 per condition). (**F**) MC38 (1 × 10^5^) or MTO140 (1.5 × 10^5^) cells were injected through the mesenteric vein into C57BL/6 mice. IFN-α (150 ng/day) or NaCl was delivered by mini-osmotic pumps from day 7 to day 28. (**G**) T1- and T2-weighted MRI at day 27. Dashed lines delineate metastases. Scale bar, 5 mm. (**H**) Tumor incidence (*n* = 5 mice per group). NS, not significant. (**I**) Day 28 liver sections stained for CD31, CD8, IFNAR1, and DAPI. Dashed lines outline tumors. Scale bar, 50 μm; insets show magnified regions. (**J**) CD8^+^ cells per field in Sham, MC38, and MTO140 groups. (**K**) Total and effector-memory CD8^+^ T cells among IHLs from MC38-bearing mice at day 21. Each dot represents one field [(C) and (J)] or mouse (K). Data are means ± SEM [(C) to (E)] or SD [(J) and (K)]. Statistical significance was assessed using Kruskal-Wallis/Dunn (A), two-sided Mann-Whitney [(C) and (K)], two-way ANOVA/Šídák [(D) and (J)], two-way ANOVA with Fisher’s least significant difference (LSD) test (E), or two-sided Fisher’s exact test (H). *P* values are shown.

We next examined receptor availability on tumor cells. Flow cytometric analysis revealed low surface IFNAR1 expression on MC38 cells and comparatively higher levels on MTO140 cells (Fig. 3E). Consistently, MTO140 cells exhibited high levels of signal transducer and activator of transcription 1 (STAT1) phosphorylation following acute IFN-α stimulation, with evident decreasing levels of cell surface IFNAR1 (Fig. 3E), indicating that both models retain IFN-signaling competence in line with basal receptor abundance.

To assess the impact of sustained IFN-α exposure in vivo, mice bearing MC38- or MTO140-derived LMs received continuous IFN-α delivery via mini-osmotic pumps beginning 7 days after tumor injection, at a dose previously shown to prevent perioperative CRC-LM formation (*21*) (Fig. 3F). Treatment was well tolerated, with only a transient and reversible reduction in circulating white blood cell counts (fig. S3D) and did not substantially alter overall tumor burden in either model (fig. S3, E and F). At endpoint, mice received an intraperitoneal bolus of IFN-α and were euthanized 30 min later to assess acute IFN-I responsiveness via STAT1 phosphorylation analysis. MC38 perimetastatic regions displayed a higher fraction of pSTAT1^+^(Tyr^701^) cells compared with MTO140 lesions (fig. S3, G and H). Notably, pSTAT1 signal in MC38-bearing livers localized predominantly to stromal compartments rather than tumor cell–rich cores, consistent with the immune-infiltrated (“hot”) phenotype of MC38 metastases, whereas MTO140 lesions remained comparatively immune poor. At day 28, immunofluorescence analyses revealed that prolonged IFN-α administration led to the complete exclusion of CD8^+^ T cells from MC38 tumor areas, whereas MTO140 tumors exhibited minimal CD8^+^ infiltration at baseline and after treatment (Fig. 3, I and J) consistent with the previously described T cell–excluded phenotype of this model and with transforming growth factor–β (TGFβ)–dependent immunoregulatory mechanisms reported in MTO140 tumors (*44*, *45*).

In contrast, at day 21, intrahepatic leukocyte (IHL) analysis revealed that CD8^+^ T cells were still detectable within the tumor, although at reduced percentage compared with untreated mice (Fig. 3K and fig. S3I). Collectively, these findings indicate that IFN-I^high^, IFNAR1^low^ MC38 tumors establish an IFN-enriched microenvironment in which sustained IFN-I signaling is associated with altered immune spatial organization, particularly CD8^+^ T cell exclusion (*44*, *45*).

### Tumor-derived paracrine IFN-Is reshape the liver metastatic immune landscape

To determine whether tumor-intrinsic IFN-I production influences the surrounding mTME, we next analyzed the transcriptional landscape of whole livers bearing MC38 or MTO140 metastases. These models represent distinct IFN-I contexts, with MC38 exhibiting an IFN-I^high^, IFNAR1^low^ phenotype and MTO140 displaying comparatively lower ligand production and higher receptor availability. MC38 or MTO140 cells were injected via the mesenteric vein into C57BL/6 mice, and livers were harvested at day 26 for bulk RNA sequencing (Fig. 4A).

**Fig. 4.**
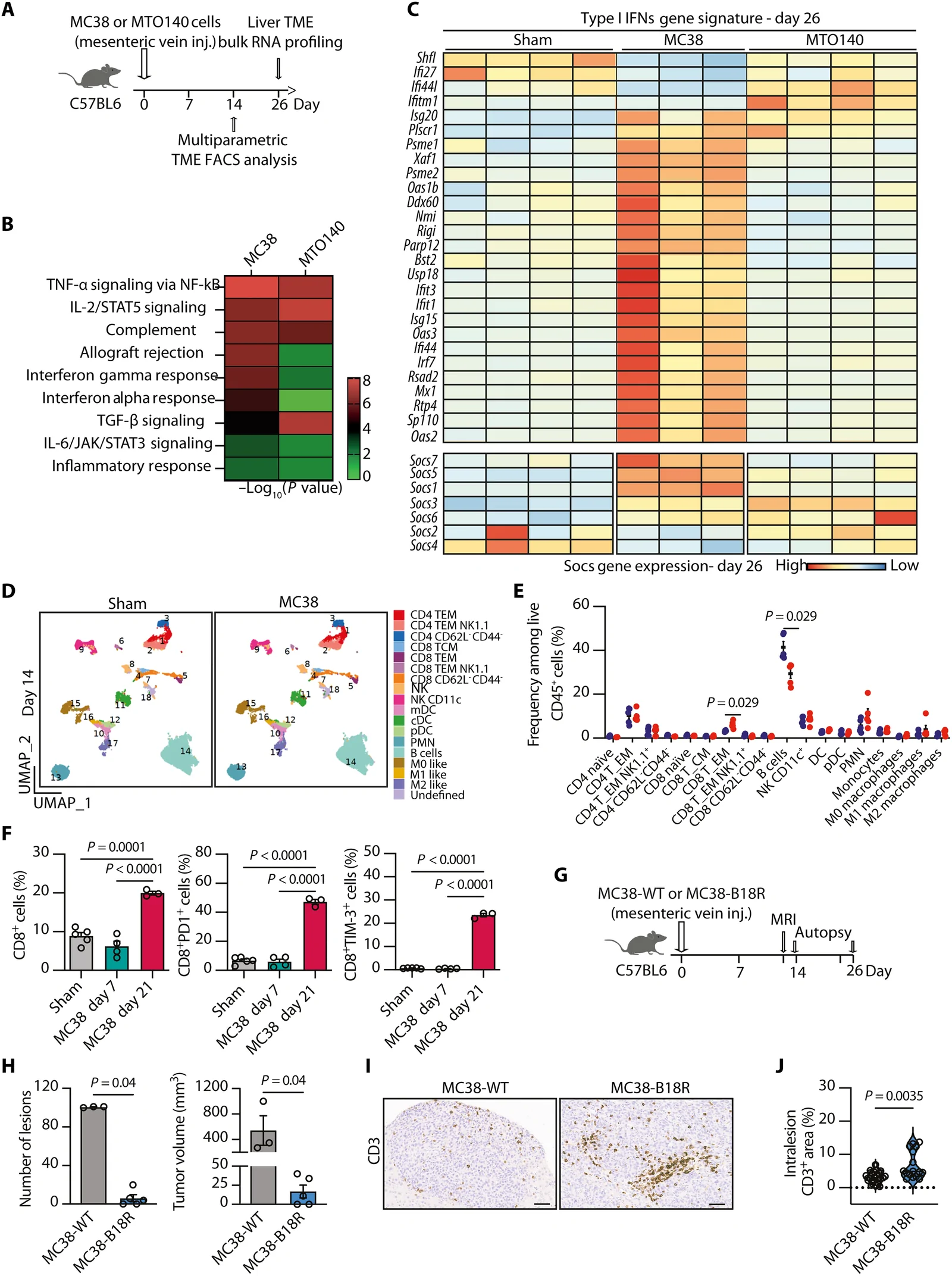
Tumor-derived IFN-Is remodel the immune landscape of CRC-LMs. (**A**) Schematic of the in vivo experimental design. MC38 (1 × 10^5^) and MTO140 (1.5 × 10^5^) cells were injected via the mesenteric vein into C57BL/6 mice (sham, *n* = 4; MC38, *n* = 3; MTO140, *n* = 4). Mice were analyzed at day 14 by immune profiling or at day 26 by bulk RNA-seq. (**B**) Pathway enrichment of up-regulated transcripts in MC38- and MTO140-bearing livers at day 26, including interferon-α, interferon-γ, and TGFβ signaling. (**C**) Relative expression of a 27-gene IFN-I signature and Socs genes in sham, MC38-bearing, and MTO140-bearing livers. Values were derived from log2-transformed RPKM and scaled by gene. (**D**) UMAP clustering of live CD45^+^ IHLs from sham and MC38-bearing livers at day 14. (**E**) Frequencies of the indicated immune-cell populations among live CD45^+^ cells (*n* = 5 mice per group). (**F**) Frequencies of total CD8^+^, CD8^+^PD-1^+^, and CD8^+^TIM-3^+^ T cells in sham mice and MC38-bearing mice at days 7 and 21 (Sham, *n* = 5; MC38 day 7, *n* = 4; MC38 day 21, *n* = 3). (**G**) Schematic of the in vivo experimental design. MC38-WT or MC38-B18R cells (2 × 10^5^) were injected via the mesenteric vein (WT, *n* = 3; B18R, *n* = 5). (**H**) Number of metastatic lesions and total tumor volume at day 14 by MRI. (**I**) Representative CD3 IHC of MC38-WT and MC38-B18R lesions. Scale bars, 100 μm. (**J**) Intralesional CD3^+^ cells. Each dot represents one mouse [(E), (F), and (H)]; bars indicate means ± SEM. Statistical significance was assessed using two-sided Mann-Whitney tests with FDR correction (E), one-way ANOVA with Tukey’s correction (F), or two-sided Mann-Whitney tests [(H) and (J)]. *P* values are shown. NS, not significant.

Principal components analysis demonstrated clear separation of sham, MC38-bearing, and MTO140-bearing livers, indicating distinct transcriptional programs (fig. S4A). Differential expression analysis [false discovery rate (FDR) < 0.01; |log_2_FC| > 1] identified 590 up-regulated and 1067 down-regulated genes in MC38-bearing livers, compared with 317 up-regulated and 111 down-regulated genes in MTO140-bearing livers relative to sham controls (fig. S4B). Pathway enrichment analysis revealed prominent activation of IFN-α and IFN-γ response signatures in MC38-bearing livers, whereas TGFβ signaling was more enriched in MTO140-bearing livers (Fig. 4B), consistent with recent observations (*44*, *45*). To assess whether this transcriptional profile reflected previously described IFN-I–associated programs in CRC-LMs, we examined a published IFN-α gene signature identified in a subset of patients with CRC-LMs (*26*). A subset of 27 murine IFN-I genes derived from this signature was strongly enriched in MC38-bearing livers, whereas only 4 were preferentially up-regulated in MTO140-bearing livers (Fig. 4C, top). Because sustained cytokine signaling can induce negative-feedback mechanisms, we next examined the expression of suppressor of cytokine signaling (SOCS) family members (*46*). MC38-bearing livers showed increased expression of selected SOCS genes, particularly *Socs1*, *Socs5*, and *Socs7*, compared with sham and MTO140-bearing livers, whereas other family members displayed distinct, tumor model–specific patterns (Fig. 4C, bottom). In parallel, analysis of immune-inhibitory genes revealed distinct transcriptional profiles in the two models: *Cd274*, *Havcr2*, *Pdcd1*, *Entpd1*, and *Ctla4* were more prominently expressed in MC38-bearing livers, whereas *Pdcd1lg2* and *Tigit* were relatively increased in MTO140-bearing livers (fig. S4C). Together, these data indicate that IFN-I^high^ tumors are associated with a robust IFN-I transcriptional program in the liver metastatic niche and accompanied by the induction of selected cytokine-feedback regulators and a distinct immunoregulatory gene-expression profile.

We next examined whether these transcriptional differences were accompanied by changes in the hepatic immune landscape. Immunohistochemical analysis demonstrated increased intratumoral CD3^+^ T cell infiltration in MC38 tumors at day 21 compared with MTO140 tumors, which displayed a comparatively “cold” phenotype, whereas F4/80^+^ myeloid-cell abundance was similar between the two models (fig. S4, D and E). Flow cytometric profiling of the intrahepatic immune infiltrate at day 14 after intramesenteric injection revealed remodeling of the immune landscape in MC38-bearing livers compared with sham controls. Unsupervised uniform manifold approximation and projection (UMAP) clustering of live CD45^+^ cells identified distinct lymphoid and myeloid populations (Fig. 4D and fig. S4F), and their quantification demonstrated enrichment of CD8^+^ effector memory T (T_EM_) cells together with a relative reduction in B cell abundance, whereas the other populations analyzed did not differ substantially (Fig. 4E).

To assess the impact of sustained IFN-I exposure on CD8^+^ T cell phenotype, we analyzed the intrahepatic immune infiltrate at early (day 7) and late (day 21) time points following MC38 injection. Although overall CD8^+^ T cell percentage increased over time, these cells progressively acquired an activated and dysfunction/exhaustion-associated phenotype, characterized by increased PD-1 expression (∼45 to 50%) and the emergence of TIM-3^+^ subsets (∼20 to 25%) at day 21 (Fig. 4F). Collectively, these findings indicate that IFN-I^high^, IFNAR1^low^ CRC-LMs are associated with induction of an IFN-I transcriptional program in the liver mTME, selective remodeling of the intrahepatic immune landscape, and progressive alteration of the CD8^+^ T cell compartment.

To directly test whether tumor-derived IFN-Is contribute to these transcriptional and immune alterations, we generated MC38 cells expressing B18R, a soluble viral decoy receptor that neutralizes extracellular IFN-Is (fig. S4H) (*47*). In vitro, B18R expression did not reduce intracellular IFN-α protein levels (fig. S4, I and J) but attenuated downstream signaling responses, as indicated by reduced *Isg15* induction following IFN-α stimulation (fig. S4K). To evaluate the consequences of local extracellular IFN-I neutralization in vivo, we injected C57BL/6 mice via the mesenteric vein with MC38-WT (wild-type) or MC38-B18R cells. Hepatic metastatic burden assessed by magnetic resonance imaging (MRI) revealed a marked reduction in the number of total metastatic lesions and overall volume of hepatic lesions in mice bearing MC38-B18R tumors compared with mice bearing MC38-WT tumors (Fig. 4H). MC38-B18R-bearing livers also exhibited reduced expression of representative IFN-I transcripts (*Ifna2*, *Ifna4*, and *Ifnb1*) and ISGs (*Ifit1*, *Isg15*, and *Ifit3*) in whole liver tissue compared with MC38-WT-bearing livers (fig. S4L). Consistent with a functional effect on the immune microenvironment, immunohistochemical analysis revealed a substantially higher proportion of intralesional CD3^+^ cells in MC38-B18R tumors than in MC38-WT tumors (Fig. 4, I and J). Thus, B18R-mediated neutralization of extracellular IFN-Is was associated with both reduced metastatic burden and increased T cell accumulation within metastatic lesions. Consistent with previous evidence linking chronic IFN-I signaling to T cell dysfunction and adaptive immunoregulatory programs (*22*, *24*, *30*), our data support a contributory role for tumor-derived IFN-Is in shaping the transcriptional landscape of the liver mTME and suggest that sustained extracellular IFN-I signaling may limit intratumoral T cell accumulation.

### Tumor-derived IFN-Is impair CD8^+^ T cell persistence through IFNAR1

To assess whether sustained IFN-I signaling directly compromises CD8^+^ T cell fitness, we isolated naïve CD8^+^ OT-I T cells from OT-I transgenic mice and differentiated them in vitro with SIINFEKL peptide to generate effector memory-like OT-I CD8^+^ T cells (CD44^high^ CD62L^low^ OT-I T_EM_) (Fig. 5, A and B, and fig. S5A). This system provides a defined antigen-specific CD8^+^ cell model to assess the direct effects of IFN-I exposure independent of additional tumor-derived cues. Acute IFN-α stimulation induced rapid STAT1 phosphorylation and reduced surface IFNAR1 expression in OT-I T_EM_ cells (Fig. 5, C to F, and fig. S5B) consistent with ligand-induced receptor down-regulation.

**Fig. 5.**
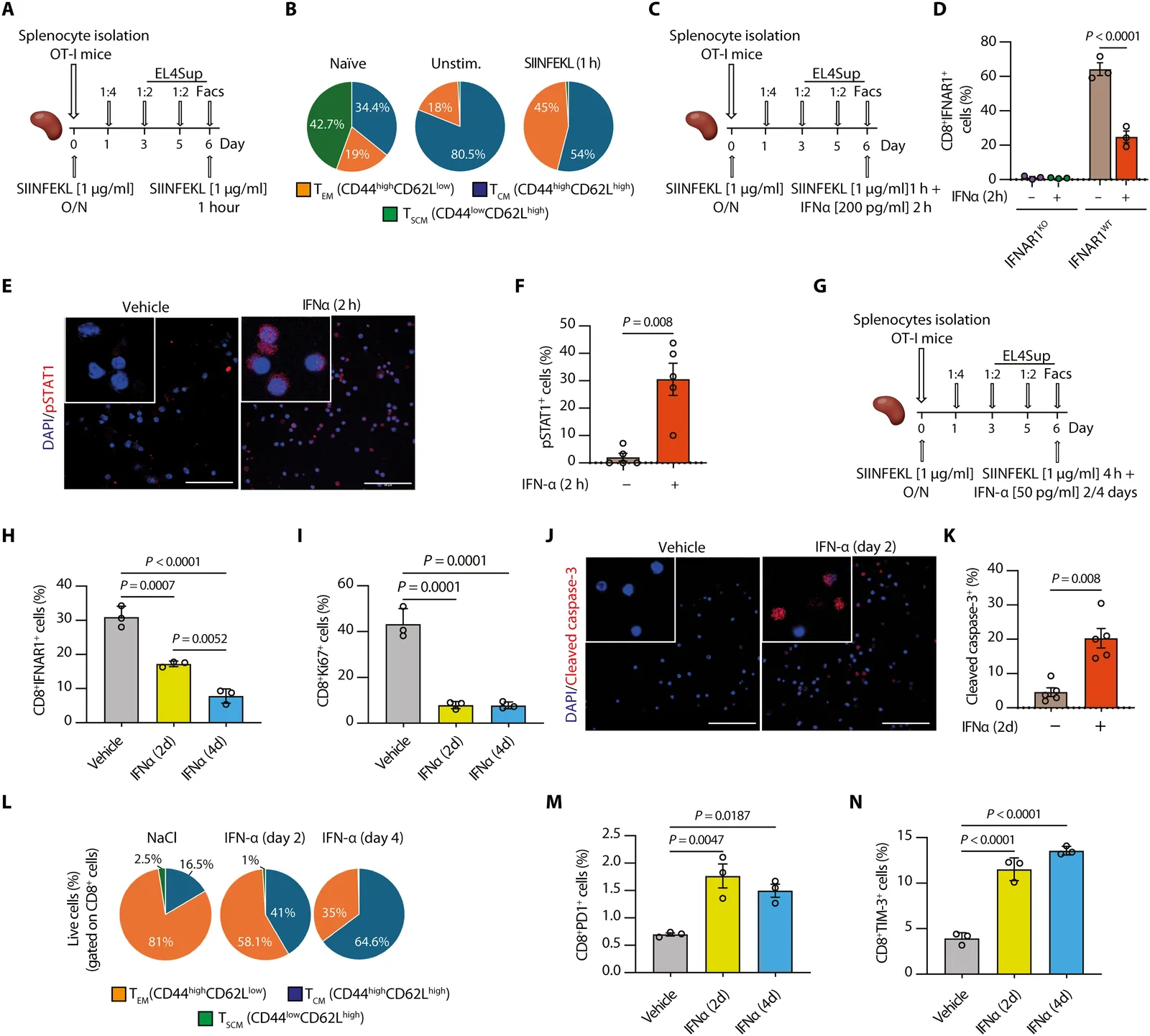
Chronic CRC-derived IFN-I signaling impairs CD8^+^ T cell persistence and function. (**A**) Schematic of the antigen-driven activation and expansion of OT-I CD8^+^ T cells to generate effector OT-I cells. OT-I splenocytes were activated overnight with SIINFEKL (1 μg/ml) and expanded in 2.5% EL-4 supernatant to generate effector cells. Unless otherwise indicated, analyses were performed on live CD8^+^ OT-I cells. (**B**) Distribution of effector-memory (T_EM_; CD8^+^CD44^high^CD62L^Low^), T cell memory (T_CM_; CD8^+^CD44^high^CD62L^high^), and stem-cell-memory (T_SCM_; CD8^+^CD44^low^CD62L^high^) subsets in naïve, unstimulated, and SIINFEKL-restimulated cells at day 6. (**C**) Day-6 effector OT-I cells were restimulated with SIINFEKL for 1 hour and exposed to IFN-α (200 pg/ml) for 2 hours. (**D**) Surface IFNAR1 after acute IFN-α or vehicle treatment; IFNAR1^KO^ splenocytes served as staining controls. (**E** and **F**) Representative pSTAT1(Tyr701) immunofluorescence and percentage of pSTAT1-positive cells after acute IFN-α exposure. Scale bar, 50 μm; insets show magnified regions; five fields per condition. (**G**) OT-I cells were activated and expanded for 6 days, restimulated with SIINFEKL for 4 hours, and cultured with IFN-α (50 pg/ml) or vehicle for 2 or 4 days. (**H** and **I**) Frequencies of IFNAR1^+^ and Ki-67^+^ cells after chronic IFN-α exposure. (**J** and **K**) Representative cleaved caspase-3 immunofluorescence and percentage of positive cells after 2 days of treatment. Scale bar, 50 μm; insets show magnified regions; five fields per condition. (**L**) Distribution of T_EM_, T_CM_, and T_SCM_ subsets after 2 or 4 days of IFN-α or vehicle treatment. (**M** and **N**) Frequencies of PD-1^+^ and TIM-3^+^ cells after chronic IFN-α exposure. Data are means ± SEM. Statistical significance was assessed using one-way ANOVA with Tukey’s multiple-comparisons test [(D), (H), (I), (M), and (N)] or two-sided Mann-Whitney tests [(F) and (K)]. *P* values are shown. h, hour.

To model sustained exposure, we cultured OT-I T_EM_ cells with low-dose IFN-α (50 pg/ml) for 2 or 4 days. Chronic IFN-α treatment resulted in persistent IFNAR1 down-regulation (Fig. 5, G and H), decreased proliferation (reduced CD8^+^Ki-67^+^ fraction; Fig. 5I), and increased apoptosis (cleaved caspase-3^+^ cells; Fig. 5, J and K), accompanied by reduced cell recovery/viability in CD8^+^ T_EM_ populations (Fig. 5L) and acquisition of dysfunctional phenotypes. In particular, prolonged IFN-α exposure increased the proportion of TIM-3^+^ OT-I CD8^+^ cells (∼10%) and, to a lesser extent, PD-1^+^ OT-I CD8^+^ cells (∼2%) (Fig. 5, M and N), indicating that sustained IFN-I signaling promotes features consistent with T cell dysfunction. Collectively, these data demonstrate that sustained exposure to tumor-derived IFN-Is is sufficient to promote IFNAR1 modulation and impair CD8^+^ T cell persistence, while driving distinct dysfunctional phenotypes depending on the tumor context.

### IFNAR1 stabilization preserves CD8^+^ T cell fitness in response to IFN-α exposure

Consistent with our in vitro findings that sustained IFN-α exposure promotes IFNAR1 down-regulation and CD8^+^ T cell dysfunction (Fig. 5), we next examined whether pharmacological stabilization of IFNAR1 could mitigate these effects in vivo. C57BL/6 mice bearing MC38 LMs received vehicle, continuous IFN-α via mini-osmotic pumps starting 7 days after tumor injection, either alone or in combination with the p38 mitogen-activated protein kinase (MAPK) inhibitor LY2228820 and the PKD/TGFβ receptor I inhibitor SD-208 (LY/SD-208), a regimen previously shown to stabilize IFNAR1 on cell surface (Fig. 6A) (*34*). All treatments were well tolerated and had only modest effects on circulating white blood cell counts, which remained within the physiological range throughout therapy (fig. S6A). Neither IFN-α monotherapy nor LY/SD-208 alone substantially altered metastatic burden, as assessed by MRI-based lesion number and volume quantification at day 28 (Fig. 6, B and C, and fig. S6B). By contrast, combined IFN-α and LY/SD-208 treatment markedly reduced metastatic outgrowth and tumor incidence (Fig. 6, B and C, and fig. S6B). Histologic analysis of liver lesions revealed decreased proliferative index (Ki67; Fig. 6D and fig. S6C), increased CD3^+^ T cell infiltration, and enhanced cleaved caspase-3 staining in the combination group relative to controls (Fig. 6, D to F). This response was associated with increased frequencies of circulating CD8^+^IFNAR1^+^ T cells (Fig. 6G).

**Fig. 6.**
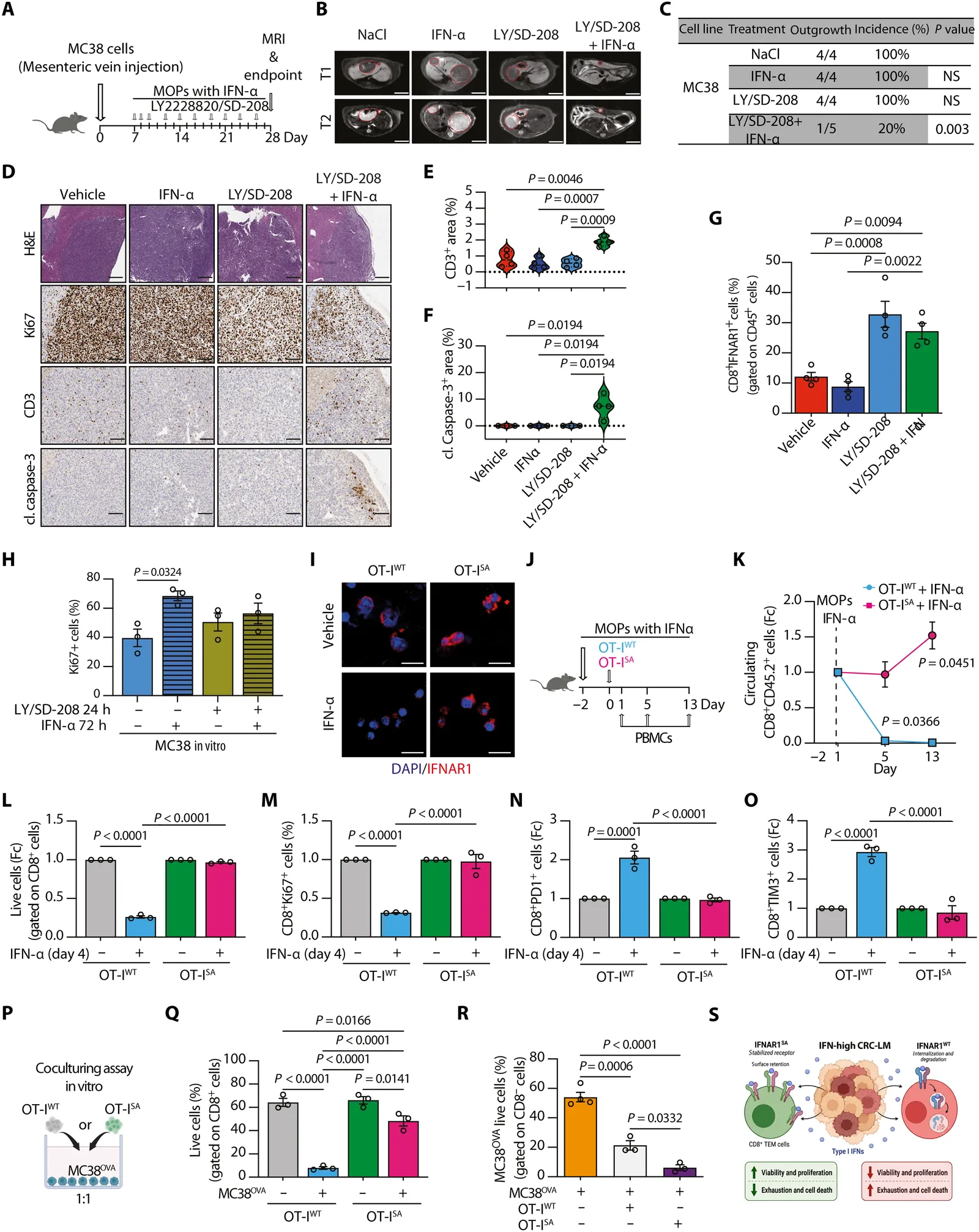
Pharmacological and genetic IFNAR1 stabilization preserves CD8^+^ T cell fitness during chronic IFN-α exposure. (**A**) Schematic of the in vivo treatment design. MC38-bearing mice received NaCl, IFN-α by mini-osmotic pumps, LY2228820 plus SD-208 (LY/SD-208), or LY/SD-208 plus IFN-α from day 7 to day 28. (**B**) Representative T1- and T2-weighted MRI. Dashed lines delineate metastases. Scale bar, 5 mm. (**C**) Metastatic outgrowth incidence: NaCl, 4/4; IFN-α, 4/4; LY/SD-208, 4/4; LY/SD-208 + IFN-α, 1/5. (**D**) Hematoxylin and eosin (H&E) and Ki-67, CD3, and cleaved caspase-3 staining of liver lesions. Scale bars, 500 μm (H&E) and 200 μm (IHC). (**E** and **F**) CD3^+^ and cleaved caspase-3^+^ areas. Each dot represents the mean across all lesions from one mouse. (**G**) Circulating CD8^+^IFNAR1^+^ cells on day 14 after 7 days of treatment. Each dot represents one mouse. (**H**) Ki-67^+^ MC38 cells after 72-hour IFN-α exposure, with or without 24-hour LY/SD-208 pretreatment (*n* = 3). (**I**) IFNAR1 staining in OT-I^WT^ and OT-I^SA^ CD8^+^ T cells after vehicle or chronic IFN-α exposure. Scale bar, 10 μm. (**J**) IFN-α–treated recipients received effector OT-I^WT^ or OT-I^SA^ CD8^+^ T_EM_ cells 2 days after MOP implantation (*n* = 4 mice per group). (**K**) Fold change in circulating transferred OT-I cells, normalized to day 1 after adoptive transfer. (**L** to **O**) Fold change in live, Ki-67^+^, PD-1^+^, and TIM-3^+^ OT-I^WT^ and OT-I^SA^ cells after 4 days of IFN-α in vitro (*n* = 3). (**P**) OT-I^WT^ or OT-I^SA^ cells were cocultured with MC38^OVA^ cells at a 1:1 ratio. (**Q** and **R**) T cell viability and tumor cell survival after coculture (*n* = 3). (**S**) Model of IFNAR1 stabilization in IFN-high CRC-LMs. Created with FigureLabs.ai. Quantitative data are means ± SEM where applicable. Statistical significance was assessed using Fisher’s exact test (C), one-way ANOVA with Tukey’s correction [(E) to (H), (L) to (O), and (R)], a mixed-effects model with Šídák’s multiple-comparisons test (K), or one-way ANOVA with Holm-Šídák’s multiple-comparisons test (Q). *P* values are shown.

To determine whether LY/SD-208 exerted a direct antitumor effect on MC38 cells, we examined tumor cell IFNAR1 expression and proliferation in vitro. Pretreatment of MC38 with LY/SD-208 increased surface IFNAR1 expression, which was further enhanced following IFN-α stimulation (fig. S6D). However, under prolonged IFN-α exposure, LY/SD-208 did not alter the percentage of Ki-67^+^ MC38 cells compared with the corresponding control conditions (Fig. 6H), arguing against a direct antiproliferative effect of the inhibitors on tumor cells. This interpretation is also consistent with the lack of in vivo efficacy of LY/SD-208 administered alone (Fig. 6, B to F, and fig. S6, B and C). Thus, antitumor activity was observed only when LY/SD-208 was combined with sustained IFN-α exposure in immunocompetent mice. To further assess the contribution of host IFNAR1 signaling, LY/SD-208 was evaluated in WT and global *Ifnar1*^KO^ recipient mice. The effects of LY/SD-208 on metastatic burden were attenuated in *Ifnar1*^KO^ compared with WT recipients (fig. S6E), suggesting that its therapeutic activity depends on host IFNAR1 expression.

Consistently, at the cellular level, OT-I CD8^+^ T_EM_ cells (fig. S6F), pretreated in vitro with LY/SD-208 and subsequently exposed to low-dose IFN-α (50 pg/ml) for 2 days displayed preserved surface IFNAR1 expression and enhanced pSTAT1 activation compared with cells exposed to IFN-α alone (fig. S6, G and H). To genetically corroborate these findings, we used *Ifnar1*^SA^ knock-in mice harboring a serine-to-alanine substitution that prevents IFNAR1 phosphorylation and internalization, thereby stabilizing the receptor at the cell surface (*32*). OT-I T cells derived from these mice (OT-I^SA^) exhibited higher baseline IFNAR1 surface expression and preserved cytotoxic features relative to OT-I^WT^ controls (Fig. 6I and fig. S6I). Acute IFN-α stimulation induced receptor internalization in OT-I^WT^ but not OT-I^SA^ cells (fig. S6J). Notably, OT-I^SA^ cells exhibit enhanced pSTAT1 responsiveness (fig. S6K).

We next tested whether IFNAR1 stabilization enhances T cell persistence under systemic IFN-α exposure in vivo. To isolate the effects of sustained IFN-α independent of tumor-derived variables, CD45.1/CD45.2 recipient mice were treated with IFN-α for 2 days before adoptive transfer of in vitro–activated OT-I^WT^ or OT-I^SA^ effector cells (Fig. 6J). Under continuous IFN-α exposure, circulating OT-I^WT^ cells progressively declined, whereas OT-I^SA^ cells expanded and persisted over 13 days (Fig. 6K). In vitro, prolonged IFN-α exposure (4 days) substantially reduced viability and proliferation of OT-I^WT^ cells, while OT-I^SA^ cells were protected (Fig. 6, L and M, and fig. S6L). Genetic stabilization of IFNAR1 also prevented induction of exhaustion-associated markers PD-1 and TIM-3 (Fig. 6, N and O) and preserved IFN-γ production following antigen restimulation (fig. S6M).

Last, in antigen-specific coculture assays (Fig. 6P) using MC38 cells engineered to express ovalbumin (OVA) (MC38^OVA^; fig. S6, N and O), OT-I^SA^ CD8^+^ T cells exhibited substantially superior viability (Fig. 6Q) and enhanced tumor cell elimination in vitro compared with OT-I^WT^ cells (Fig. 6R). These data demonstrate that pharmacologic or genetic stabilization of IFNAR1 preserves CD8^+^ T cell viability and effector function during sustained IFN-α exposure, enhancing persistence and antitumor activity in IFN-rich environments (Fig. 6S).

### Adoptive transfer of IFNAR1^SA^ OT-I T cells eliminates established MC38^OVA^ LMs and confers durable protection

Having established that pharmacologic and genetic stabilization of IFNAR1 preserves CD8^+^ T cell fitness under sustained IFN-I exposure, we next asked whether this advantage translates into improved therapeutic efficacy in ACT against CRC-LMs. To test this, we injected MC38^OVA^ cells via the mesenteric vein and performed adoptive transfer of in vitro–activated OT-I^WT^ or OT-I^SA^ CD8^+^ T_EM_ cells after 7 or 14 days. When ACT was administered 7 days after tumor seeding (Fig. 7A), before overt radiographic progression and at a stage when tumor-derived IFN-Is remain low or undetectable, both OT-I^WT^ and OT-I^SA^ cells showed comparable efficacy in controlling disease progression. MRI at day 28 showed a complete absence of detectable lesions in both treatment groups compared with NaCl controls, which developed extensive LMs (fig. S7A). Quantification confirmed a marked reduction in lesion number and total metastatic volume (Fig. 7, B and C), translating into prolonged survival in both OT-I–treated cohorts (Fig. 7D). These findings indicate that IFNAR1 stabilization does not confer additional therapeutic benefit in early, micrometastatic disease characterized by limited IFN-I exposure, suggesting that the advantage of receptor stabilization may emerge specifically in IFN-rich contexts.

**Fig. 7.**
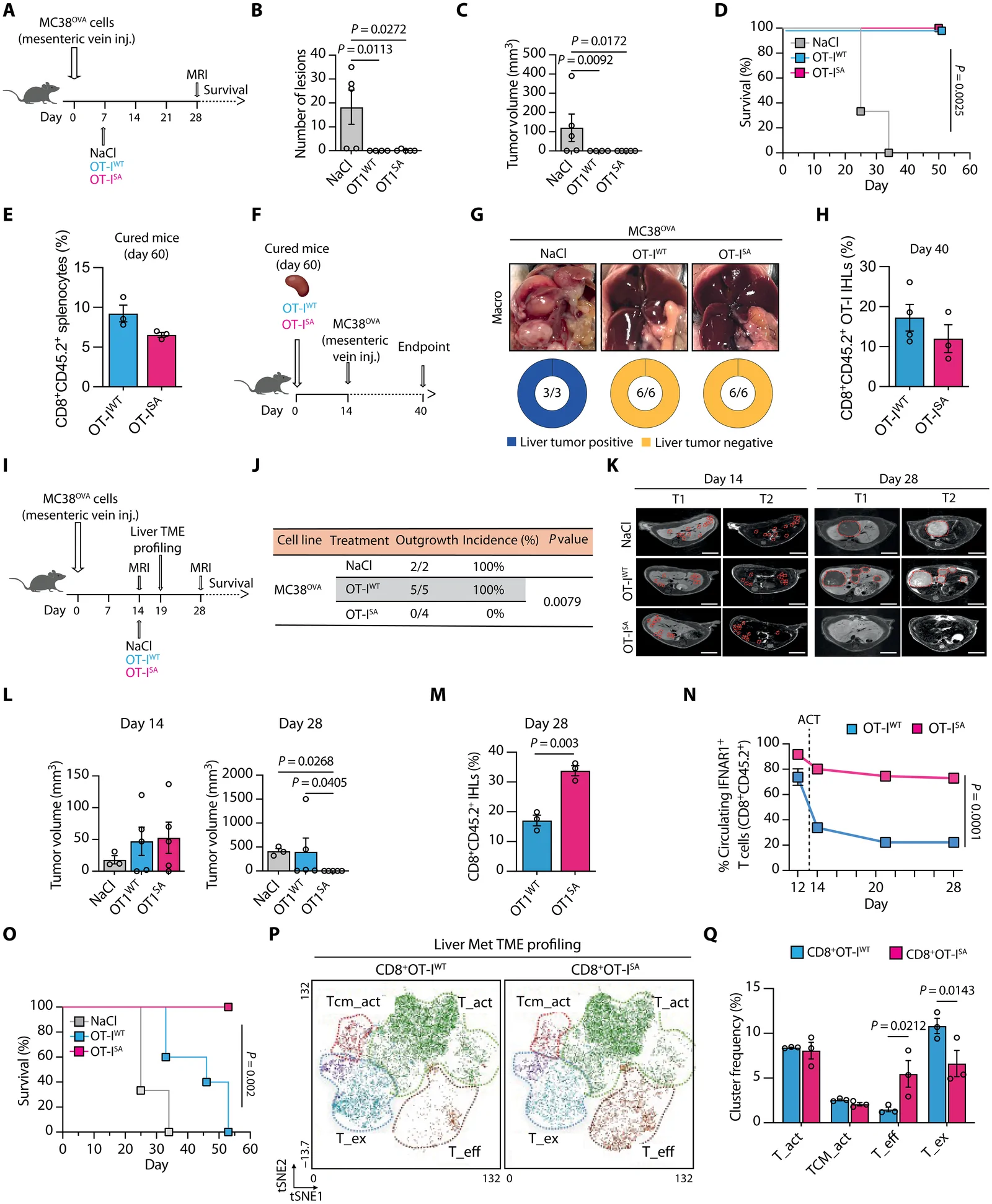
IFNAR1 stabilization enhances CD8^+^ T cell–mediated control of IFN-I^high^ CRC-LMs and promotes durable antitumor immunity. (**A**) Experimental design for early ACT: MC38^OVA^ cells (3 × 10^5^) were injected via the mesenteric vein on day 0; mice received NaCl, OT-I^WT^, or OT-I^SA^ effector CD8^+^ cells on day 7 and were followed for survival. (**B** and **C**) Number of liver lesions and metastatic volume at day 28 by MRI. (**D**) Kaplan-Meier survival analysis. (**E**) Splenic CD8^+^CD45.2^+^ OT-I cells in cured mice at day 60. (**F**) Splenocytes from mice cured by OT-I^WT^ or OT-I^SA^ treatment were transferred into naïve recipients, which were challenged 14 days later with MC38^OVA^ cells. (**G**) Representative livers and tumor incidence at endpoint. (**H**) CD8^+^CD45.2^+^ OT-I cells among intrahepatic lymphocytes at day 40. (**I**) Experimental design for ACT in the established-disease setting: MC38^OVA^-bearing mice received NaCl, OT-I^WT^, or OT-I^SA^ cells on day 14; liver immune profiling was performed on day 19 and survival monitored. (**J**) Metastatic outgrowth incidence at day 28. (**K**) Representative T1- and T2-weighted MRI at days 14 and 28. Dashed lines delineate metastases. Scale bar, 5 mm. (**L**) Total tumor volume at days 14 and 28. (**M**) CD8^+^CD45.2^+^ intrahepatic OT-I cells at day 28. (**N**) Longitudinal circulating IFNAR1^+^CD8^+^CD45.2^+^ OT-I cells after ACT. (**O**) Kaplan-Meier survival analysis. (**P** and **Q**) FlowSOM/tSNE analysis and frequencies of activated (T_act), activated-memory (T_cm_act), effector (T_eff), and exhausted (T_ex) T cell clusters in LMs at day 19. Each dot represents one mouse; bars indicate means ± SEM. Statistical significance was assessed using Kruskal-Wallis tests with Dunn’s correction [(B), (C), and (L)], log-rank tests [(D) and (O)], two-sided Mann-Whitney tests [(E) and (H)], Fisher’s exact test (J), an unpaired t test with Welch’s correction (M), a mixed-effects model with Tukey’s multiple-comparisons test (N), or two-way ANOVA with Šídák’s multiple-comparisons test (Q). *P* values are shown.

Although *Ifnar1*^SA^ stabilized mice have not shown an overt spontaneous senescence phenotype, sustained IFN-I signaling has been linked to cumulative stress responses and impaired long-term T cell fitness (*29*). Because OT-I cells are primed ex vivo and subsequently function in an IFN-I^high^ mTME during tumor clearance, we next asked whether IFNAR1 stabilization affects durability and recall competence under conditions of heightened IFN-I exposure. To address this, spleens were harvested from mice cured by day 7 ACT at day 60, and donor OT-I cells were quantified, revealing comparable numbers of OT-I^WT^ and OT-I^SA^ CD8^+^ T_EM_ cells (Fig. 7E). Equal numbers of recovered OT-I^WT^ and OT-I^SA^ CD8^+^ T_EM_ cells were then transferred into naïve CD45.1/CD45.2 C57BL/6 recipients to decouple T cell intrinsic persistence from the cured donor environment and directly assess recall capacity. After a 2-week equilibration period, recipients were challenged by mesenteric vein injection of MC38^OVA^ cells (Fig. 7F). At necropsy, both cohorts remained disease-free (Fig. 7G) and exhibited comparable frequencies of donor OT-I cells in the blood (fig. S7B) and among intrahepatic lymphocytes, quantified as CD8^+^CD45.2^+^OT-I^+^ IHLs at day 40 (Fig. 7H). These results indicate that IFNAR1 stabilization does not measurably impair long-term persistence or recall-mediated protection.

We also tested ACT in the setting of established metastases, where sustained IFN-I exposure and T cell dysfunction are expected to be more pronounced. OT-I^WT^ or OT-I^SA^ CD8^+^ T_EM_ cells were transferred 14 days after MC38^OVA^ injection (Fig. 7I). Longitudinal MRI analysis at day 28 revealed complete clearance of LMs in mice treated with OT-I^SA^ cells, whereas mice receiving OT-I^WT^ cells remained tumor positive, with lesion numbers and volumes comparable to NaCl controls (Fig. 7, J to L, and fig. S7C). OT-I^SA^ CD8^+^ T_EM_ cells persisted at higher numbers within livers (Fig. 7M) and were maintained in the circulation through day 28, whereas transferred OT-I^WT^ CD8^+^ T_EM_ cells progressively declined over time (Fig. 7N).

Consistent with these findings, although OT-I^WT^ ACT improved survival relative to NaCl controls, only OT-I^SA^ therapy eliminated LMs and substantially extended long-term survival (Fig. 7O). To link improved tumor control with T cell state within the mTME, we profiled the liver mTME 5 days after ACT (day 19) by multiparametric flow cytometry of IHLs (Fig. 7P and fig. S7D). Compared with OT-I^WT^ transfer, OT-I^SA^ CD8^+^ T_EM_ cell transfer resulted in a notable increase in the effector-like (T_EM_) cluster and a reduction in the exhausted-like (T_EX_) cluster (Fig. 7, P and Q), consistent with enhanced persistence and reduced exhaustion in vivo within an IFN-rich metastatic niche. Collectively, these data indicate that genetically preventing IFNAR1 internalization preserves tumor-specific CD8^+^ T cells from dysfunction in IFN-I–rich environments, thereby conferring a functional advantage for eradication of established CRC-LMs and durable protection in this model.

To determine whether exogenous IFN-α could further enhance ACT when IFNAR1 was genetically stabilized in tumor-specific CD8^+^ T cells, we first identified a subcurative dose of OT-I^SA^ cells in mice bearing established MC38^OVA^ LMs. Dose titration confirmed the therapeutic efficacy of high-dose OT-I^SA^ ACT observed in Fig. 7, I to L and identified an intermediate dose that conferred only partial protection. This intermediate dose was selected for subsequent combination studies (fig. S7E). Mice bearing established MC38^OVA^ LMs received OT-I^SA^ cells alone or in combination with continuous IFN-α administration (fig. S7F). At day 28, 60% of mice receiving subcurative OT-I^SA^ ACT alone, and 40% of those receiving OT-I^SA^ ACT plus IFN-α remained free of detectable metastatic outgrowth, whereas none of the NaCl-treated controls did (fig. S7G). Despite the lack of additional benefit from IFN-α, both treatment regimens substantially reduced metastatic lesion number and total tumor volume compared with NaCl treatment (fig. S7, H to J). Tumor burden in both the treated groups remained close to pretreatment values measured at day 14, whereas NaCl-treated mice exhibited marked metastatic progression by day 28.

Consistent with these findings, immunohistochemical analysis further showed that both treatment regimens substantially increased intralesional CD3^+^ area compared with NaCl-treated controls with a numerical trend toward greater CD3^+^ accumulation following the addition of continuous IFN-α (fig. S7, K to L). Thus, even at a subcurative dose, OT-I^SA^ ACT prevented detectable metastatic outgrowth in a proportion of mice, markedly restrained the progression of residual disease, and promoted intralesional T cell accumulation. These therapeutic effects were maintained during continuous IFN-α administration although IFN-α did not provide an additional measurable improvement in tumor control under these experimental conditions.

### IFNAR1 stabilization enhances anti-CEA CAR T cell persistence and therapeutic efficacy in IFN-I^high^ CRC-LMs

Having shown that IFNAR1 stabilization preserves tumor-specific CD8^+^ T cell fitness and improves ACT efficacy in IFN-I^high^ MC38^OVA^ metastases, we next asked whether this principle extends to a clinically relevant CAR T platform and whether its benefit depends on the IFN-I context of the mTME. T cells from IFNAR1^WT^ or IFNAR1^SA^ donors were engineered to express a CAR targeting hCEA, generating hCEA–CAR T^WT^ and hCEA–CAR T^SA^ cells. Following activation, T cells were transduced with a retroviral vector containing a green fluorescent protein (GFP) reporter downstream of CEA-CAR coding sequence to monitor T cell transduction (fig. S8, A and B) and track them upon injection in vivo. Both populations expanded efficiently and displayed comparable CAR transduction efficiency (∼65% versus ∼59%; fig. S8, C and D).

To enable longitudinal monitoring of tumor burden, MC38 cells were engineered to express hCEA together with a secreted Lucia luciferase reporter (MC38-hCEA^LuciaLuc^), allowing minimally invasive quantification of metastatic growth via serum luciferase measurement (fig. S8, E and F) (*48*). In vitro, sorted hCEA^+^ MC38-hCEA^LuciaLuc^ cells were equivalently recognized and killed by both CAR T products and induced comparable IFN-γ release (Fig. 8A), indicating similar baseline cytotoxic capacity. MC38-hCEA^LuciaLuc^ cells were then injected via the mesenteric vein to establish IFN-I^high^ LMs (Fig. 8B and fig. S8G). Following lymphodepletion (4.5 Gy) and randomization based on serum luciferase levels (Fig. 8C), mice received equal numbers (10^7^) of hCEA–CAR T^WT^ or hCEA–CAR T^SA^ cells. Although both CAR T populations engrafted similarly and were present at comparable levels in circulation at day 9 (fig. S8H), hCEA–CAR T^SA^ treatment resulted in substantially superior control of metastatic progression, as demonstrated by longitudinal serum luciferase measurements (Fig. 8C) and MRI at day 22 (Fig. 8, D and E), which revealed reduced lesion number and tumor volume. Histologic analysis at day 22 showed that vehicle- and hCEA–CAR T^WT^–treated metastases remained highly proliferative (Ki67^+^) and largely devoid of CD3^+^ infiltration, whereas the few lesions detected in hCEA–CAR T^SA^–treated mice exhibited increased CD3^+^ and GFP^+^ T cell accumulation and reduced proliferative index (fig. S8, I to K). Quantification of IHLs confirmed enhanced CD8^+^ CAR T infiltration in the hCEA–CAR T^SA^ group, while CD4^+^ CAR T representation remained low across conditions (Fig. 8F). Immunofluorescence further demonstrated greater intratumoral accumulation of GFP^+^ hCEA–CAR T^SA^ cells, with at least twofold enrichment relative to hCEA–CAR T^WT^ cells (Fig. 8, G and H). In contrast to CAR T^SA^ cells, hCEA–CAR T^WT^ cells frequently down-regulated surface IFNAR1 and displayed increased cleaved caspase-3 staining, consistent with enhanced susceptibility to IFN-driven dysfunction and apoptosis (Fig. 8, I and J). Last, longitudinal evaluation of serum alanine aminotransferase, aspartate aminotransferase (AST), and lactate dehydrogenase (LDH) revealed no evidence of increased systemic toxicity in either CAR T group (figs. S8L and S9E). Lung and colon tissues showed minimal CD3^+^ or GFP^+^ infiltration relative to metastatic lesions (figs. S8, I and J, and S10, A to C), supporting a favorable safety profile in this model, as previously reported (*49*).

**Fig. 8.**
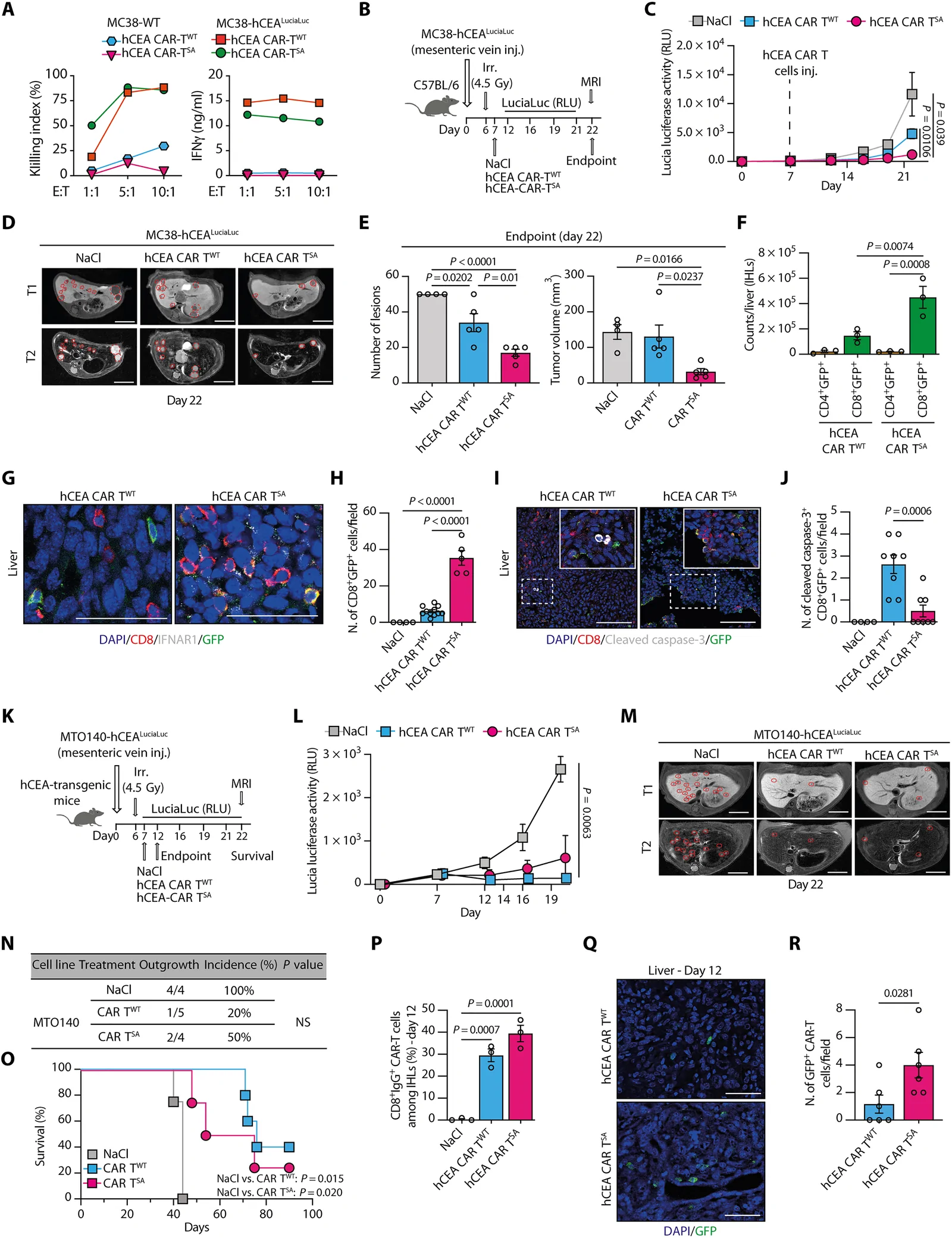
IFNAR1 stabilization enhances anti-CEA CAR T cell persistence and therapeutic efficacy in IFN-I^high^ CRC-LMs. (**A**) In vitro cytotoxicity and IFN-γ secretion by hCEA CAR T^WT^ and hCEA CAR T^SA^ against MC38-WT or MC38-hCEA^LuciaLuc^ target cells at the indicated effector-to-target (E:T) ratios. (**B**) Experimental scheme: MC38-hCEA^LuciaLuc^ cells (7 × 10^4^) were injected via the mesenteric vein on day 0; mice were lymphodepleted by sublethal irradiation (4.5 Gy, day 6) and received NaCl or hCEA CAR T^WT^ or hCEA CAR T^SA^ cells (1 × 10^7^ at day 7). Tumor burden was monitored by serum LuciaLuc activity and day 22 MRI. (**C**) Longitudinal serum LuciaLuc activity. (**D**) Representative T1- and T2-weighted MRI at day 22. Dashed lines delineate metastases. Scale bars, 5 mm. (**E**) Lesion number and total tumor volume. (**F**) Numbers of CD4^+^GFP^+^ and CD8^+^GFP^+^ CAR T cells among IHLs at day 22. (**G** and **H**) CD8, IFNAR1, GFP, and DAPI staining and CD8^+^GFP^+^ CAR T cells per field. Scale bar, 50 μm. (**I** and **J**) Cleaved caspase-3, CD8, GFP, and DAPI staining and apoptotic CAR T cells per field. Scale bar, 50 μm; insets show magnified regions. (**K**) MTO140-hCEA^LuciaLuc^ cells (1 × 10^5^) were injected into hCEA-transgenic mice and treated as in (B). (**L**) Longitudinal serum LuciaLuc activity. (**M**) Representative T1- and T2-weighted MRI at day 22. Dashed lines delineate metastases. Scale bar, 5 mm. (**N**) Tumor incidence at day 22. (**O**) Kaplan-Meier survival. (**P**) CD8^+^GFP^+^ CAR T cells among IHLs at day 12. (**Q** and **R**) GFP and DAPI staining and GFP^+^ CAR T cells per field. Scale bar, 50 μm. Each dot represents one mouse [(E), (F), and (P)] or field [(H), (J), and (R)]; bars show means ± SEM. Statistics: two-way ANOVA/Fisher’s LSD (C), one-way ANOVA/Tukey [(E), (F), (H), (J), and (P)], two-way ANOVA/Tukey (L), Fisher’s exact test (N), log-rank/Holm-Šídák (O), or two-sided Mann-Whitney test (R). *P* values are shown.

We next evaluated whether the benefit of IFNAR1 stabilization extends to an IFN-I^low^ metastatic context. MTO140 cells, which exhibit limited IFN-I activity (Fig. 4, B and C), were engineered to express hCEA^LuciaLuc^ and injected in the mesenteric vein into hCEA-transgenic mice, which recapitulate physiologic CEA expression and peripheral tolerance (*49*) (figs. S9, A to C, and S10A). Following lymphodepletion and randomization based on serum luciferase, mice received equal numbers of hCEA–CAR T^WT^ or hCEA–CAR T^SA^ cells (Fig. 8K). In this IFN-I^low^ setting, longitudinal serum Lucia luciferase measurements indicated that both CAR T cell products restrained early metastatic outgrowth relative to vehicle control (Fig. 8L). At day 22 after tumor injection, however, no consistent therapeutic advantage of IFNAR1 stabilization was observed: MRI-based quantification revealed comparable lesion numbers and total tumor volume between hCEA–CAR T^WT^ and hCEA–CAR T^SA^ groups (Fig. 8, M and N, and fig. S9D), and tumor-outgrowth incidence did not differ substantially among treatment groups (Fig. 8N). Both hCEA–CAR T^WT^ and hCEA–CAR T^SA^ treatments substantially prolonged overall survival compared with vehicle, whereas no substantial difference was detected between the two CAR T cell groups (Fig. 8O). Thus, IFNAR1 stabilization did not confer a durable therapeutic advantage over hCEA–CAR T^WT^ cells in this model.

To determine whether the absence of differential tumor control reflected similar intratumoral persistence, we examined early CAR T dynamics in this setting. Following lymphodepletion and transfer (Fig. 8K), analysis at day 12 showed that MTO140-hCEA^LuciaLuc^ lesions remained immunologically cold, with low CD8^+^ T cell accumulation. However, although quantification of tumor-infiltrating CAR T cells demonstrated a slightly increased percentage of hCEA–CAR T^SA^ compared with hCEA–CAR T^WT^ cells within the liver (Fig. 8, P to R, and fig. S9, F to H), IFNAR1 stabilization does not confer a consistent advantage in this IFN-I^low^ environment.

Together, these data support IFNAR1 destabilization as a context-dependent barrier to ACT efficacy in CRC-LMs. IFNAR1-stabilized CAR T cells showed improved intratumoral persistence and resistance to apoptosis in IFN-I^high^ lesions, resulting in enhanced antitumor activity without detectable off-tumor toxicity. By contrast, in IFN-I^low^ metastases, IFNAR1 stabilization preserved the CAR T phenotype but did not provide additional therapeutic benefit, consistent with the limited IFN-I activity in this setting (*44*, *45*).

## DISCUSSION

IFN-Is exert complex and context-dependent effects in cancer, with evidence supporting both antitumor and immunosuppressive roles. However, how tumor-intrinsic IFN-I production shapes the immune landscape of established CRC-LMs, and how this intersects with IFNAR1 regulation on effector CD8^+^ T cells, has remained unclear. The present study identifies tumor-intrinsic IFN-I dynamics in CRC-LMs and IFNAR1 down-regulation on effector CD8^+^ T cells as important regulators of immune dysfunction within metastatic niches. Across human synchronous CRC-LM specimens, we observed that IFNAR1 protein expression progressively declines from normal colon to primary CRC. In LMs, however, residual IFNAR1 expression is retained within discrete lymphocyte-rich perimetastatic invasive margin niches. Notably, IFNAR1 status within these regions stratified patients, with IFNAR1^high^ lesions associated with more favorable clinical outcomes compared with IFNAR1^low^ lesions, supporting a potential biomarker role in CRC-LM. These observations suggest that preservation of IFNAR1 expression within peritumoral immune niches is linked to improved immune control, aligning with the broader prognostic relevance of immune cell density and spatial organization in CRC (*34*, *38*, *50*). In parallel, using PDOs derived from metachronous CRC-LMs as well as established CRC cell lines, we defined IFN-I^high^ and IFN-I^low^ tumor states on the basis of tumor-intrinsic IFN-I ligand expression. Although IFN-I^high^ tumors have been reported to be enriched among MSI-H/dMMR cases (*51*, *52*), our data indicate that an IFN-I^high^ state can also arise independently of MSI-H/dMMR status, suggesting that tumor-intrinsic IFN-I production is not restricted to hypermutated disease. Furthermore, we observed heterogeneous expression of multiple IFN-I ligands (including several IFNA subtypes, IFNB1, IFNE, and IFNW1) across PDOs, supporting bona fide tumor-derived IFN-I production. While it remains to be formally demonstrated, this IFN-I^high^ state may be enriched or selected during metastatic progression and could contribute to the aggressive behavior of certain lesions. Mechanistically, dissemination-associated nuclear deformation and cellular stress may promote cytosolic DNA accumulation and engage cGAS-STING signaling, thereby inducing downstream IFN-I responses in metastatic tumors (*53*, *54*). Collectively, these findings define a functional IFN-I^high^ tumor state that intersects with IFNAR1 down-regulation within metastatic immune niches.

Our data extend existing paradigms of IFN-I function by delineating a context-dependent dichotomy between early protective and chronic suppressive IFN-I signaling within metastatic tumor niches (*11*, *55*–*57*). Previous preclinical studies have shown that perioperative IFN-α delivery can exert protective effects by reinforcing the liver endothelial barrier, limiting CRC cell extravasation, inducing intravascular tumor cell death, and promoting antitumor immunity before overt metastatic outgrowth (*20*, *21*). Consistently, IFN-α has been shown to enhance tumor-specific CD8^+^ T cell expansion (*58*) and increase responsiveness to PD-1/PD-L1 blockade when metastatic burden is low (*26*, *52*, *59*). By contrast, sustained or chronic IFN-I signaling has been associated with immunosuppression, adaptive resistance, and diminished responses to immune checkpoint inhibition (*24*, *30*, *60*, *61*). In keeping with this dichotomy, MC38- and MTO140-bearing livers displayed distinct expression patterns of individual SOCS family genes. The selective increase in *Socs1* in MC38-bearing livers is consistent with engagement of an IFN-I–responsive negative-feedback circuit (*62*), whereas the biological significance of the changes in *Socs5* and *Socs7* remains to be determined. Because these analyses were performed in bulk liver tissue, differences in the relative abundance of tumor, immune, stromal, and hepatic cell populations may also have contributed to the observed expression patterns. Nevertheless, our findings support a model in which, in established CRC-LMs, a tumor-intrinsic IFN-I^high^ state is associated with IFNAR1 internalization and down-regulation on effector CD8^+^ T cells, accompanied by impaired survival, reduced proliferative capacity, and acquisition of dysfunctional phenotypes.

Rather than broadly suppressing immunity, chronic IFN-I signaling appears to selectively compromise IFN-responsive and effector CD8^+^ T cell persistence within metastatic niches, contributing to their spatial exclusion from tumor lesions (*30*, *32*, *34*, *61*, *63*). These observations align with prior reports linking IFNAR1 degradation to stress-associated cues, including proinflammatory cytokines, endoplasmic reticulum stress, hypoxia, nutrient deprivation, and vascular endothelial growth factor signaling, which converge on p38α-dependent IFNAR1 ubiquitination and endocytic turnover (*34*, *64*).

Our functional studies show that pharmacologic inhibition of p38 and PKD/TGFβRI stabilizes IFNAR1 and preserves CD8^+^ T cell viability and effector function under sustained IFN-α exposure in vitro and in vivo, without exerting a measurable direct antiproliferative effect on MC38 cells. In this setting, IFNAR1 stabilization extends the window during which IFN-α therapy supports CD8^+^ T cell–mediated tumor control, including at stages when IFN-α monotherapy alone no longer confers measurable benefit in established metastatic disease and instead associates with induction of inhibitory programs such as T cell exhaustion and immune-checkpoint pathways (*22*–*24*, *26*, *30*, *52*, *59*–*61*).

Similarly, genetic stabilization of IFNAR1 (*Ifnar1*^S526A^) preserved effector CD8^+^ T cell function and enhanced tumor control in established IFN-I^high^ metastatic settings (MC38 model), further supporting a functional role for IFNAR1 regulation in shaping antitumor immunity. Notably, IFNAR1 stabilization did not impair IFN-α sensing, as OT-I^SA^ cells retained robust STAT1 responsiveness while preserving viability, proliferation, and effector function during sustained IFN-α exposure. Although the downstream mechanisms underlying this protection remain to be defined, these findings indicate that stabilizing IFNAR1 preserves T cell responsiveness to IFN-α without triggering the loss of T cell fitness observed in WT cells.

Collectively, these findings support the concept that IFNAR1-stabilizing approaches may recondition IFN-I–rich metastatic niches to better sustain CD8^+^ T cell persistence and function, thereby improving adoptive immunotherapy efficacy (*34*, *37*, *65*). In this context, IFNAR1 internalization emerges from our data not merely as a marker of chronic IFN-I exposure but as a mechanistically relevant modulator of CD8^+^ T cell survival and functional capacity. Loss of IFNAR1 on tumor-reactive cytotoxic T lymphocytes (CTLs) may reduce responsiveness to prosurvival cytokines such as IL-2, potentially weakening anti-apoptotic programs (*34*). This vulnerability could be amplified in the liver microenvironment, where local competition for cytokines by innate lymphoid populations has been reported to limit effector CD8^+^ T cell persistence (*66*). We further hypothesize that sustained IFNAR1 internalization under chronic IFN-I exposure may promote enhanced receptor turnover and lysosomal degradation, which could engage cellular stress pathways, including persistent autophagic flux or metabolic imbalance, thereby contributing to CD8^+^ T cell dysfunction. While not directly addressed in the present study, such mechanisms could intersect with nucleic acid sensing pathways, including cGAS-STING, which have been implicated in stress-associated inflammatory signaling in antiviral and tumor contexts (*67*–*69*).

Discriminating the IFN-I status of metastatic tumors becomes particularly relevant in the context of ACTs that rely on sustained CD8^+^ T cell fitness. Our data show that, in IFN-I^high^ MC38 liver lesions, both effector OT-I^WT^ CD8^+^ T cells and hCEA–CAR T^WT^ cells undergo IFNAR1 down-regulation, increased apoptosis, and limited persistence within tumor areas, mirroring the progressive exclusion of endogenous tumor-specific CD8^+^ T cells from metastatic lesions (*20*, *21*, *44*).

In contrast, IFNAR1-stabilized OT-I^SA^ CD8^+^ T cells and hCEA–CAR T^SA^ cells resist IFN-driven apoptosis, accumulate within metastatic foci, and achieve superior tumor control, with minimal off-tumor recruitment in lung and colon. Notably, this therapeutic advantage is evident in IFN-I^high^ models but attenuated in IFN-I^low^ settings established using MTO140 mouse-derived organoids. These findings suggest that IFNAR1 stabilization confers the greatest benefit in metastatic contexts in which chronic IFN-I signaling constitutes a dominant constraint on CD8^+^ T cell persistence. In IFN-I^low^ LMs, both hCEA–CAR T^WT^ and hCEA–CAR T^SA^ accumulated comparably at early time points following ACT, consistent with similar trafficking and initial tumor recruitment. However, over time, the absence of a selective advantage for IFNAR1 stabilization suggests that alternative immunoregulatory programs may predominate within IFN-I^low^ (cold) metastatic niches, including TGFβ-enriched pathways previously described in this model (*44*, *45*). Such mechanisms may limit T cell persistence independently of IFNAR1 regulation, thereby diminishing the relative impact of receptor stabilization in IFN-poor settings. More broadly, these findings underscore that the efficacy of ACT against established LMs is multifactorial and depends not only on the intrinsic fitness of the transferred T cells, but also on cell dose, tumor burden at the time of treatment, and the composition of the mTME, including local IFN-I availability and additional immunoregulatory pathways (*8*, *37*). Even when complete control was not achieved, subcurative OT-I^SA^ ACT markedly restrained residual metastatic growth, whereas continuous IFN-α did not provide additional tumor control despite a trend toward greater intralesional T cell accumulation.

From a translational perspective, these data support the integration of IFNAR1 protein expression [assessed by routine immunohistochemistry (IHC)] with transcriptional measures of tumor-intrinsic IFN-I activity as a potential strategy to stratify metastatic CRC (*70*). Patients with IFN-I^high^ lesions characterized by reduced IFNAR1 expression within peritumoral immune niches may be particularly suited to therapeutic approaches aimed at stabilizing IFNAR1, either pharmacologically in combination with IFN-α–based regimens (*20*, *21*), or through ACT approaches designed to resist IFNAR1 down-regulation (*9*). In this framework, chronic tumor-intrinsic IFN-I signaling is not uniformly detrimental but may represent a context-dependent vulnerability that can be therapeutically leveraged to enhance ACT efficacy. Future studies should prospectively validate IFNAR1-based patient stratification and define the safety, dosing, and clinical efficacy of IFNAR1-stabilizing strategies in metastatic CRC.

### Limitations and future directions

Our human analyses are retrospective and include treatment heterogeneity, which may confound associations between IFNAR1 expression and clinical outcome and warrant prospective validation. In particular, neoadjuvant chemotherapy was unevenly distributed across IFNAR1 groups, whereas IFNAR1 expression was measured only in surgical specimens obtained after treatment decisions had been made in accordance with the clinical guidelines and multidisciplinary assessment applicable at the time. The single posttreatment sampling time point therefore precludes determining whether chemotherapy directly affects IFNAR1 expression, and these clinical associations warrant prospective validation. Although detection of multiple IFN-I ligands in PDOs and murine CRC models supports the concept that ligand composition may shape signaling refractoriness, we did not directly define which endogenous IFN-I species (or combinations) are necessary and sufficient to drive IFNAR1 destabilization in vivo, an important area for future mechanistic dissection. Moreover, while our data highlight CD8^+^ T cells as a key compartment affected by IFNAR1 destabilization, we cannot exclude important contributions from other immune populations, effects on stromal compartments, or broader systemic effects of pharmacologic IFNAR1 stabilization to the observed antitumor response in IFN-I^high^ LMs (*22*–*26*). Last, whether other solid tumors that frequently metastasize to the liver engage similar IFNAR1-destabilizing programs, and thus exhibit comparable refractoriness to immunotherapy, remains to be determined and will require extension of these analyses to additional metastatic contexts.

## MATERIALS AND METHODS

### Human samples: Human synchronous cohort

A retrospective cohort of 105 patients diagnosed with CRC and synchronous LMs who underwent combined resection of the primary tumor and metastatic lesions at the Gastrointestinal and Hepatobiliary Surgery Unit, IRCCS San Raffaele Hospital (Milan, Italy), between January 2007 and June 2017, was reviewed. From this population, 44 patients with evaluable paired tissue specimens (primary CRC and matched LMs) were included in the study. The study was approved by the San Raffaele Hospital Ethics Committee in May 2018 (study code: “IFNAR1”) and conducted in accordance with the Declaration of Helsinki. For each patient, clinical and pathological data were extracted from medical records, including age, sex, primary tumor site, KRAS mutational status, histologic grade, pathological T and N stage, microsatellite instability status (MSI/MSS), receipt of neoadjuvant chemotherapy within 6 months before surgery, and oncological outcomes during follow-up for up to 5 years. The decision to proceed directly to surgery or to administer neoadjuvant chemotherapy was made according to contemporaneous clinical guidelines and multidisciplinary assessment. Neoadjuvant regimens included fluoropyrimidine- and oxaliplatin-based chemotherapy (FOLFOX, CAPOX/XELOX), irinotecan-based chemotherapy (FOLFIRI), or triplet chemotherapy (FOLFOXIRI), in some cases combined with targeted agents (bevacizumab, cetuximab, or panitumumab).

### ACC_ORG-LiMeT cohort of PDOs and human CRC cell lines

PDOs were established from 15 patients with histologically confirmed CRC**-**LMs treated at the Hepatobiliary Surgery Unit, IRCCS San Raffaele Hospital (Milan, Italy). Samples were collected after written informed consent in accordance with the Declaration of Helsinki. Study protocols were reviewed and approved by the San Raffaele Hospital Ethics Committee (study codes: “ACC_ORG” and “LiMet”) and authorized for the use of human specimens by the Institutional Biobank-Biological Resource Center (CRB-OSR) (BBMRI-ERIC ID: IT_1383758011993577). For each patient, clinical and pathological data were extracted from medical records, including age, sex, primary tumor site, KRAS mutational status, pathological grade and stage, microsatellite status (MSI/MSS), and receipt of neoadjuvant chemotherapy within 6 months before surgery. Liver metastasis specimens not required for diagnostic purposes were mechanically and enzymatically dissociated and cultured in Matrigel domes under human organoid-specific conditions. Briefly, surgical resections of CRC-LM were minced and digested in phosphate-buffered saline (PBS)/EDTA (5 mM) supplemented with 2× TrypLE (Gibco) and 500 U of recombinant deoxyribonuclease I (DNase I) (Roche) at 37°C for 60 min. Cells released into the solution were resuspended in growth factor-reduced Matrigel (Corning) and seeded in 24-well flat-bottom cell culture plates. After solidification, Matrigel domes were overlaid with Advanced Dulbecco’s modified Eagle’s medium (DMEM)/F12 (Gibco) supplemented with 1× B27 (Gibco), 1× N2 (Gibco), 1% penicillin-streptomycin (Euroclone), 1% glutamine (Gibco), and 1% bovine serum albumin (BSA) (Sigma-Aldrich), together with the following growth factors: epidermal growth factor (EGF) (50 ng/ml; PeproTech), Noggin (100 ng/ml; PeproTech), R-Spondin 1 (500 ng/ml; PeproTech), 10 nM gastrin (Sigma-Aldrich), fibroblast growth factor 10 (10 ng/ml; PeproTech), basic fibroblast growth factor (10 ng/ml; PeproTech), Wnt-3A (100 ng/ml; R&D Systems), 1 μM prostaglandin E2 (Tocris Bioscience), 10 μM Y-27632 (StemCell Technologies), 4 mM nicotinamide (Sigma-Aldrich), 0.5 μM A83-01 (Tocris Bioscience), and 5 μM SB202190 (Sigma-Aldrich). Medium was changed every 2 to 3 days. For in vivo experiments, PDOs were transduced to stably express luciferase using a third-generation lentiviral vector (LV) carrying the luciferase gene (pLenti PGK V5-LUC Neo, Addgene; gift from E. Campeau) and selected with G418 before use. Established human CRC cell lines, derived from metastatic lesions, COLO 205 [RRID:CVCL_0218 from American Type Culture Collection (ATCC), catalog no. CCL-222] and SW620 (RRID:CVCL_0547 from ATCC catalog no. CCL-227), were cultured in RPMI 1640 and DMEM, respectively, supplemented with 10% fetal bovine serum (FBS; Lonza). All organoids and cell lines were maintained at 37°C in a humidified atmosphere containing 5% CO_2_ and were routinely confirmed to be mycoplasma-free before experimental use.

### Murine tumor cell lines and tumor organoids

The murine MC38 colon adenocarcinoma cell line (MSI, H-2^b^, C57BL/6-derived; RRID:CVCL_B288) was maintained in DMEM (Gibco) supplemented with GlutaMAX, 10% FBS (Lonza), and 1% penicillin-streptomycin (Gibco). The MTO140 murine CRC organoid line (MSS, H-2^b^, C57BL/6-derived), generated from intestinal stem cells carrying conditional *Apc^fl/fl^*, *Kras^LSL-G12D^*, *Tgfbr2^fl/fl^*, and *Trp53^fl/fl^* alleles with *Lgr5^eGFP-creERT2^*-driven recombination (*44*), was cultured in Matrigel (Corning) drops overlaid with Advanced DMEM/F12 (2:1) containing B-27 supplement, recombinant human EGF (50 ng/ml), recombinant human Noggin (100 ng/ml), and 1 μM galunisertib. Cell number and viability were routinely monitored using a CytoSMART automated cell counter (Corning). Several derivative lines were generated for this study by lentiviral transduction. MC38-NGFR-OVA cells, expressing chicken OVA under the EF1α promoter, were generated using a LV provided by L. Naldini for antigen-specific assays; MC38-B18R-eGFP cells stably expressed the vaccinia virus–encoded IFN-binding decoy receptor B18R together with enhanced green fluorescent protein (eGFP) (VectorBuilder) (*47*). Both MC38-hCEA^LuciaLuc^ and MTO140-hCEA^LuciaLuc^ lines were generated by lentiviral transduction with the pHR-Sin-Bx-Em-IRES-hCEA-LuciaLuc LV, which expresses hCEA and a secreted Lucia luciferase reporter (LuciaLuc) for longitudinal, noninvasive in vivo bioluminescence imaging (*48*). All cell and organoid lines were expanded, cryopreserved, and authenticated by morphology, fluorescence profiling, and functional assays before use.

### In vivo mouse models

All preclinical in vivo experiments were conducted under protocols approved by the Institutional Animal Care and Use Committee (IACUC) at IRCCS San Raffaele Scientific Institute (IACUC nos. 1042, 1520, and 1532) and were authorized by the Italian Ministry of Health and local authorities in accordance with Italian law, and designed, conducted, and reported in accordance with institutional guidelines and ARRIVE recommendations, where applicable. Sample sizes for in vivo liver metastasis experiments were determined by a priori statistical power calculations using G*Power, based on previous experience with the same CRC liver metastasis models and comparable MRI-based quantification of hepatic lesions (*20*, *21*), and were reviewed and approved as part of the authorized IACUC protocols. Eight- to 10-week-old sex- and age-matched male C57BL/6 J (CD45.2; JAX Mice Strain; Charles River Laboratories; Strain Code 632), B6.SJL-Ptprc^a^Pepc^b^/BoyCrl mice expressing the Ptprc^a^ (CD45.1; Charles River Laboratories; Strain Code 494), and NOD-SCID (NOD.CB17-Prkdcscid/NCrCrl) (Charles River Laboratories; Strain Code 394) immunodeficient mice were purchased from Charles River Laboratories (Calco, Italy).

CD45.1^+^CD45.2^+^ F1 hybrid mice were obtained by crossing C57BL/6 J male (CD45.2) mice with CD45.1 female mice. *Ifnar1*αβ^−/−^ (B6.129S2-*Ifnar1*^*tm1Agt/Mm*^; RRID:MMRRC_032045-JAX; *Ifnar1*^KO^) mice were obtained from the Jackson Laboratory and backcrossed onto the C57BL/6 J background. C57BL/6-*Ifnar1*^*tm1.1Syfu*^/J (RRID:IMSR_035564-JAX; *Ifnar1*^SA^) crossed to C57BL/6-Tg (TcraTcrb; OT-I) mice referred to as OT-I^SA^ mice were provided by S. Y. Fuchs (University of Pennsylvania). Both OT-I and OT-I^SA^ mice express a transgenic TCR specific for the murine MHC-I allele H2k^b^, enabling recognition of the immunodominant OVA epitope (SIINFEKL; residues 257 to 264). C57BL/6 N-hCEA-Tg mice (*71*) were provided by H. Abken.

### Generation of experimental tumor models

Mesenteric vein injections were performed to induce LMs in most injected animals. MC38 cells were trypsinized and injected in PBS using a 29G needle, whereas MTO140 organoids were dissociated and processed into single-cell suspensions before injection.

For intrahepatic xenograft experiments, NOD-SCID immunodeficient mice were anesthetized and injected intrahepatically, directly beneath Glisson’s capsule, with 5 × 10^4^ PDO cells resuspended in a 1:1 mixture of Matrigel and PBS. To minimize postoperative infection risk, mice received enrofloxacin (300 mg/liter) in drinking water starting 1 day before surgery and continuing for 7 days thereafter. All experimental procedures followed previously described protocols (*20*, *21*).

### Peripheral blood analyses and biochemistry

At the indicated experimental time points, whole blood was collected from the retro-orbital plexus of anesthetized mice using Na-heparin–coated capillaries (Hirschmann Laborgeräte GmbH, Germany) and EDTA-coated vials (Microvette, Sarstedt, Germany). Hematological parameters were determined using an automated cell counter (ProCyte Dx, IDEXX Laboratories, USA). The extent of hepatocellular injury was assessed by measuring serum alanine aminotransferase, AST, and LDH activity at multiple time points after the treatment, as previously described (*72*).

### Recombinant mouse IFN-α therapy

Continuous intraperitoneal delivery of IFN-α (carrier-free, BioLegend, San Diego, CA, USA) was achieved by intraperitoneal implantation of mini-osmotic pumps (Alzet; Cupertino, CA, USA) capable of delivering 150 ng of IFN-α per day for either 14 days (0.25 μl/hour) or 28 days (0.11 μl/hour). NaCl-containing pumps were used as controls. To avoid MRI artifacts by metallic components, pumps were modified to be compatible with imaging, as previously reported. For in vitro experiments, IFN-α was used at a final concentration of 50 pg/ml (chronic exposure) or 200 pg/ml (acute exposure).

### Small-molecule kinase inhibitor treatment

Pharmacological stabilization of IFNAR1 was achieved using a combination of the p38 MAPK inhibitor LY2228820 (LY; Aurogene) and the PKD/TGFβ receptor I (ALK5; Aurogene) inhibitor SD-208. For in vitro experiments, both inhibitors were prepared in dimethyl sulfoxide at 10 mM and used at a final concentration of 1 μM. For in vivo treatment, SD-208 was resuspended in 1% methylcellulose in sterile NaCl at 7.8 mg/ml and administered by oral gavage at 3 mg/kg, whereas LY2228820 was resuspended in sterile NaCl at 30 mg/ml and administered at 1 mg/kg, as previously described (*34*). The drug combination or vehicle control (corresponding volumes of 1% methylcellulose) was administered by 300-μl oral gavage every 2 days starting 7 days after MC38 injection and continuing until day 28.

### Magnetic resonance imaging

MRI was performed at the Experimental Imaging Center, IRCCS San Raffaele Scientific Institute (Milan, Italy), using a 7.0-T preclinical scanner (Bruker BioSpec 70/30 USR; Paravision 6.0.1) equipped with a four-channel mouse body coil. Mice were anesthetized with isoflurane (3% for induction, 2% for maintenance in 1 liter/min O_2_) and maintained normothermic on a temperature-controlled bed with continuous monitoring of respiration and body temperature. Gadoxetic acid (Gd-EOB-DTPA; Primovist, Bayer) was administered intravenously at 0.05 μmol/g body weight immediately before imaging. T2-weighted (TurboRARE-T2; TR = 3394 ms, TE = 33 ms) and T1-weighted (RARE-T1; TR = 581 ms, TE = 8.6 ms) axial fat-saturated sequences were acquired during the hepatobiliary phase, as previously described (*20*, *21*). Images were analyzed independently by two radiologists, blinded to treatment group, using MIPAV [National Institutes of Health (NIH); version 5.3.4 or later]. LMs were identified as discrete focal T2-hyperintense and T1-hypointense lesions by a certified radiologist. The number of lesions corresponds to the total number of individual metastatic foci detected per mouse. Tumor volumes were calculated by manual region-of-interest (ROI) segmentation across consecutive slices, and total metastatic burden per mouse was determined as the sum of all individual lesion volumes.

### Immunohistochemistry

Formalin-fixed, paraffin-embedded (FFPE) sections (3 μm) from mouse and human samples were processed using the Bond RX Automated Stainer (Leica Microsystems GmbH, Wetzlar, Germany). Primary antibodies, suppliers, catalog numbers, RRIDs, antigen-retrieval conditions, and working dilutions are listed in table S3. After deparaffinization, antigen retrieval was performed using Epitope Retrieval Solution 1 (citrate buffer, pH 6.0) or Epitope Retrieval Solution 2 (EDTA buffer, pH 9.0) according to antibody specifications. Detection was performed using the Bond Polymer Refine Detection Kit (DS9800, Leica Microsystems) with DAB chromogen development for 10 min, followed by hematoxylin counterstaining for 5 min. IFNAR1 IHC on human samples was additionally performed by the Pathology Department of IRCCS San Raffaele Hospital using the validated protocol “Jolly Umap Rb Tit 40+20 Univer (03/05/2017)” on a Ventana Discovery ULTRA Staining Module (Ventana Medical Systems Inc.). Sections were included in the analysis only if tissue morphology, staining quality, and counterstaining were adequate for digital quantification. Four colon sections and two liver sections failed quality control and were excluded from analysis because of insufficient tissue area. In the ACC_ORG-LiMeT cohort, IFNAR1 IHC was performed on available FFPE liver metastasis tissue. Cases for which histological material was not available were annotated as n.a. and were not included in IHC-based analyses. Quality control criteria were applied before quantitative analysis. All images were acquired and managed using a specific macro for each marker in the Aperio system and analyzed with eSlide Manager and ImageScope software (Leica Biosystems; v12.4.6.5003). A certified pathologist, blinded to treatment group, identified ROIs corresponding to normal colonic mucosa and tumor areas, and in the liver, uninvolved parenchyma, perimetastatic margins, and intrametastatic regions. Quantification within each ROI was performed using a customized color-deconvolution algorithm in ImageScope (Leica Biosystems), following the manufacturer’s guidelines. The total analyzed area included 145 mm^2^ of normal colon, 1372 mm^2^ of CRC tissue, 809 mm^2^ of uninvolved parenchyma, 183 mm^2^ of perimetastatic margin, and 580 mm^2^ of intrametastatic area. Representative images were selected from these ROIs and exported as ImageScope snapshots for figure preparation.

### Multiplex immunofluorescence staining and imaging

Multiplex immunofluorescence staining was performed using the Opal workflow, which allows simultaneous detection of up to six tissue biomarkers plus a nuclear counterstain within a single image. A four-antibody panel was developed using the commercially available Opal 6-Plex Detection Kit (NEL811001KT, Akoya Biosciences). The panel included anti-CD4 (Leica, ready-to-use) paired with Opal 480, anti-CD8 (Leica, ready-to-use) paired with Opal 520, anti-MUM1/IRF4 (Cell Marque, ready-to-use) paired with Opal 620, and anti-IFNAR1 (Sigma-Aldrich) paired with Opal 690. Spectral 4′,6-diamidino-2-phenylindole (DAPI) (Akoya Biosciences) was used as nuclear counterstain (table S3). Briefly, FFPE tissue slides underwent three deparaffinization cycles of 8 min each, followed by antigen retrieval using BOND Epitope Retrieval Solution 2 (Leica Biosystems) for 40 min at 97°C. Slides were then sequentially incubated with each primary antibody for 16 min at 36°C, followed by incubation with Opal anti-MS+RT HRP reagent for 20 min and the corresponding Opal fluorophore for 8 min. Spectral DAPI was applied for 5 min, and slides were mounted with Dako mounting medium, according to the manufacturer’s instructions. All staining steps, except coverslip mounting, were performed on the Akoya PhenoImager HT platform. Images were acquired using the PhenoImager HT 2.0 system. Spectral unmixing was performed using the live unmixing preview and the built-in synthetic spectral library. Unmixed multispectral images were visualized with Phenochart 2.0.0 software (Akoya Biosciences) and exported as fluorescent images or as QPTIFF files for downstream spatial analysis in QuPath.

### Computational processing and spatial analysis of multiplex immunofluorescence data

Unmixed multispectral QPTIFF files generated from the PhenoImager HT 2.0 workflow were analyzed in QuPath (v0.5.1) (*73*). Under the supervision of a pathologist, three ROIs were manually annotated on each tissue section: uninvolved parenchyma (ROI1), perimetastatic invasive margin (ROI2), and intrametastatic regions (ROI3). Cell segmentation was performed using StarDist (*74*), with the qupath-extension-stardist-0.5.0 plugin and the pretrained model stardist_cell_seg_model.pb. To ensure comparability across regions, approximately 5000 cells were segmented per ROI and expressed as the percentage of positive cells among total segmented DAPI-positive cells. Cell phenotyping was performed using supervised machine learning based on the expression of the marker panel. After manual annotation of representative cells (20 to 50 cells per phenotype), a two-step classification strategy was implemented. First, a classifier was trained to identify T cells (CD8^+^ or CD4^+^) on the basis of mutually exclusive CD8 and CD4 expression. Subsequently, independent classifiers were trained to identify MUM1^+^ and IFNAR1^+^ cells. This approach generated three independent object classifiers: CD8^+^/CD4^+^/DAPI^+^, MUM1^+^/DAPI^+^, and IFNAR1^+^/DAPI^+^. Each classifier was trained using the artificial neural network (ANN_MLP) algorithm, with “cells” set as the detection parameter and cell mean intensity measurements used to define marker-specific thresholds. Classifiers were applied sequentially across all ROIs. Quantitative data from each ROI were then exported from QuPath for downstream visualization and statistical analysis.

### Immunofluorescence and confocal microscopy

Livers were perfused with PBS, harvested and fixed with either zinc formalin (Bio-Optica) for 4 hours and embedded in paraffin or fixed for 2 hours in antigen fix solution (Diapath), equilibrated in 30% sucrose in PBS 2 hours at 4°C before embedding in OCT (Bio-Optica) for quick freezing at −80°C. Superfrost Plus slides (Thermo Fisher Scientific) were used for paraffinized or cryosections. After deparaffinization, samples were permeabilized and blocked with PBS containing 5% FBS and 0.1% Triton X-100 (Sigma-Aldrich) for 30 min at room temperature and subsequently incubated with 10% of donkey serum (DS; Sigma-Aldrich) in PBS for 30 to 60 min at room temperature. All staining procedures using the primary and secondary antibodies listed in table S3 were performed in staining buffer (PBS containing 1.5% DS, 0.2% Triton X-100, and 1% BSA). Confocal images were acquired using a Leica SP8 confocal system (Leica Microsystems). Z-stacks (15 to 20 μm) were projected in two dimensions and processed using Fiji image processing software (NIH). Fluorescence intensity in each ROI was quantified using the ROI Manager in Fiji software (Analyze→Measure), recording the mean value. Background fluorescence was measured in adjacent signal-free area and subtracted from each measurement. For each condition, five randomly selected fields were analyzed. Cell counting was performed using the Cell Counter plugin.

### Isolation of ICs

ICs were isolated from colons of WT C57BL/6 mice using a chelating buffer assay (*75*). Briefly, intestinal tissues were washed, and the underlying muscle layers were removed using surgical scissors. The intestinal segment was gently flushed with cold collection medium using a 10-ml syringe fitted with a needle. The tissue was opened longitudinally and cleaned to remove mucus. It was then cut into 5-mm fragments and incubated in cold chelating buffer on ice for 30 min. The supernatant containing crypts was collected into 50-ml Falcon tubes precoated with BSA, filtered through a 100-μm cell strainer, and centrifuged at 400*g* for 5 min at 4°C to separate crypts from single cells. Crypt purity was assessed by flow cytometry using EpCAM- and CD45-specific antibodies.

### IFN-α enzyme-linked immunosorbent assay

Circulating pan–IFN-α levels were quantified in plasma collected from NaCl-treated controls or IFN-α–treated mice at the indicated time points using the VeriKine-HS Mouse IFN-α ELISA Kit (PBL, table S4) according to the manufacturer’s instructions. IFN-α levels in confluent MC38- or MTO140-conditioned media were measured after overnight lyophilization at −20°C and reconstitution in ^1^/_10_ of the original volume, yielding a 10-fold concentration. Pan–IFN-α titers were determined by plotting blank-subtracted optical density (OD) values against a four-parameter logistic standard curve generated in Prism v10.6.1 (GraphPad). The assay detection range was 2.38 to 152 pg/ml.

### B16-Blue IFN-α/β reporter bioassay (IFN-I bioactivity)

Functional IFN-I activity was quantified in the same 10-fold concentrated MC38- or MTO140-conditioned media described above. Native conditioned media concentrates were used to stimulate B16-Blue IFN-α/β reporter cells (InvivoGen) in exponential growth phase. B16-Blue cells are stably transfected with a secreted embryonic alkaline phosphatase (SEAP) reporter under the control of the IFN-α/β-inducible ISG54 promoter. For the assay, 20 μl of each sample or control was dispensed into flat-bottom 96-well plates, followed by 180 μl of B16-Blue cell suspension (approximately 75,000 cells per well). Plates were incubated overnight at 37°C with 5% CO_2_. The following day, 20 μl of each B16-Blue supernatant was transferred to black optical 96-well plates (Thermo Fisher Scientific) containing 180 μl of QUANTI-Blue detection reagent (InvivoGen). Plates were incubated at 37°C for 3 hours, and SEAP activity was measured using a Victor spectrophotometer at 620 to 655 nm (PerkinElmer Life and Analytical Sciences). Background absorbance from cell-free wells containing QUANTI-Blue reagent was subtracted from each measurement for normalization. IFN-I bioactivity was calculated by interpolating SEAP values against an IFN-α standard curve.

### RNA extraction and quantitative real-time PCR gene expression analyses

Total RNA was isolated from liver homogenates, CRC cell lines, and murine and human organoid pellets using the ReliaPrep RNA Tissue or Cell Miniprep System (Promega) and subsequently treated with TURBO DNase (Thermo Fisher Scientific) according to the manufacturer’s recommendations. RNA was quantified using a NanoDrop spectrophotometer (Thermo Fisher Scientific), and integrity was assessed using a 4200 TapeStation System (Agilent Technologies, Santa Clara, CA, USA). Only samples with an RNA integrity number greater than 8 were included in subsequent analyses. cDNA was synthesized using SuperScript IV VILO Master Mix (Thermo Fisher Scientific) according to the manufacturer’s protocol, as previously described (*72*). For quantification of human and murine IFN-I subtypes, custom prespotted TaqMan Array Cards (Applied Biosystems) were used. Briefly, 100 to 200 ng of cDNA were preamplified using a custom primer pool specific for the genes included in the assay with TaqMan PreAmp Master Mix (Applied Biosystems, table S5), following the manufacturer’s protocol. Subsequently, 100 μl of TaqMan Fast Advanced Master Mix and diluted preamplified cDNA (1:1) were loaded into each reservoir of the array card and run on a QuantStudio 12 K Flex Fast Real-Time PCR System (Applied Biosystems) in fast-cycling mode, according to the TaqMan Array Cards manual (ref. 4400263). Raw data (.eds files) were imported into Thermo Fisher Connect software for preliminary analysis, with a defined control group for each experiment. The mean equivalent Cq value was used instead of Ct to correct for technical triplicate bias. The TBP housekeeping gene was used for ∆Cq normalization. Relative gene expression was calculated using the 2^−ΔΔCt^ method, where ΔCq = Cq(GOI) − Cq(TBP) and ΔΔCq = ΔCq(sample) − mean ΔCq(control), as previously described (*72*). For validation of selected individual genes, SYBR Green real-time PCR was performed using gene-specific primers described in table S5, with GAPDH used as housekeeping gene. Absolute quantification of IFN-I subtypes was performed using the QIAcuity Digital PCR system (QIAGEN, Hilden, Germany) according to the manufacturer’s protocol. The optimal cDNA input was determined in preliminary dilution experiments and set at 50 ng per reaction. A 12-μl reaction mixture containing 20 × TaqMan gene expression assay, 4 × QIAcuity Probe PCR Master Mix (Qiagen), and the appropriate amount of cDNA was prepared and loaded into a QIAcuity Nanoplate 8.5 K 96-well plate (Qiagen). Plates were processed in a QIAcuity One instrument (Qiagen) through sample partitioning, 40 amplification cycles, and FAM fluorescence acquisition. The number of positive partitions per target was determined using the QIAcuity software suite (Qiagen) and converted to copies per reaction, normalized to cDNA input. In accordance with manufacturer recommendations, reactions with more than three positive partitions were considered reliable.

### RNA sequencing

Total RNA isolated from sham controls and from MC38 and MTO140 metastatic livers that passed quality control was processed for bulk RNA sequencing (Macrogen). Strand-specific mRNA libraries were prepared using the TruSeq Stranded mRNA LT Sample Prep Kit according to the TruSeq Stranded mRNA Sample Preparation Guide (Part no. 15031047 Rev. E) and sequenced on an Illumina platform with paired-end 101-bp reads. Across 11 samples, sequencing generated an average of approximately 20 million reads per sample, with GC content ranging from ∼47.6 to 49.7% and Q30 values of ∼95.1 to 96.0%. Raw reads were trimmed using Trimmomatic (v0.32) to remove adapter sequences and low-quality bases. Filtered reads were aligned to the murine reference genome (GRCm39) using STAR (v2.5.3a). Gene-level counts were generated using featureCounts based on Gencode basic annotations (version M33). Sequencing and alignment quality were assessed using FastQC, RSeQC, and MultiQC. Expressed genes were defined as those with at least 1 count per million in a minimum number of samples, depending on the size of the comparison groups. Genes not meeting these criteria were excluded from downstream analyses. Gene count matrices were imported into the R environment (v4.0.3) for differential expression analysis using the DESeq2 Bioconductor package (v1.30.1) (*76*). Principal components analysis was carried out using the prcomp R package: Data were preliminary centered and scaled, to get zero-mean and unit variance on each gene. Differentially expressed genes (DEGs) were identified as those with a FDR < 0.01 and an absolute log_2_FC > 1. Functional enrichment analysis was conducted using the enrichR R package (v3.0) (*77*) based on DEGs defined by FDR < 0.01. Enriched pathways were grouped and summarized according to biological function, and statistical significance was assessed using the hypergeometric test implemented in the hypeR R package (v1.8.0). Preranked Gene set enrichment analysis (*78*) was performed for each differential expression comparison using all expressed genes ranked by log_2_ fold change. Gene sets were obtained from the Canonical Pathways, Hallmark, and Gene Ontology collections in the MSigDB database. On the basis of a previously published human IFN-I signature, we analyzed the corresponding murine ortholog signature consisting of 27 representative genes (*26*).

### Isolation of splenocytes and generation of effector OT-I T cells

Spleens were harvested from mice and placed onto a 70-μm cell strainer positioned over a petri dish containing 10 ml of plain RPMI 1640. Tissue was mechanically dissociated using a syringe plunger to obtain a single-cell suspension, which was washed three times with RPMI 1640. The cell suspension was centrifuged at 400*g* for 5 min at room temperature. The pellet was resuspended, treated with ACK lysis buffer to remove red blood cells, and neutralized with RPMI 1640. Single-cell splenocyte suspensions were plated in 48-well plates (one spleen per well) and stimulated overnight with SIINFEKL peptide (1 μg/ml; OVA₍_257_-_264_₎, ProImmune) in T cell culture medium (TCM; RPMI 1640 supplemented with 10% FBS, 2 mM l-glutamine, 50 μM 2-mercaptoethanol, 10 mM Hepes, 100 μM nonessential amino acids, and penicillin-streptomycin). Cells were then washed, diluted 1:4, and incubated at 37°C with 5% CO_2_ to promote effector T cell generation and expansion. After 2 days, cultures were diluted 1:2 in fresh medium supplemented with 2.5% IL-2–containing EL-4 supernatant and replaced every 2 days. After 7 to 8 days, effector OT-I T cells were used for the indicated experiments.

### Acute and chronic IFN-α stimulation of effector OT-I T cells

Effector OT-I T cells generated as described above were used on days 6 to 8 for acute or chronic IFN-α stimulation assays. For acute stimulation, effector OT-I cells were restimulated with SIINFEKL peptide (1 μg/ml) for 1 hour and then treated with recombinant IFN-α (200 pg/ml) or vehicle for 2 hours before analysis of IFNAR1 surface expression and STAT1 phosphorylation. IFNAR1 surface expression was assessed by flow cytometry, whereas pSTAT1(Tyr701) was analyzed by immunofluorescence. For chronic stimulation, effector OT-I cells were restimulated with SIINFEKL peptide (1 μg/ml) for 4 hours and then cultured with recombinant IFN-α (50 pg/ml) or vehicle for 2 or 4 days. Following chronic IFN-α exposure, cells were analyzed by flow cytometry to assess IFNAR1 surface expression, Ki67, PD-1, TIM-3, and memory/effector subset distribution. Spleen cells prepared from *Ifnar1*αβ^−/−^ knockout animals were used as background controls and to set negative gates. Dead cells were excluded using Live/Dead Fixable Near-IR or Green viability dyes, and all flow cytometry analyses were performed after gating on live CD8^+^ OT-I cells. Marker frequencies were therefore quantified within the viable CD8^+^ OT-I T cell population. Apoptosis was assessed independently by cleaved caspase-3 immunofluorescence staining (table S3).

### Coculture assay

Effector OT-I^WT^ and OT-I^SA^ cells (1 × 10^5^ cells per well) were cocultured with MC38^OVA^ cells (1 × 10^5^ cells per well) at a 1:1 effector-to-target (E:T) ratio in 48-well plates using a 1:1 mixture of TCM and DMEM for 4 days. At the end of the culture period, T cells and tumor cells were harvested together. The two populations were discriminated by staining with an anti-CD8 antibody for flow cytometric analysis. Cell viability was assessed using the Live/Dead Fixable Green Dead Cell Stain Kit (table S4).

### CAR retroviral vector construction

A retroviral MSCV-based vector was engineered to coexpress a CEA-specific chimeric antigen receptor (CEA-CAR), provided by H. Abken, together with a GFP reporter. The CEA-CAR cassette comprised an N-terminal signal peptide, a CEA-binding scFv, hinge and transmembrane domains, and intracellular signaling modules including costimulatory domain(s) and CD3ζ, as previously described (*49*, *79*). The CAR coding sequence was cloned into the MSCV retroviral backbone, followed by GFP downstream of an internal ribosome entry site (IRES), generating the RV_MSCV_CEA.CAR-IRES_GFP construct, which enables coordinated expression of CEA-CAR and GFP from a single transcript. Plasmid integrity was confirmed by restriction enzyme analysis and Sanger sequencing across cloning junctions and the full CAR open reading frame before retroviral packaging and transduction. The hCEA-CAR retrovirus was produced in Phoenix-ECO packaging cells as previously described (*79*).

### CAR T cell preparation

Splenic T cells from C57BL/6 J and C57BL/6-*Ifnar1*^*tm1.1Syfu*^/J (IFNAR1^SA^) mice were isolated under sterile conditions by mechanical dissociation, ACK-mediated red blood cell lysis, and negative selection using the Pan T Cell Isolation Kit-II and LS columns (Miltenyi), following the manufacturer’s instructions. Purified T cells were activated with Dynabeads Mouse T-Activator CD3/CD28 (Gibco) at a 3:1 bead-to-cell ratio. A total of 5 × 10^5^ cells per well were seeded in 48-well plates in complete RPMI 1640 supplemented with recombinant human IL-2 (50 U/ml) and cultured at 37°C. After 48 hours, activated T cells were transduced by spinoculation with concentrated retrovirus [multiplicity of infection (MOI) = 6] in the presence of polybrene (8 μg/ml) (800*g* for 2 hours at 30°C), while nontransduced controls received polybrene only. Following centrifugation, wells were replenished with prewarmed complete RPMI 1640 containing IL-2 (50 U/ml) and returned to 37°C incubation. A second transduction at the same MOI was performed 24 hours later. From day 3 after activation, CAR T cells were expanded in complete RPMI 1640 supplemented with murine IL-7 and IL-15 (10 ng/ml). Transduction efficiency was assessed by flow cytometry 5 days after the initial transduction. CAR T cells were used for in vitro assays or adoptive transfer between days 7 and 9 of culture. Before in vivo transfer, magnetic beads were removed using a magnetic separator, and cells were washed three times in PBS before injection. All procedures involving retrovirus and transduced cells were performed in Class II biological safety cabinets in accordance with institutional biosafety regulations.

### CAR T cell in vitro cytotoxic activity

For cytotoxicity assay, MC38-WT and MC38-hCEA^LuciaLuc^ target cells were seeded in 96-well U-bottom plates. Effector hCEA–CAR T^WT^ and hCEA–CAR T^SA^ cells were added at E:T ratios of 1:1, 5:1, or 10:1. After 48 hours, supernatants were collected and stored at −20°C for subsequent IFN-γ enzyme-linked immunosorbent assay (ELISA) analysis. Cells were harvested and counted using the trypan blue exclusion assay. Cells were then stained with anti-CD3, anti-CEA, and DAPI and analyzed by flow cytometry. Target cells were identified on the basis of forward scatter (FSC) and CEA expression. The Killing index was calculated as follows: 1 − (absolute number of residual target cells in presence of transduced T cells/absolute number of residual target cells in presence of untransduced T cells).

### Adoptive cell transfer

OT-I T cells were isolated and expanded from donor spleens as described above. A total of 1 × 10^7^ OT-I cells were resuspended in 200 μl of PBS and administered intravenously via tail-vein injection into CD45.1^+^CD45.2^+^ recipient mice. Circulating CD8^+^ cells were distinguished from endogenous populations using anti-CD45.1-PERCP/Cy5.5 and anti-CD45.2-PB antibodies. For CAR T cell transfer, transduction efficiency was assessed on the basis of the percentage of GFP^+^ cells. Accordingly, cell numbers were adjusted to ensure transfer of 1 × 10^7^ GFP^+^ CAR T cells per mouse. Cells were resuspended in 100 μl of PBS and administered via intravenous tail-vein injection into lymphodepleted mice (4.5-Gy x-ray irradiation; RS 2000 irradiator, Rad Source Technologies Inc.).

### Isolation of PBMCs and evaluation of circulating OT-I T and CAR T cells

Peripheral blood mononuclear cells (PBMCs) were isolated from blood collected from mice under isoflurane anesthesia (5% for induction and 2% for maintenance in 2 liter/min O_2_) by retro-orbital sampling using heparinized capillaries. Blood was collected into EDTA-coated tubes, maintained at 4°C, and used to quantify circulating OT-I and CAR T cells. For each blood collection (day 1 postadoptive transfer for OT-I cells and day 9 for CAR T cell quantification), 150 to 200 μl of blood was diluted to 500 μl with PBS containing EDTA and centrifuged (∼500*g* for 10 min at 4°C). Red blood cells were lysed by resuspending the cell pellet in 3 ml of ACK lysing buffer (1×; Lonza) for 2 to 5 min at room temperature. Samples were then washed with PBS, centrifuged at ∼500*g* for 5 min, and the final pellet was resuspended in 200 to 500 μl of fluorescence-activated cell sorting (FACS) buffer. Cells were transferred to 96-well U-bottom plates and stained with specific antibodies for flow cytometry analysis.

### Isolation of IHLs

Animals were euthanized by cervical dislocation under anesthesia. The abdomen was opened, and the liver was perfused retrogradely with 10 ml of PBS (Corning) to remove circulating lymphocytes and blood cells. The liver was excised, cut into small fragments, and mechanically dissociated using a syringe plunger. The resulting cell suspension was filtered through a 70-μm cell strainer, washed with RPMI 1640, and centrifuged at 500*g* for 5 min. The pellet was resuspended in digestion medium consisting of RPMI 1640 supplemented with type IV collagenase (200 U/ml; Sigma-Aldrich) and type I DNase (100 U/ml; Sigma-Aldrich) and incubated at 37°C for 40 min with vortexing every 10 min. Following digestion, samples were briefly vortexed and centrifuged at 50*g* for 3 min to allow precipitation of connective tissue. The supernatant containing IHLs was collected and centrifuged at 500*g* for 5 min. The resulting pellet was resuspended in 4 ml of a 36% Percoll solution, prepared by mixing 2.4 ml of RPMI 1640, 0.16 ml of 10× PBS, and 1.44 ml of Percoll (Sigma-Aldrich) per sample, and centrifuged at 800*g* for 20 min without brake. The leukocyte pellet was collected and treated with ACK lysis buffer (Lonza) for 2 min, followed by centrifugation at 500*g* for 5 min. Cells were resuspended in RPMI 1640 and counted using a CytoSMART automated cell counter or a Bürker counting chamber following dead-cell exclusion with Trypan Blue (Sigma-Aldrich). The resulting single-cell suspensions were subsequently processed for flow cytometry using the antibodies listed in table S6.

### Flow cytometry

Samples were acquired on BD FACSCanto II, BD FACSymphony A5 SORP instruments (BD Biosciences), and data were analyzed using FlowJo v10 (BD). Single-cell suspensions were stained with Live/Dead Fixable Near-IR or Green dyes (Invitrogen) or DAPI (15 min at room temperature) to assess viability. Cells were blocked for 15 to 20 min in FACS buffer (PBS supplemented with 2% FBS) containing anti-CD16/CD32 Fc block, followed by staining with fluorochrome-conjugated antibodies (table S6) for 20 min at 4°C. For intracellular staining (Ki67, pSTAT1, IFN-γ), cells were fixed and permeabilized using Foxp3/Transcription Factor or Cytofix/Cytoperm buffer sets according to the manufacturers’ protocols. For IFN-γ detection, cells were restimulated in the presence of brefeldin A (1:1000) for 4 hours before staining. Where indicated, cells were fixed in 2% paraformaldehyde (PFA) for 10 min at 4°C before acquisition. Doublets and dead cells were excluded on the basis of FSC-A versus FSC-H discrimination and viability gating. For high-dimensional analysis of transferred OT-I cells, CD45.2^+^CD8^+^ events were pregated, QC using FlowAI, randomly down-sampled, concatenated, and subjected to t-SNE and FlowSOM clustering within FlowJo. Clusters were annotated according to CD25, CD44, CD62L, IFN-γ, TNFα, PD-1, and TIM-3 expression profiles. Cytek Aurora (Cytek Biosciences) was used to characterize immune populations in the tumor microenvironment (TME) of MC38 tumor-bearing mice. Flow cytometry data were initially cleaned in R using the PeacoQC package (v1.12.0) to remove low-quality events. Cleaned FCS files were imported into FlowJo (v10.10.0) for spillover correction, dead cell and doublet exclusion, and random down-sampling to 8000 CD45^+^ events. Processed data were exported for downstream analysis in R (v4.3.1), where sample batches were loaded using read.flowSet (flowCore, v2.14.2). Logicle transformation and marker-wise normalization (gaussNorm, flowStats, v4.14.1) were applied to reduce technical variability across batches. Batch-corrected samples were merged into a SingleCellExperiment object using prepData (CATALYST, v1.26.1). Dimensionality reduction was performed using UMAP, and unsupervised clustering and meta-clustering were carried out using FlowSOM (v2.10.0) and ConsensusClusterPlus (v1.66.0), following established workflows (*80*).

### Statistical analysis

Human synchronous cohort. Differences between two paired groups were assessed using a two-sided nonparametric Wilcoxon matched-pairs signed-rank test. Differences among three paired groups were evaluated using a two-sided nonparametric Friedman test for matched measures, followed by Dunn’s multiple-comparisons test. For survival analyses, patients with evaluable perimetastatic IFNAR1 expression were dichotomized at the cohort mean, generating IFNAR1^low^ and IFNAR1^high^ groups. Overall survival was administratively censored at 60 months, and patients without evaluable survival follow-up were excluded. Kaplan-Meier survival curves were compared using a two-sided log-rank (Mantel-Cox) test. Multivariable survival analysis was performed using an exploratory Cox proportional hazards regression model adjusted for age, sex, histologic grade, pT, and pN. Histologic grade, pT, and pN were entered as ordinal covariates. IFNAR1^high^ was used as the reference group; thus, hazard ratios refer to IFNAR1^low^ versus IFNAR1 ^High^ patients. Tied event times were handled using the exact method, and patients with missing data for one or more model covariates were excluded by complete-case analysis. Hazard ratios are reported with 95% profile-likelihood confidence intervals, and *P* values for individual model coefficients were determined using likelihood-ratio tests. The association between IFNAR1 status (High versus Low) and prior neoadjuvant chemotherapy exposure was evaluated using a two-sided Fisher’s exact test, yielding the odds ratio and corresponding *P* value.

For in vitro and in vivo experiments, statistical significance was assessed using a two-sided nonparametric Mann-Whitney test for unpaired two-group comparisons or ordinary one-way or two-way analysis of variance (ANOVA), or mixed models, as appropriate. ANOVA and mixed models were followed by the multiple-comparisons procedure specified in the corresponding figure legend. When data did not meet normality assumptions, the Kruskal-Wallis test, followed by Dunn’s multiple-comparisons test, was applied. Contingency tables were tested using a two-sided Fisher’s exact test. Survival experiments were evaluated using the log-rank (Mantel-Cox) test. When more than two survival curves were compared, pairwise comparisons were adjusted using the Holm-Šídák method, as indicated. For comparisons of immune-cell populations between sham-treated and MC38-bearing livers, multiple two-sided Mann-Whitney tests were performed, one for each immune-cell population. Multiple comparisons were controlled using the two-stage step-up FDR procedure of Benjamini, Krieger, and Yekutieli, with Q set at 5%. FDR-adjusted *P* values are reported. All statistical analyses were performed using GraphPad Prism 11.0.2 (GraphPad Software) and are specified in the corresponding figure legends. All tests were two sided unless otherwise stated. *P* values < 0.05 were considered statistically significant. Unless otherwise indicated, differences were considered not significant (NS).
