# Supplementary material for: IFNAR1 stabilization sustains CD8+ T cell persistence in IFN-high colorectal cancer liver metastases
Source: Sci Adv. 2026 Nov 4;12(45):eaei0181. doi: 10.1126/sciadv.aei0181 (PMC13733802; doi:10.1126/sciadv.aei0181)
Supplement: Supplementary file 1 [file sciadv.aei0181_sm.pdf]

Science Advances
AAAS

Supplementary Materials for

IFNAR1 stabilization sustains $CD8^+$ T cell persistence in IFN-high colorectal cancer liver metastases

Andrea Castiglioni *et al.*

Corresponding author: Giovanni Sitia, sitia.giovanni@hsr.it

Sci. Adv. **12**, eaei0181 (2026)
DOI: 10.1126/sciadv.aei0181

The PDF file includes:

Figs. S1 to S10
Tables S1 to S6
Legends for data S1 to S8

Other Supplementary Material for this manuscript includes the following:

Data S1 to S8

A

	CRC-LM1	CRC-LM2	CRC-LM3	CRC-LM4	CRC-LM5	CRC-LM6	CRC-LM7	CRC-LM8	CRC-LM9	CRC-LM10	CRC-LM11	CRC-LM12	CRC-LM13	CRC-LM14	CRC-LM15	CRC-LM16	CRC-LM17	CRC-LM18	CRC-LM19	CRC-LM23	CRC-LM24	CRC-LM25
Age	81	51	83	75	57	79	81	70	46	49	78	67	77	81	81	90	78	84	69	81	65	63
Sex	f	f	f	m	f	m	f	f	f	f	m	m	m	m	f	m	m	f	m	f	m	m
Tumor Site	Total	Right	Right	Right	Rectum	Right	Left	Right	Left	Left	Left	Right	Right	Left	Right	Right	Left	Left	Left	Left	Left	Left
KRAS	Mut	Mut	n.a.	Mut	WT	Mut	n.a.	n.a.	n.a.	n.a.	n.a.	Mut	Mut	n.a.	n.a.	n.a.	Mut	Mut	n.a.	Mut	WT	Mut
Grading	G2	G2	G3	G2	G2	G2	G2	G2	G2	G2	G2	G3	G2	G2	G3	G3	G2	G3	G2	n.a.	n.a.	G2
TRG grade	n.a.	n.a.	n.a.	n.a.	n.a.	n.a.	n.a.	n.a.	n.a.	n.a.	n.a.	n.a.	n.a.	n.a.	n.a.	n.a.	n.a.	n.a.	n.a.	2	2	2
Microsatellite Status	MSS	n.d.	MSS	MSS	MSS	MSS	MSS	MSS	n.d.	n.d.	n.d.	n.d.	n.d.	n.d.	n.d.	n.d.	n.d.	n.d.	n.d.	n.d.	MSS	MSS
NeoAdjuvant Chemo	naïve	naïve	naïve	naïve	naïve	naïve	naïve	naïve	naïve	naïve	naïve	naïve	naïve	naïve	naïve	naïve	naïve	naïve	naïve	FOLFOXIRI + Bev	FOLFOX + Bev	Cape
IFNAR1 on Liver (IHC)	High	High	Low	High	High	High	High	High	Low	Low	Low	Low	Low	n.a.	High	Low	Low	High	Low	Low	Low	Low

	CRC-LM26	CRC-LM27	CRC-LM28	CRC-LM29	CRC-LM30	CRC-LM31	CRC-LM32	CRC-LM33	CRC-LM35	CRC-LM36	CRC-LM37	CRC-LM38	CRC-LM39	CRC-LM40	CRC-LM41	CRC-LM42	CRC-LM43	CRC-LM46	CRC-LM47	CRC-LM49	CRC-LM50	CRC-LM51
Age	72	70	70	54	63	52	57	75	72	84	51	61	72	73	78	78	54	33	65	78	82	73
Sex	f	m	f	m	m	f	f	m	m	m	m	m	m	m	m	f	m	m	m	f	m	m
Tumor Site	Right	Left	Left	Right	Left	Left	Left	Left	Right	Left	Right	Left	Left	Left	Left	Left	Left	Left	Left	Left	Left	Left
KRAS	Mut	n.a.	WT	Mut	Mut	WT	Mut	n.a.	n.a.	WT	n.a.	n.a.	n.a.	WT	n.a.	n.a.	n.a.	WT	WT	Mut	WT	n.a.
Grading	n.a.	n.a.	n.a.	n.a.	n.a.	n.a.	G2	G2	G3	G2	G2	G2	G2	G2	G2	G1	G2	G2	n.a.	n.a.	G2	G3
TRG grade	3	2	1	1	3	2	3	n.a.	n.a.	n.a.	n.a.	n.a.	n.a.	n.a.	n.a.	3	n.a.	n.a.	3	3	n.a.	n.a.
Microsatellite Status	n.d.	MSS	MSS	MSS	n.d.	n.d.	n.d.	MSS	n.d.	n.d.	n.d.	n.d.	n.d.	n.d.	n.d.	n.d.	n.d.	n.d.	n.d.	n.d.	n.d.	n.d.
NeoAdjuvant Chemo	FOLFOX + Bev	FOLFOX	CAPOX + Bev	FOLFIRI + Bev	Bev	FOLFOX	FOLFOX6	FOLFIRI + Cet	FOLFOX4 + Pani	FOLFOX6	n.a.	FOLFIRI + Bev	FOLFOX	FOLFOX + Bev	n.a.	n.a.	n.a.	FOLFIRI + Bev	FOLFOX6 + Cet	CAPOX	CAPOX	FOLFIRI + Cet
IFNAR1 on Liver (IHC)	Low	Low	Low	High	Low	Low	Low	Low	n.a.	Low	High	Low	Low	Low	Low	Low	Low	Low	Low	High	Low	Low

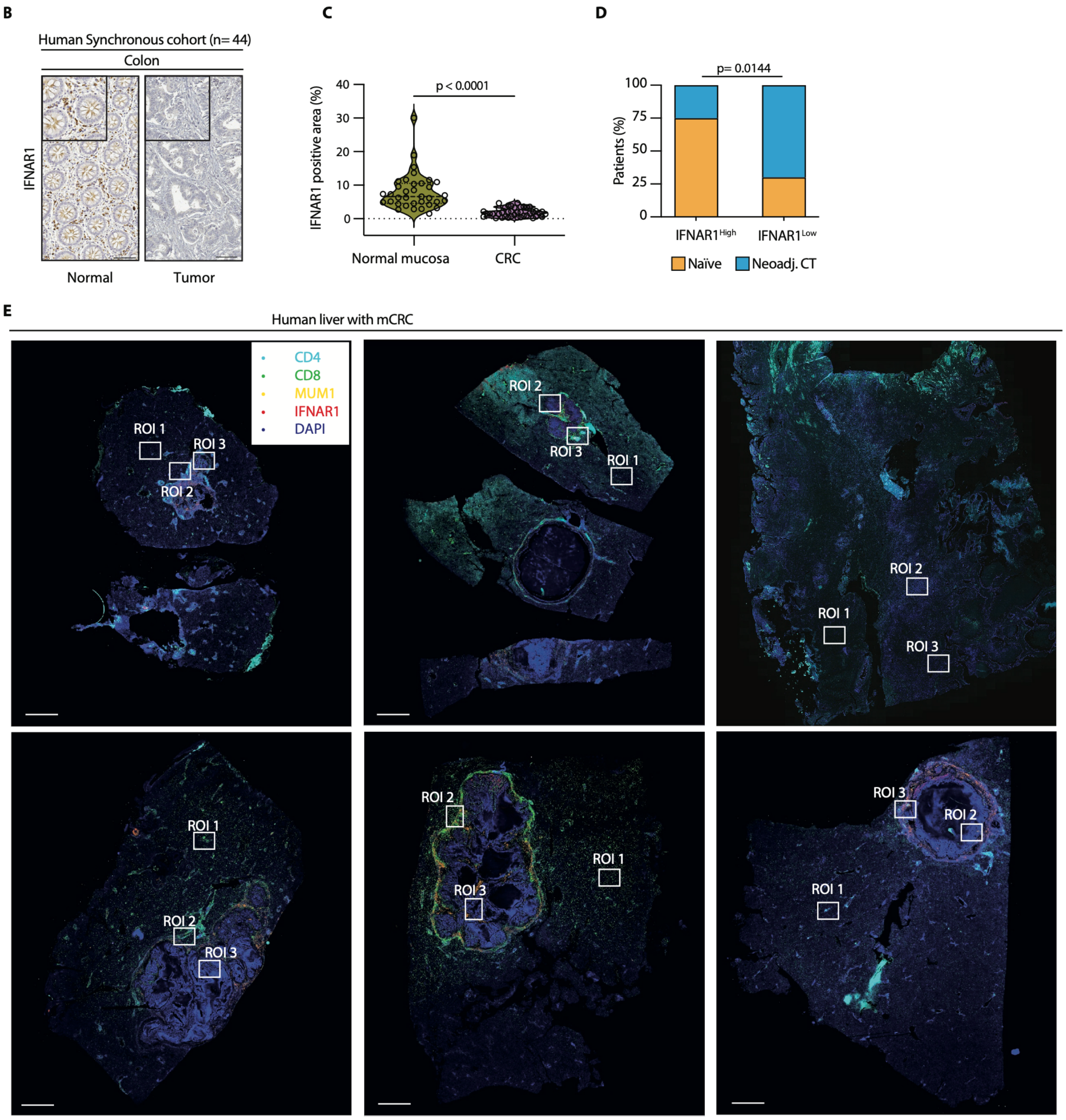

Fig. S1. Clinicopathological features of the synchronous CRC cohort and IFNAR1 status from primary CRC to liver metastases.

(A) Clinical and pathological characteristics of the Human Synchronous cohort (n = 44). Parameters include age, sex, primary tumor site (right colon, left colon, or rectum)**,** KRAS mutation status, grade (G1-G3), tumor regression grade (TRG), microsatellite status (MSS/MSI),

and neoadjuvant chemotherapy exposure with drug regimen. Chemotherapy/targeted-therapy abbreviations: 5-FU, 5-fluorouracil; LV, leucovorin/folinic acid; Cape, capecitabine; Oxa, oxaliplatin; Iri, irinotecan; Bev (Avastin), bevacizumab; Cet (Erbitux), cetuximab; Pani, panitumumab; FOLFOX = 5-FU/LV + oxaliplatin; FOLFIRI = 5-FU/LV + irinotecan; FOLFOXIRI = 5-FU/LV + oxaliplatin + irinotecan; CAPOX = capecitabine + oxaliplatin. Regimens highlighted in blue indicate treatments previously reported to induce immunogenic cell death. IFNAR1 status at the liver peri-metastatic invasive front IFNAR1^{High} (in orange) vs IFNAR1^{Low} (in green) is annotated for each patient. n.a., not available; n.d., not determined. **(B)** Representative IFNAR1 immunohistochemistry (IHC) in normal colon mucosa and matched primary CRC tumor epithelium from the Human Synchronous cohort (n=44). Scale bar, 100 µm; Insets show magnified regions. **(C)** Quantification of the IFNAR1-positive area (%) in normal colon mucosa versus CRC regions shown in (B). Each dot represents one patient; The exact P value was determined using a two-sided Wilcoxon matched-pairs signed-rank test; $P < 0.0001$. **(D)** Association between peri-metastatic IFNAR1 status and neoadjuvant chemotherapy (CT) exposure in patients with evaluable peri-metastatic IFNAR1 expression (n = 42). The stacked bar plot shows the proportions of treatment-naïve and neoadjuvant CT-treated patients within the IFNAR1^{High} and IFNAR1^{Low} groups. IFNAR1^{Low} patients had higher odds of having received neoadjuvant CT than IFNAR1^{High} patients (OR, 7.00; 95% CI, 1.49-27.02; two-sided Fisher's exact test, P = 0.0144; Baptista-Pike method for the confidence interval). **(E)** Whole-section multispectral images of additional IFNAR1^{High} CRC liver metastases stained for CD4 (cyan), CD8 (green), MUM1/IRF4 (yellow), and IFNAR1 (red), with DAPI nuclear counterstain (blue). For each liver section, three ROIs (ROI 1-3) indicate uninvolved parenchyma, peri-metastatic invasive front, and intra-metastatic tumor regions. Scale bars, 2 mm.

A

	PDO#6	PDO#8	PDO#9	PDO#11	PDO#17	PDO#20	PDO#22	PDO#39	PDO#43	PDO#49	PDO#54	PDO#61	PDO#62	PDO#68	PDO#74
Age	70	66	64	72	74	69	66	58	64	65	64	81	51	58	73
Sex	m	m	m	m	m	f	m	f	m	f	m	m	m	f	f
Tumor Site	Left	Right	Rectum	Rectum	Left	Left	Rectum	Left	Left	Left	Rectum	Left	Rectum	Rectum	Right
KRAS	n.a.	wt	wt	wt	mut	wt	n.a.	wt	n.a.	mut	wt	mut	mut	n.a.	mut
Grading	G3	G3	G2	G2	G2	G3	n.a.	G2	G2	G3	G2	G3	G3	G2	G2
TRG grade	4	2	4	4	3	2	4	n.a.	4	n.a.	n.a.	n.a.	n.a.	n.a.	n.a.
Microsatellite Status	n.a.	n.a.	n.a.	n.a.	n.a.	n.a.	n.a.	MSS	MSS	MSS	n.a.	n.a.	MSI	n.a.	MSS
NeoAdjuvant Chemo	naïve	naïve	FOLFIRI + Cet	CAPOX	naïve	FOLFOX + Pani	FOLFIRI + Afli	naïve	FOLFIRI + Pani	naïve	naïve	naïve	FOLFIRI + Cet	naïve	naïve
IFNAR1 on Liver (IHC)	n.a.	n.a.	n.a.	n.a.	n.a.	n.a.	n.a.	Low	Low	Low	High	High	Low	High	Low

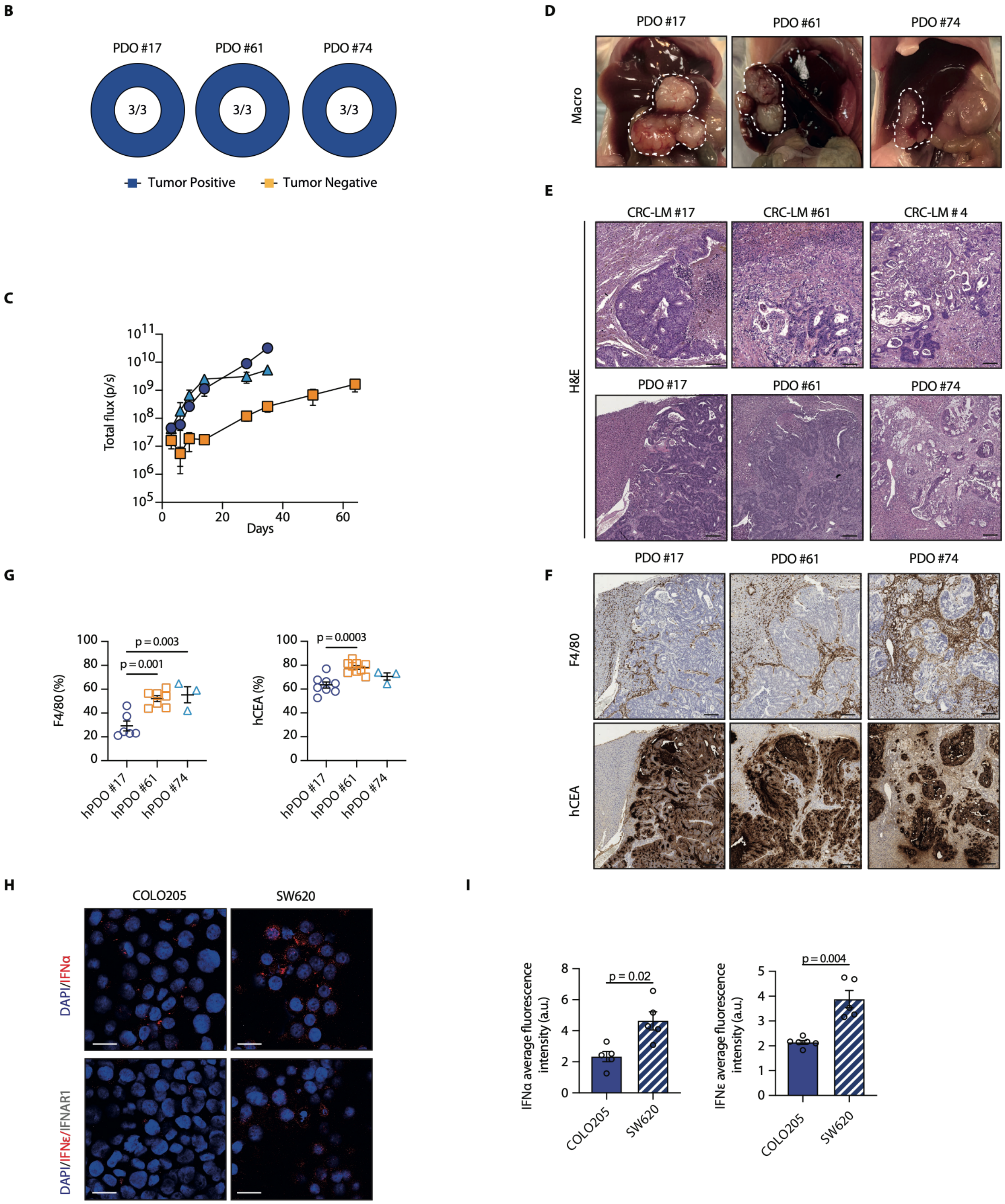

Fig. S2. PDO models of CRC liver metastases and associated histopathological characterization.

(**A**) Clinical and pathological characteristics of the ACC_ORG-LiMeT PDO cohort **(**n = 15 independent PDO lines**)**, including age, sex, primary tumor site, KRAS mutational status, histological grade, tumor regression grade (TRG), microsatellite status, neoadjuvant treatment regimen, and IFNAR1 expression assessed by immunohistochemistry (IHC) in the corresponding available CRC liver metastasis specimens. Chemotherapy/targeted-therapy abbreviations: 5-FU, 5-fluorouracil; LV, leucovorin/folinic acid; Cape, capecitabine; Oxa, oxaliplatin; Iri, irinotecan; Cet (Erbitux), cetuximab; Pani, panitumumab; Afli, aflibercept; FOLFIRI = 5-FU/LV + irinotecan; FOLFOX = 5-FU/LV + oxaliplatin; CAPOX = capecitabine + oxaliplatin. Regimens highlighted in blue indicate treatments previously reported to induce immunogenic cell death. n.a., not available. Histological material for IFNAR1 IHC was not available for 7 of 15 PDO cases. PDOs in bold (#17, #61, #74) were used for *in vitro* and *in vivo* validation assays. (**B**) Incidence of tumor outgrowth following intrahepatic implantation of luciferase-expressing PDO #17, #61, and #74 into NOD-SCID mice (n = 3 mice per PDO line). (**C**) Longitudinal bioluminescence imaging (IVIS) of tumor burden following intrahepatic implantation of luciferase-expressing PDO lines in the mice described in (B). Total flux is expressed as photons/s. Data are shown as mean ± SEM (n = 3 mice per PDO line). (**D**) Representative macroscopic images of livers at endpoint showing hepatic tumor lesions (dashed lines) of PDO #17, #61, or #74. (**E**) Representative H&E staining of the original human liver metastasis specimens and corresponding PDO-derived mouse liver lesions (PDO #17, PDO #61, and PDO #74; bottom). Scale bar, 200 µm. (**F**) Representative immunohistochemistry of murine liver metastases (CRC-LMs) generated by intrahepatic implantation of PDOs, stained for F4/80 (top), and human CEA (hCEA, bottom). Scale bar, 200 µm. (**G**) Quantification of F4/80-positive area (%, left), and hCEA-positive area (%, right) in murine liver lesions derived from PDO #17, #61, and #74. Each dot represents one metastatic lesion **(**F4/80: n = 6, 7, and 3 lesions for PDO #17, #61, and #74, respectively; hCEA: n = 8, 9, and 3 lesions, respectively); bars show mean ± SEM. Statistical significance was assessed using one-way ANOVA with Tukey's multiple-comparisons test; P values are shown. (**H**) Representative immunofluorescence of pan-IFNα (red; top) or IFNε (red; bottom) together with IFNAR1 (white; bottom) in human CRC cell lines derived from metastatic lesions (COLO205, SW620), with DAPI nuclear counterstain (blue). Scale bar, 50 µm. (**I**) Quantification of the mean fluorescence intensity (MFI; arbitrary units, a.u.) of pan-IFNα (left) and IFNε (right) in the CRC cell lines shown in (H). Each dot represents one randomly selected field of view (n = 4 fields per cell line), and bars indicate mean ± SEM. Statistical significance was assessed using a two-sided Mann-Whitney U test; P values are shown.

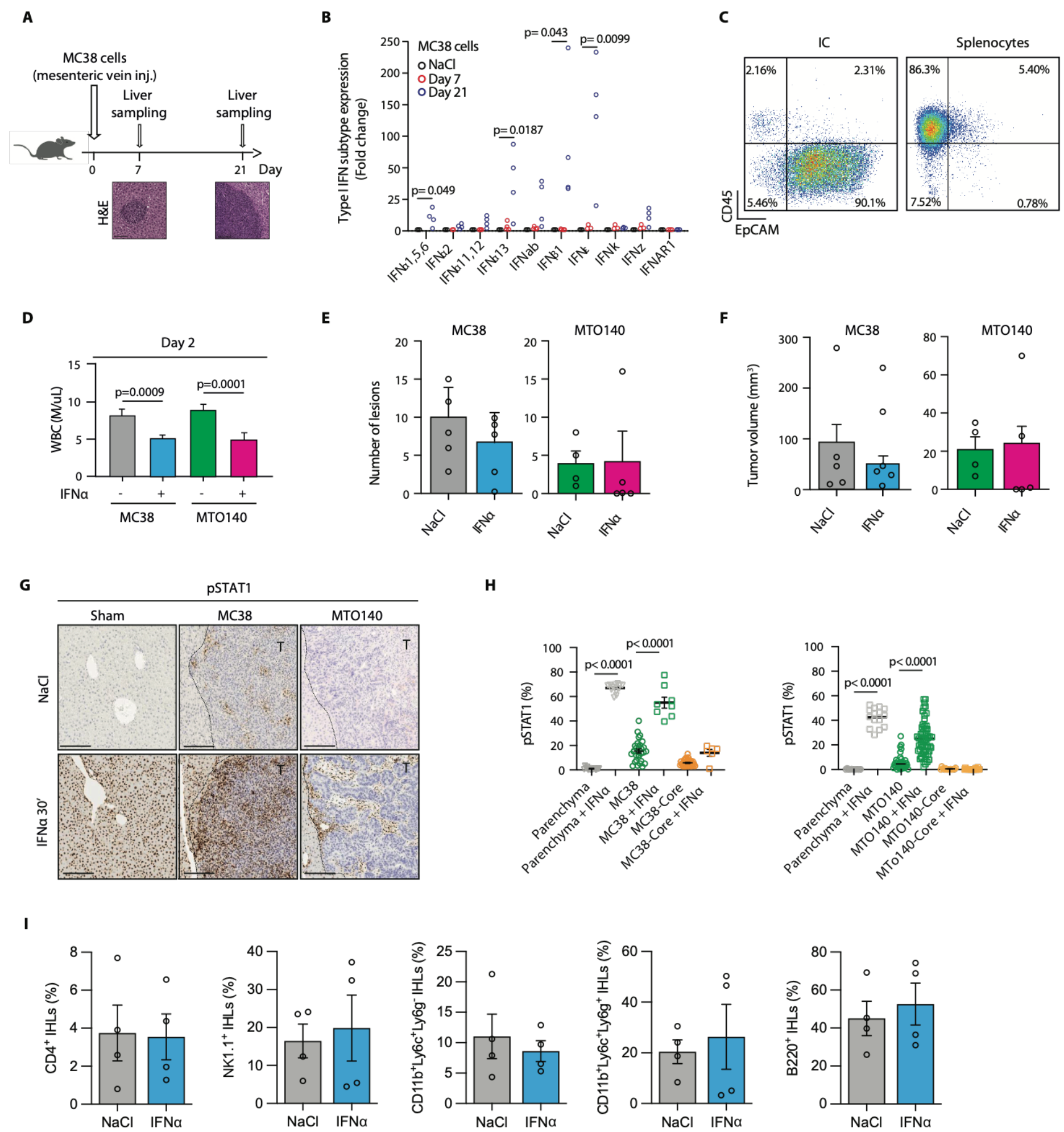

Fig. S3. Time-dependent type I IFN expression and signaling in murine CRC liver metastasis models.

(**A**) Schematic of the experimental design for MC38 liver metastasis induction by mesenteric vein injection, with liver collection at day 7 and day 21 post-injection. Representative hematoxylin and eosin (H&E) images at the indicated time points are shown. Scale bar, 100 µm. (**B**) Fold change in all type I IFN-related transcript expression in livers from MC38-bearing mice at day 7 (red) and day 21 (blue) post-injection, normalized to NaCl controls, quantified using TaqMan Array Cards. Note: *Ifnα4*, *Ifnα7*, *Ifnα9*, *Ifnα14*, *Ifnα15*, and *Ifnα16* transcripts were below the limit of detection in this assay and are not shown. Each dot represents one mouse. Kruskal-Wallis test with Dunn's multiple-comparisons correction; exact P values are shown. (**C**) Representative flow cytometry gating strategy for isolation of murine intestinal crypt epithelial cells (IC) using EpCAM (epithelial marker) and CD45 (immune marker). Splenocytes were used as a positive control for CD45 staining. Percentages of cells in each quadrant are indicated. (**D**) Peripheral white blood cell (WBC) counts measured 2 days after treatment initiation in mice bearing MC38 or MTO140 liver metastases treated with continuous IFNα or NaCl control (as in Fig. 3F). Bars show mean ± SD. Two-way ANOVA followed by Šídák's multiple-comparisons test: P values are shown. (**E-F**) Quantification of the number (E) and of total liver metastatic burden (tumor volume, mm³) (F) from contrast-enhanced MRI in MC38- and MTO140-bearing mice treated with NaCl or IFNα (Fig. 3G). Each dot represents one mouse (MC38: NaCl, n = 5; IFNα, n = 5; MTO140: NaCl, n =

4; IFNα, n = 5), and bars indicate mean ± SEM. (**G**) Representative immunohistochemistry for pSTAT1 (Tyr701) in livers from sham, MC38, and MTO140 cohorts following acute (30 min) high dose IFNα (1000 ng) i.p. challenge at endpoint (top, NaCl; bottom, IFNα 30′). Dashed lines demarcate tumor margins (T). Scale bar, 200 µm. (**H**) Quantification of $pSTAT1^+$ cells (%) in liver compartments from MC38 (left) or MTO140 (right) models following acute high-dose IFNα challenge (30 min) at endpoint (day 28), comparing liver in peritumoral (green) and intratumoral core regions (orange). Each dot represents one quantified tumor/ROI. One-way ANOVA + Tukey multiple-comparisons correction; exact P values are shown. (**I**) Frequencies of the indicated immune-cell populations among live intrahepatic leukocytes (IHLs) isolated from MC38-bearing mice at day 21 after tumor-cell injection and treated with NaCl or IFNα, as described in Fig. 3F. Bar plots show $CD3^+CD4^+$ T cells, $NK1.1^+$ NK cells, $CD11b^+Ly6C^+Ly6G^-$ monocytes, $CD11b^+Ly6C^+Ly6G^+$ neutrophils, and $B220^+$ B cells. Each dot represents one mouse (n = 4 mice per group), and bars indicate mean ± SEM. Statistical significance was assessed using two-sided Mann-Whitney tests; all comparisons were not significant.

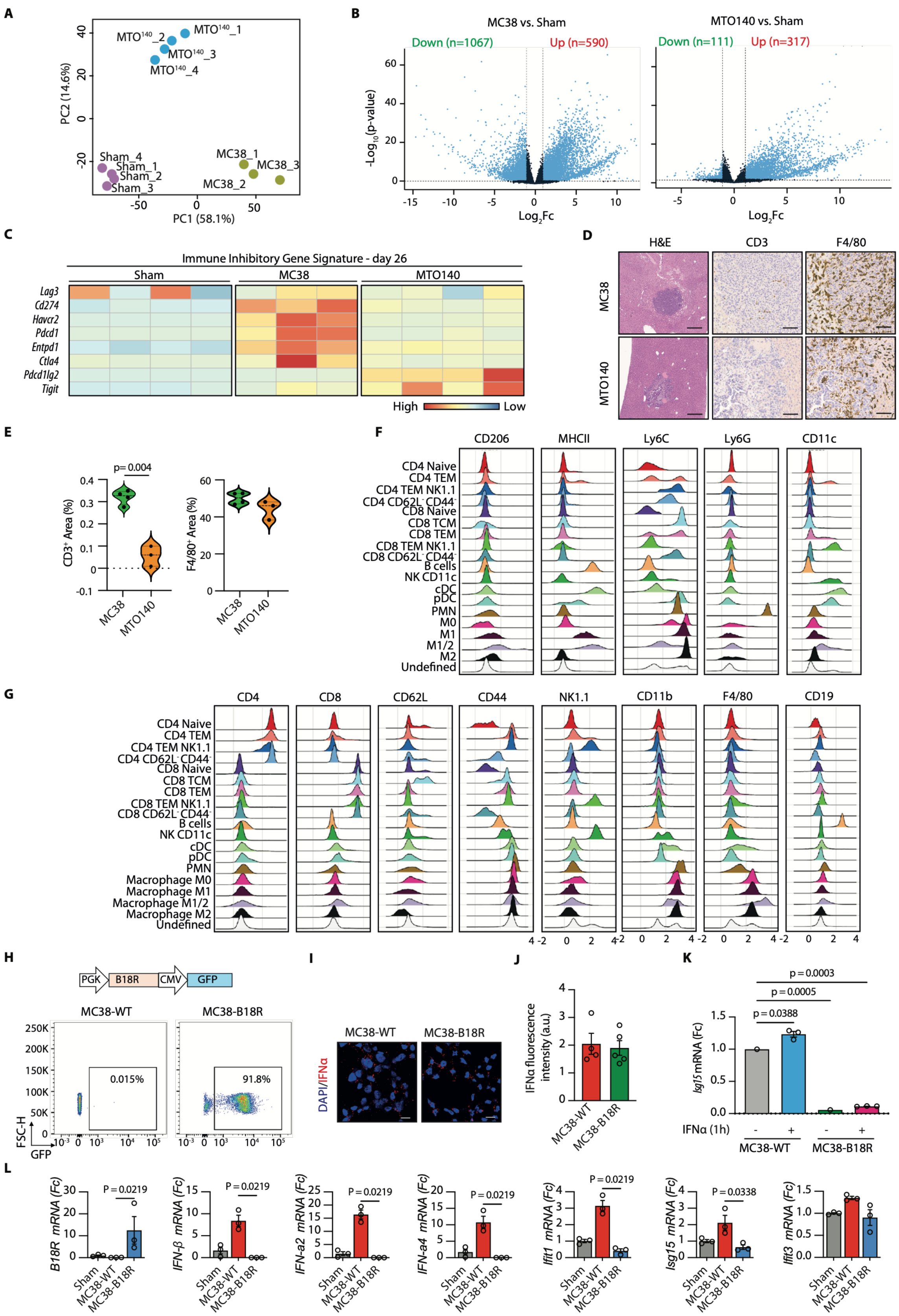

A
PC1 (58.1%)
PC2 (14.6%)
MTO140_1
MTO140_2
MTO140_3
MTO140_4
Sham_1
Sham_2
Sham_3
Sham_4
MC38_1
MC38_2
MC38_3
B
MC38 vs. Sham
Down (n=1067)
Up (n=590)
MTO140 vs. Sham
Down (n=111)
Up (n=317)
-Log10 (p-value)
Log2Fc
C
Immune Inhibitory Gene Signature - day 26
Sham
MC38
MTO140
Lag3
Cd274
Havcr2
Pdcd1
Entpd1
Ctla4
Pdcd1lg2
Tigit
High
Low
D
H&E
CD3
F4/80
MC38
MTO140
E
p=0.004
CD3+ Area (%)
F4/80+ Area (%)
MC38
MTO140
F
CD206
MHCII
Ly6C
Ly6G
CD11c
CD4 Naive
CD4 TEM
CD4 TEM NK1.1
CD4 CD62L- CD44-
CD8 Naive
CD8 TCM
CD8 TEM
CD8 TEM NK1.1
CD8 CD62L- CD44-
B cells
NK CD11c
cDC
pDC
PMN
M0
M1
M1/2
M2
Undefined
G
CD4
CD8
CD62L
CD44
NK1.1
CD11b
F4/80
CD19
Macrophage M0
Macrophage M1
Macrophage M1/2
Macrophage M2
H
PGK
B18R
CMV
GFP
MC38-WT
MC38-B18R
0.015%
91.8%
FSC-H
GFP
I
MC38-WT
MC38-B18R
DAPI/IFNα
J
IFNα fluorescence intensity (a.u.)
MC38-WT
MC38-B18R
K
p = 0.0003
p = 0.0005
p = 0.0388
Isg15 mRNA (Fc)
IFNα (1h)
MC38-WT
MC38-B18R
L
B18R mRNA (Fc)
IFN-β mRNA (Fc)
IFN-a2 mRNA (Fc)
IFN-a4 mRNA (Fc)
Ifit1 mRNA (Fc)
Isg15 mRNA (Fc)
Ifit3 mRNA (Fc)
P = 0.0219
P = 0.0338
Sham
MC38-WT
MC38-B18R

Fig. S4. Transcriptional and immune profiling of CRC liver metastases and validation of B18R-mediated extracellular type I IFN neutralization.

(**A**) Principal component analysis (PCA) of bulk RNA-seq profiles from Sham (pink), MC38 (light green)- and MTO140 (light blue)-bearing livers at day 26, showing distribution of samples from each group, based on gene expression profiles (Sham, n = 4; MC38, n = 3; MTO140, n = 4). (**B**) Volcano plots of differential gene expression (DGE) from bulk RNA-seq comparing MC38-bearing *versus* sham livers (left) and MTO140-bearing *versus* sham livers (right) at day 26 (MC38, n = 3; MTO140, n = 4; sham, n = 4). Differentially expressed genes were identified using (FDR < 0.01; $\log_2$(Fold change) > 1 or < -1). Numbers of significantly upregulated (red) and downregulated (green) genes are indicated. (**C**) Heatmap showing the relative expression of selected immune-inhibitory genes in sham, MC38-bearing, and MTO140-bearing livers at day 26. Expression values were calculated from $\log_2$-transformed RPKM values and scaled by gene across samples. **(D)** Representative H&E, CD3 and F4/80 immunohistochemical staining of MC38 and MTO140 liver metastases at day 21 after injection. Scale bars: H&E, 500 µm; CD3 and F4/80, 100 µm. (**E)** Violin plots showing $CD3^+$ area (%) and $F4/80^+$ area within MC38 and MTO140 metastatic lesions described in (D). Each dot represents one metastatic lesion. Unpaired t test; P value is shown. (**F-G**) Ridge plots showing the expression distributions of the indicated lineage and phenotypic markers across the immune-cell populations identified by unsupervised UMAP analysis of intrahepatic leukocytes from sham and MC38-bearing livers on day 14. The marker-expression profiles were used to annotate the clusters shown in Fig. 4D. TEM, effector memory T cells; TCM, central memory T cells; cDC, conventional dendritic cells; mDC, myeloid dendritic cells; pDC, plasmacytoid dendritic cells; PMN, polymorphonuclear cells; M0, M1, M1/M2, and M2, macrophage subsets. (**H**) Schematic of the lentiviral construct used to generate MC38-B18R-eGFP cells, encoding the vaccinia virus type I IFN decoy receptor B18R and GFP under independent promoters (top). Representative flow-cytometry plots showing GFP expression in parental MC38-WT cells and MC38-B18R live cells after transduction (bottom). Percentages of GFP-positive cells are indicated. (**I**) Representative immunofluorescence staining for pan-IFNα in MC38-WT and MC38-B18R cells, with DAPI nuclear counterstain. Scale bar, 50 µm. (**J**) Quantification of the mean pan-IFNα fluorescence intensity (arbitrary units, a.u.) in the MC38-WT and MC38-B18R cells shown in (I). Each dot represents one randomly selected field of view (MC38-WT, n = 4 fields; MC38-B18R, n = 5 fields), and bars indicate mean ± SEM. Statistical significance was assessed using a two-sided Mann-Whitney test. (**K**) qPCR analysis of *Isg15* mRNA in MC38-WT and MC38-B18R cells following acute IFNα stimulation (200 pg/mL for 1 hour**)**. Data are shown as fold change (FC) relative to unstimulated controls $2^{-\Delta\Delta Ct}$. One-way ANOVA followed by Dunnett's multiple-comparisons test was used for statistical analysis. P values are shown. (**L)** Quantification of the relative expression of B18R, type I IFN transcripts (*Ifnβ1*, *Ifnα2*, *Ifnα4*) and representative ISGs (*Ifit1*, *Isg15*, *Ifit3*) in mRNA extracted from whole-liver tissue from sham mice and mice injected via the mesenteric vein with MC38-WT or MC38-B18R cells and analyzed on day 26 (n = 3 mice per group). Relative mRNA expression was calculated using the $2^{-\Delta\Delta Ct}$ method. Each dot represents one mouse, and data are shown as mean ± SEM. For each gene, differences among groups were assessed using a Kruskal-Wallis test followed by Dunn's multiple-comparisons test. P values are shown.

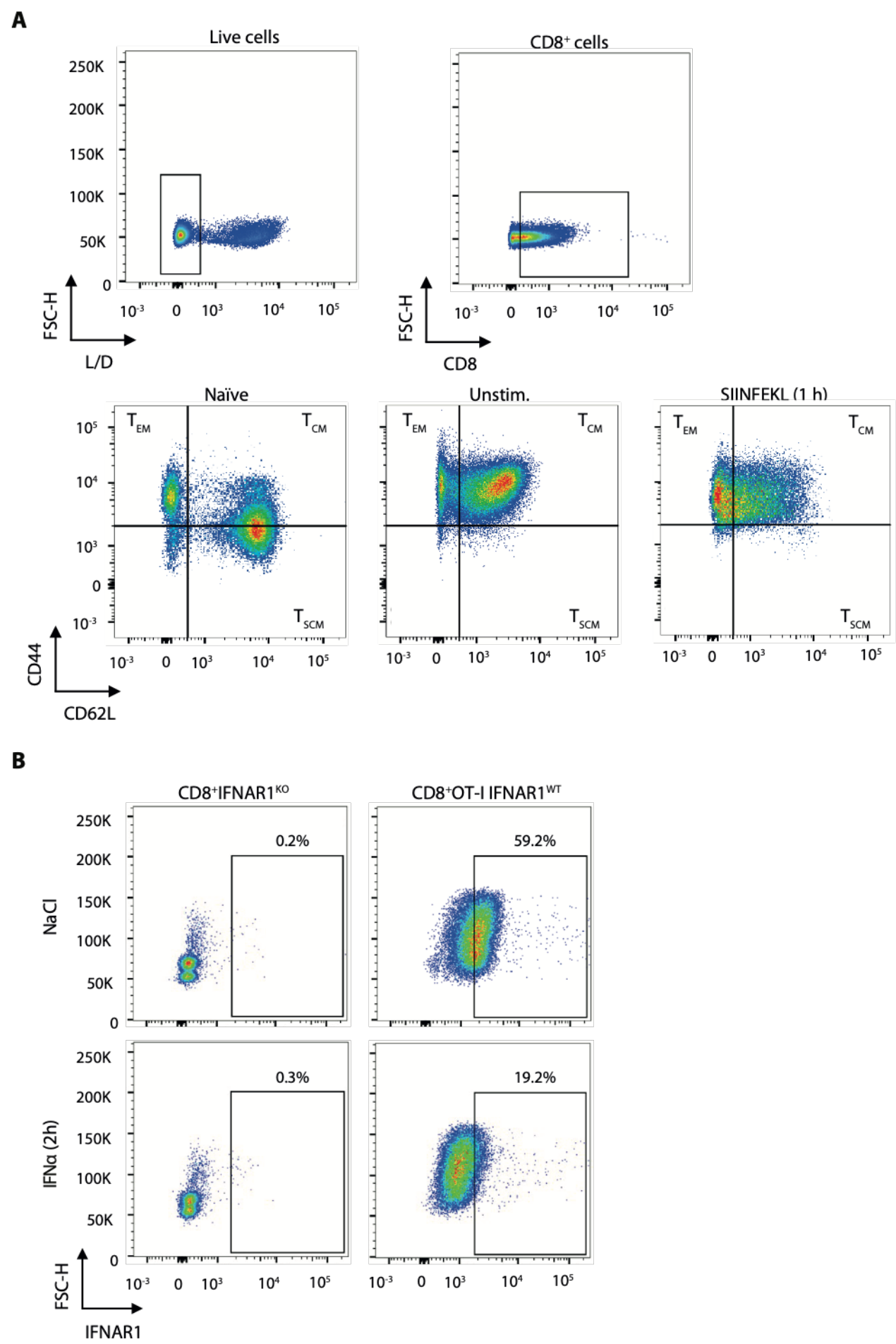

Fig. S5. Flow cytometry gating strategy for phenotypic characterization of activated and expanded OT-I CD8⁺ T cells and for surface IFNAR1 detection.

(**A**) Representative flow cytometry plots showing the sequential gating of live $CD8^+$ cells and the identification of effector memory T cells (T_{EM}), central memory T cells (T_{CM}), and T stem cell memory (T_{SCM}) in naïve cells and in day-6 OT-I cells left unstimulated or restimulated with SIINFEKL (1 µg/mL) for 1 hour. T_{EM}, $CD8^+CD44^{high}CD62L^{Low}$; T_{CM}, $CD8^+CD44^{high}CD62L^{high}$; T_{SCM}, $CD8^+$ $CD44^{low}CD62L^{high}$. Related to Fig. 5B. (**B**) Representative flow cytometry plots showing the gating strategy for surface IFNAR1 detection in live $CD8^+$ OT-I $IFNAR1^{WT}$ cells following treatment with IFNα (200 pg/mL) or NaCl vehicle for 2 hours. $CD8^+$ $IFNAR1^{KO}$ splenocytes were used as a negative control to define the IFNAR1-positive gate. Numbers indicate the percentage of $IFNAR1^+$ cells within the gated population. Related to Fig. 5D.

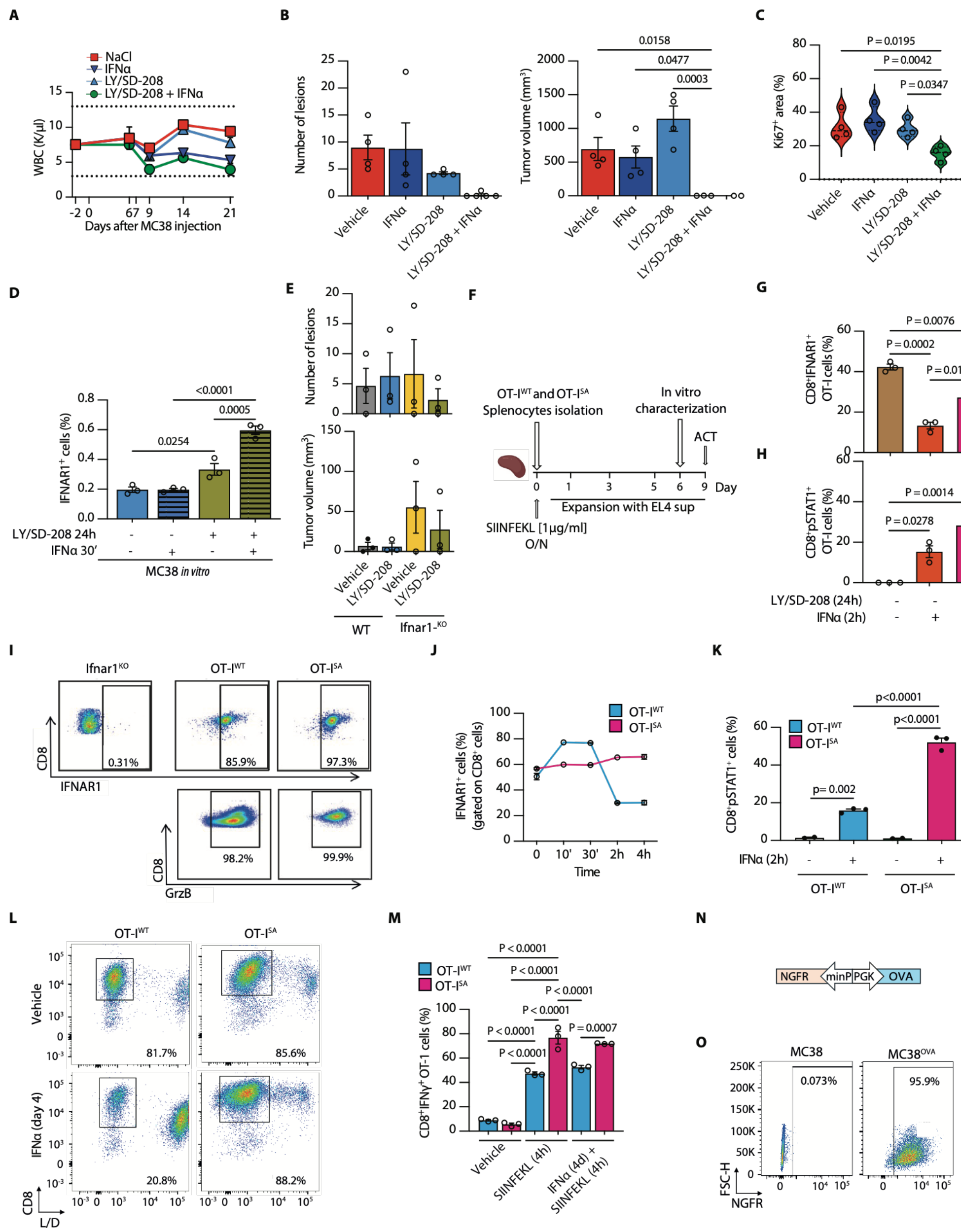

Fig. S6. Additional characterization of LY/SD-208 plus IFNα therapy and IFNAR1-stabilized OT-I cells.

(**A**) Peripheral white blood cell (WBC) counts over time in MC38-bearing C57BL/6 mice treated with NaCl (n = 4), IFNα (n = 4), LY/SD-208 (n = 4), or LY/SD-208 plus IFNα (n = 6). Data are shown as mean ± SEM. The dashed lines indicate the physiological range. (**B**) Quantification of the number of liver metastatic lesions (left) and total tumor volume (mm³; right) in MC38-bearing C57BL/6 mice treated with NaCl (n = 4), IFNα (n = 4), LY/SD-208 (n = 4), or LY/SD-208 plus IFNα (n = 5). Each dot represents one mouse. Data are shown as mean ± SEM. P values were determined by ordinary one-way ANOVA followed by Tukey's multiple-comparisons test. (**C**) Quantification of Ki-67⁺ area (%) within liver lesions from mice treated with vehicle, IFNα, LY/SD-208, or LY/SD-208 plus IFNα. Each dot represents the mean value across all metastatic

lesions analyzed in each individual mouse. Violin plots show the distribution, with mean. P values were determined by ordinary one-way ANOVA followed by Tukey's multiple-comparisons test.
(**D**) Quantification of IFNAR1^{+} MC38 cells (%) by flow cytometry following acute IFNα stimulation for 30 min, with or without 24-hour pretreatment with LY/SD-208 (n = 3 independent replicates per condition). Data are shown as mean ± SEM. P values were determined by one-way ANOVA followed by Tukey's multiple-comparisons test**.**
(**E**) C57BL/6 WT and global Ifnar1^{KO} mice were injected with MC38 cells via the mesenteric vein on day 0 to establish liver metastases. Beginning on day 7, mice received vehicle or LY/SD-208 by oral gavage. Treatments continued until day 21, when tumor burden was assessed by MRI. Graphs show the number of hepatic metastatic lesions (top) and total tumor volume (bottom). Each dot represents one mouse (n = 3 mice per group), and bars show mean ± SEM.
(**F**) Generation of effector OT-I CD8^{+} T cells (OT-I^{WT} and OT-I^{SA}) and schedule for *in vitro* characterization and adoptive cell transfer (ACT). Splenocytes from OT-I^{WT} or OT-I^{SA} mice were stimulated overnight (O/N) with SIINFEKL peptide (1 μg/mL). Cells were expanded in EL4 supernatant as indicated, with medium replenished every 2 days**,** phenotypically characterized by flow cytometry, and used for in vitro assays on day 6 or injected intravenously (i.v.) for ACT on day 9**.** (**G-H**) Flow cytometry quantification of surface IFNAR1^{+} cells (G) and pSTAT1 (Tyr701)$^{+}$ cells (H) among live CD8^{+} OT-I^{WT} cells following 24-hour pretreatment with LY/SD-208 and subsequent stimulation with IFNα for 2 hours**.** Each dot represents one independent replicate (n = 3 per condition). Data are shown as mean ± SEM. P values were determined by ordinary one-way ANOVA followed by Tukey's multiple-comparisons test. (**I**) Representative flow cytometry plots showing surface IFNAR1 staining (top) in IFNAR1^{KO} splenocytes and OT-I^{WT} and OT-I^{SA} effector CD8^{+} T cells**.** IFNAR1^{KO} splenocytes were used as a negative control to define the IFNAR1-positive gate**.** Representative intracellular Granzyme B (GrzB) staining (bottom) is shown for OT-I^{WT} and OT-I^{SA} CD8^{+} T cells**.** Numbers indicate the percentage of cells within the indicated gates**.**
(J) Time-course analysis showing the percentage of surface IFNAR1^{+} cells among CD8^{+} OT-I^{WT} and OT-I^{SA} effector T cells following in vitro IFNα stimulation (200 pg/mL; n = 3 independent replicates per genotype at each time point). Data are shown as mean ± SEM. P values were determined by two-way ANOVA. (**K**) Percentage of pSTAT1 (Tyr701)$^{+}$ cells among OT-I^{WT} and OT-I^{SA} effector CD8^{+} T_EM cells after 2 hours of IFNα stimulation (OT-I^{WT} vehicle, n = 2; OT-I^{WT} IFNα, n = 3; OT-I^{SA} vehicle, n = 2; OT-I^{SA} IFNα, n = 3). Data are shown as mean ± SEM. P values were determined by ordinary one-way ANOVA followed by Tukey's multiple-comparisons test. (**L**) Representative flow cytometry plots showing the viability of OT-I^{WT} and OT-I^{SA} CD8^{+} T cells following vehicle treatment or chronic IFNα exposure (50 pg/mL for 4 days)**.** Live cells were identified by live/dead staining. Numbers indicate the frequency (%) of live CD8^{+} cells within the indicated gates. (**M**) Quantification of IFNγ^{+} cells among live OT-I^{WT} and OT-I^{SA} CD8^{+} T cells, gated as shown in (L), following SIINFEKL restimulation for 4 hours under the indicated conditions (n = 3 independent replicates per condition). Data are shown as mean ± SEM. P values were determined by ordinary one-way ANOVA followed by Tukey's multiple-comparisons test. (**N**) Schematic of the lentiviral construct used to generate MC38^{OVA} cells. NGFR and OVA expression was driven by the bidirectional minP/PGK promoter cassette. (**O**) Representative flow cytometry plots showing NGFR expression in parental MC38 and MC38^{OVA} cells following lentiviral transduction. Numbers indicate the percentage of cells within the indicated gate.

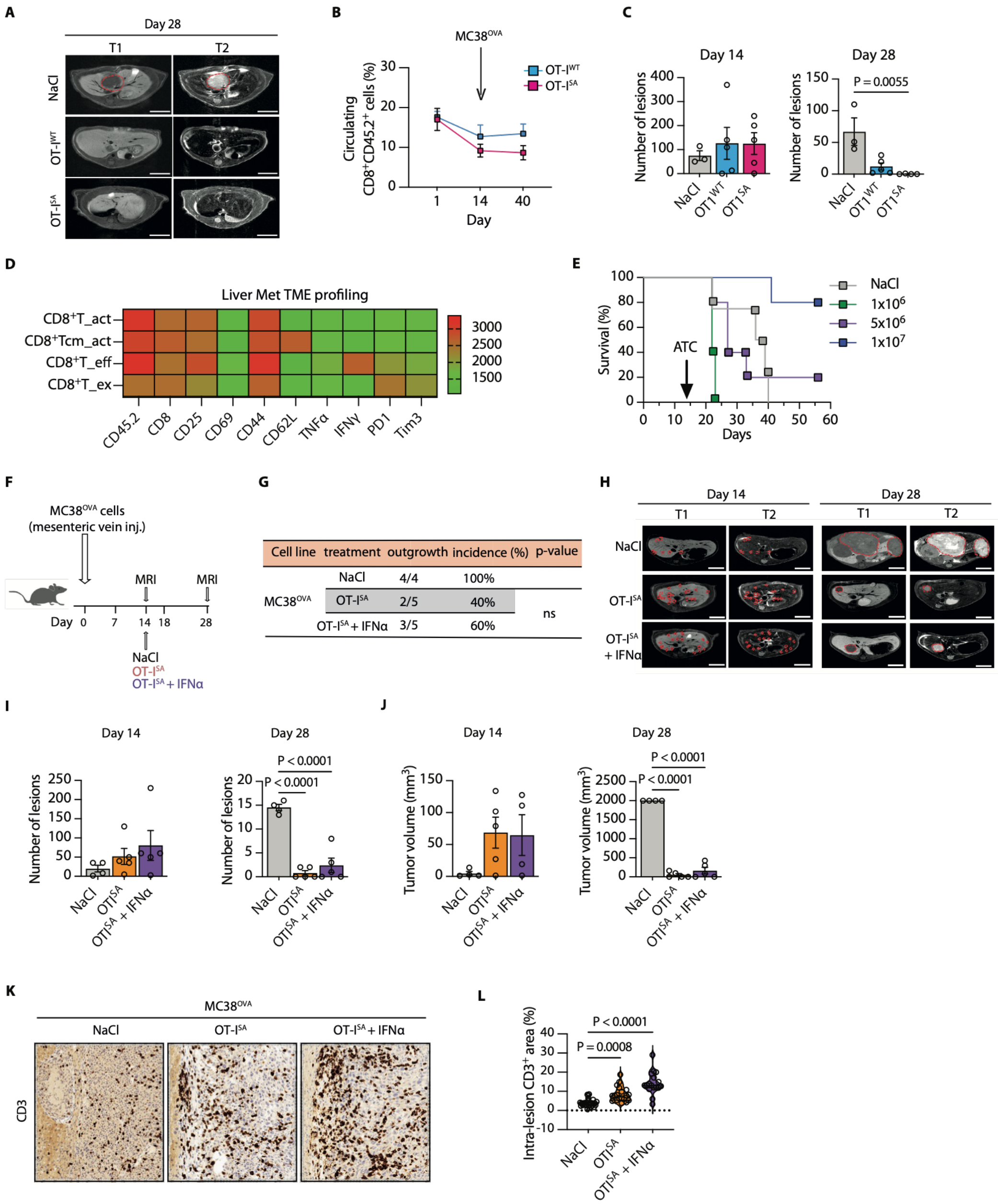

Fig. S7. Early and established ACT efficacy, OT-I^{SA} dose titration, and the effects of continuous IFNα administration combined with subcurative OT-I^{SA} ACT in MC38^{OVA} liver metastases.

(**A**) Representative contrast-enhanced T1- and T2-weighted magnetic resonance images acquired at day 28 from mice bearing MC38^{OVA} liver metastases and treated with NaCl, OT-I^{WT}, or OT-I^{SA} ACT at day 7 after tumor-cell injection. Red dashed lines delineate metastatic lesions. Scale bars, 5 mm. (**B**) Longitudinal frequency of donor OT-I^{WT} and OT-I^{SA} cells among circulating CD8^{+}CD45.2^{+} T cells following secondary transfer into naïve congenic recipients. The arrow indicates MC38^{OVA} challenge by mesenteric-vein injection at day 14. Data are shown as mean ± SEM. (**C**) MRI-based quantification of hepatic metastatic lesion number at day 14, before ACT, and at day 28 in mice treated with NaCl, OT-I^{WT}, or OT-I^{SA} cells. Each dot represents one mouse;

bars show mean ± SEM. (**D**) Heatmap showing the relative expression of the indicated phenotypic and functional markers used to define activated, activated central-memory-like, effector-like, and exhausted-like $CD8^+$ T-cell states within the liver metastatic microenvironment. (**E**) Dose-titration analysis of $OT\text{-}I^{SA}$ ACT in mice bearing established $MC38^{OVA}$ liver metastases. Mice received NaCl or the indicated numbers of activated $OT\text{-}I^{SA}$ cells at day 14 after tumor-cell injection, and survival was monitored over time. The arrow indicates the time of ACT. (**F**) Schematic representation of the combination experiment. C57BL/6 mice were injected with $MC38^{OVA}$ cells via the mesenteric vein on day 0. Metastatic burden was assessed by MRI on day 14, after which mice received NaCl, a subcurative dose of $OT\text{-}I^{SA}$ cells, or $OT\text{-}I^{SA}$ cells combined with continuous IFNα administration. Endpoint MRI and tissue analyses were performed on day 28. (**G**) Incidence of metastatic outgrowth in mice treated with NaCl, $OT\text{-}I^{SA}$ cells, or $OT\text{-}I^{SA}$ cells plus IFNα. (**H**) Representative contrast-enhanced T1- and T2-weighted MRI acquired before treatment at day 14 and at endpoint on day 28. Red dashed lines delineate hepatic metastatic lesions. Scale bars, 5 mm. (**I**) MRI-based quantification of the total number of hepatic metastatic lesions at day 14, immediately before treatment, and at day 28 in mice treated with NaCl, a subcurative dose of $OT\text{-}I^{SA}$ cells, or $OT\text{-}I^{SA}$ cells combined with continuous IFNα administration. Each dot represents one mouse (NaCl, $n = 4$; $OT\text{-}I^{SA}$, $n = 5$; $OT\text{-}I^{SA}$ + IFNα, $n = 5$); bars show mean ± SEM. Groups were compared separately at each time point using ordinary one-way ANOVA followed by Holm-Šídák's multiple-comparisons test for all pairwise comparisons. P values are shown. (**J**) MRI-based quantification of total hepatic tumor volume at day 14, immediately before treatment, and at day 28 in mice treated with NaCl, a subcurative dose of $OT\text{-}I^{SA}$ cells, or $OT\text{-}I^{SA}$ cells combined with continuous IFNα administration. Each dot represents one mouse; bars show mean ± SEM. One-way ANOVA followed by Holm-Šídák's multiple-comparisons test for all pairwise comparisons. P values are shown. (**K**) Representative CD3 immunohistochemical staining of $MC38^{OVA}$ liver metastases from mice treated with NaCl, a subcurative dose of $OT\text{-}I^{SA}$ cells, or $OT\text{-}I^{SA}$ cells combined with continuous IFNα administration. (**L**) Violin plots showing the percentage of intralesional $CD3^+$ area in $MC38^{OVA}$ metastases from mice treated with NaCl, $OT\text{-}I^{SA}$ cells, or $OT\text{-}I^{SA}$ cells plus IFNα. Groups were compared using a Kruskal-Wallis test followed by Dunn's multiple-comparison test; adjusted P values are shown.

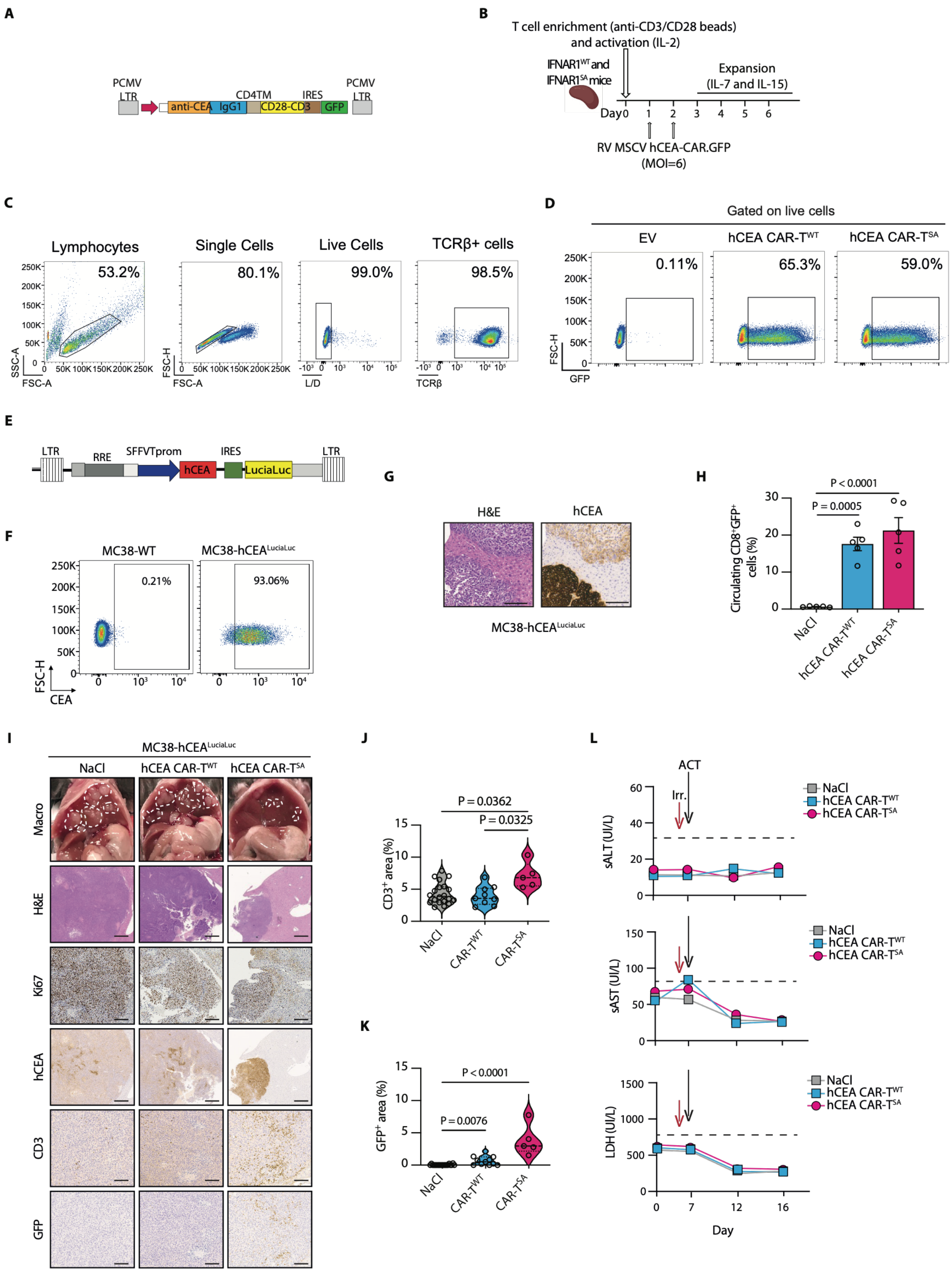

Fig. S8. Generation and characterization of IFNAR1^{WT} and IFNAR1^{SA} hCEA CAR-T cells and MC38-hCEA^{LuciaLuc} target cells.

(**A**) Schematic representation of the MSCV-based hCEA CAR retroviral construct encoding the anti-CEA scFv, IgG1 hinge, CD28 transmembrane domain, CD28-CD3ζ signaling domains, and

an IRES-GFP cassette (with GFP used as a surrogate marker of CAR expression). (**B**) Experimental workflow for CAR-T generation from splenic T cells isolated from IFNAR1^{WT} or IFNAR1^{SA} donor mice: enrichment/activation with anti-CD3/CD28 beads in the presence of IL-2, retroviral transduction with MSCV hCEA-CAR.GFP (MOI = 6 at days 1 and 2), and expansion with IL-7/IL-15. (**C**) Representative flow-cytometry gating strategy for CAR-T analyses: lymphocytes → singlets → live cells (L/D^{-}) → TCRβ^{+} cells. Percentages indicate the frequency of cells within the indicated gates. (**D**) Representative flow-cytometry plots showing GFP^{+} transduction efficiency in empty vector (EV), hCEA CAR-T^{WT}, and hCEA CAR-T^{SA} populations gated on live cells. Percentages indicate GFP^{+} cells within the indicated gates. (**E**) Schematic representation of the lentiviral vector used to generate MC38-hCEA^{LuciaLuc} cells encoding hCEA under the SFFV promoter and secreted Lucia luciferase reporter under an IRES. (**F**) Flow-cytometry validation of hCEA expression in MC38-hCEA^{LuciaLuc} compared with MC38-WT control. Percentages indicate hCEA^{+} cells within the indicated gates. (**G**) Representative H&E and hCEA immunohistochemistry (IHC) of MC38-hCEA^{LuciaLuc} liver metastatic lesions at endpoint (day 22). Scale bar, 200 µm. (**H**) Frequency of circulating CD8^{+}GFP^{+} CAR-T cells in peripheral blood on day 9 after intravenous (i.v.) adoptive transfer of hCEA CAR-T^{WT} or hCEA CAR-T^{SA} cells (1 x 10^{7}). Each dot represents one mouse; bars indicate mean ± SEM. One-way ANOVA, P values are shown. (**I**) Representative macroscopic liver images, H&E, and IHC staining for Ki67, hCEA, CD3, and GFP from MC38-hCEA^{LuciaLuc} tumor-bearing mice treated with NaCl, hCEA CAR-T^{WT}, or hCEA CAR-T^{SA}. Dashed lines delineate surface metastatic lesions in the macroscopic images. Scale bar for H&E, Ki67, and hCEA, 500 µm; for CD3 and GFP, 200 µm. (**J-K**) Violin plot showing CD3^{+} area (%) (J) and GFP^{+} area (%) (K) within liver lesions described in (I). Each dot represents one metastatic lesion. Statistical significance was assessed using Kruskal-Wallis tests followed by Dunn's multiple-comparisons test; P values are shown. (**L**) Longitudinal serum injury markers (sALT, sAST, LDH) in MC38-hCEA^{LuciaLuc} tumor-bearing mice treated with NaCl, hCEA CAR-T^{WT}, or hCEA CAR-T^{SA}; red arrows indicate sublethal irradiation (Irr.), and black arrows indicate adoptive cell transfer (CAR-T infusion). Dashed lines indicate the upper reference limit.

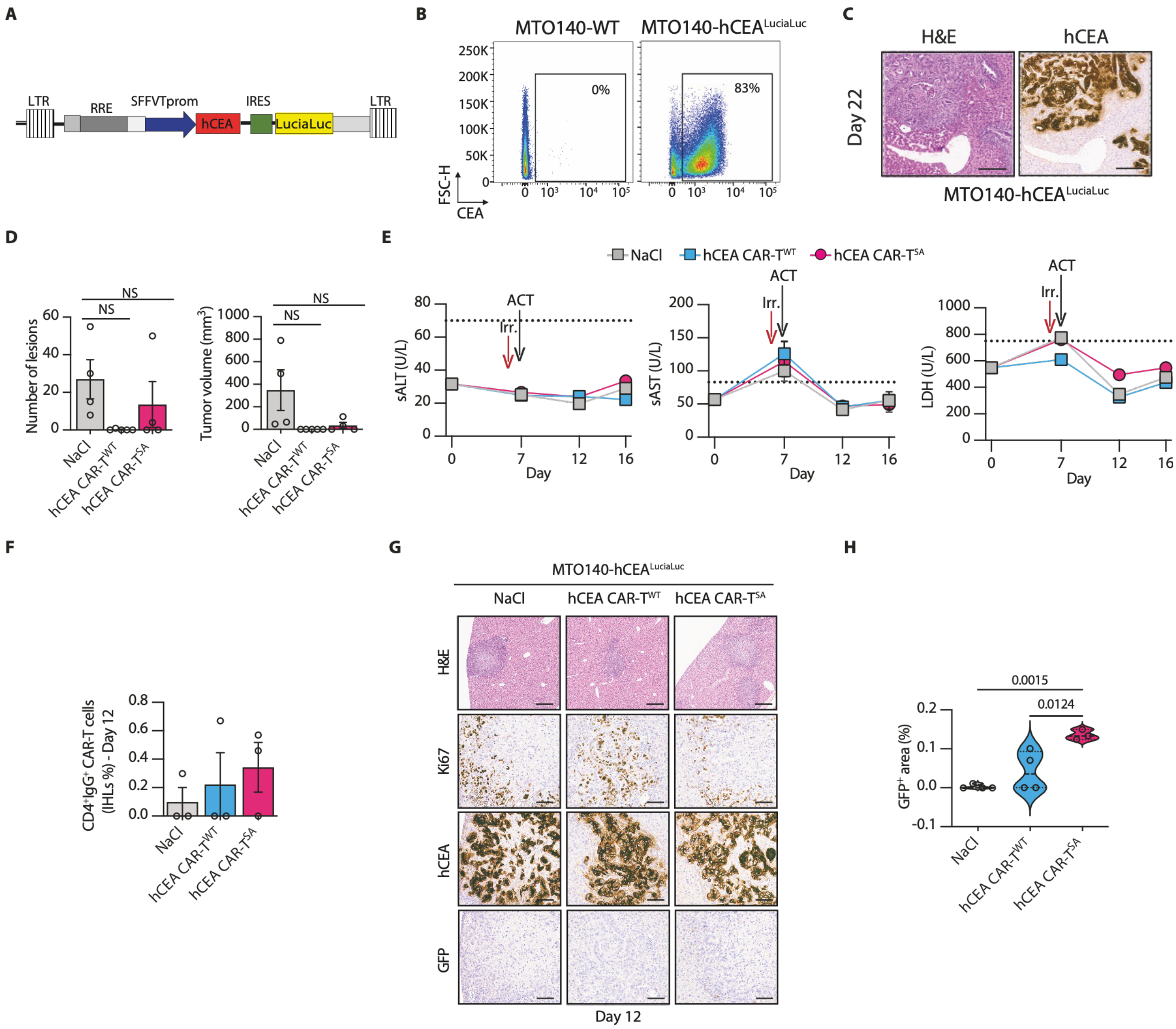

Fig. S9. Engineering and validation of the MTO140-hCEA^{LuciaLuc} model and longitudinal safety monitoring during hCEA CAR-T therapy.

(**A**) Schematic representation of the lentiviral vector used to generate MTO140-hCEA^{LuciaLuc} cells. (**B**) Flow-cytometry validation of hCEA expression in MTO140-hCEA^{LuciaLuc} compared with parental MTO140-WT cells. The indicated percentages represent hCEA-positive cells within the indicated gates**.** (**C**) Representative H&E and hCEA IHC confirming hCEA expression in MTO140-hCEA^{LuciaLuc} liver metastatic lesions at endpoint (day 22). Scale bar, 200 um. (**D**) Quantification of metastatic burden at day 22 by MRI as number of lesions (left) and total tumor volume (mm^3, right) in mice treated as indicated. Each dot represents a mouse; bars indicate mean ± SEM. One-way ANOVA; P values are shown. NS, not significant. (**E**) Serum liver injury markers (sALT, sAST and LDH) measured longitudinally in mice treated with NaCl, hCEA CAR-T^{WT}, or hCEA CAR-T^{SA}; red arrows indicate sublethal irradiation (Irr.), black arrows indicate adoptive cell transfer (ACT), and dashed lines indicate the upper reference limits. (**F**) Frequency of CD4$^+$IgG$^+$CAR-T among intrahepatic leukocytes (IHLs) from mice bearing MTO140-hCEA^{LuciaLuc} liver metastases at day 12 post-CAR-T infusion. Each dot represents one mouse; bars

show mean ± SEM. (**G**) Representative H&E and IHC staining for Ki67, hCEA, and GFP from MTO140-hCEA^{LuciaLuc} tumor-bearing mice treated with NaCl, hCEA CAR-T^{WT}, or hCEA CAR-T^{SA} at day 12. Scale bar for H&E, 500 µm; for Ki67, hCEA, and GFP, 200 µm. (**H**) Violin plot showing GFP^{+} area (%) within liver lesions described in (G). Each dot represents one metastatic lesion. One-way ANOVA, P values are shown.

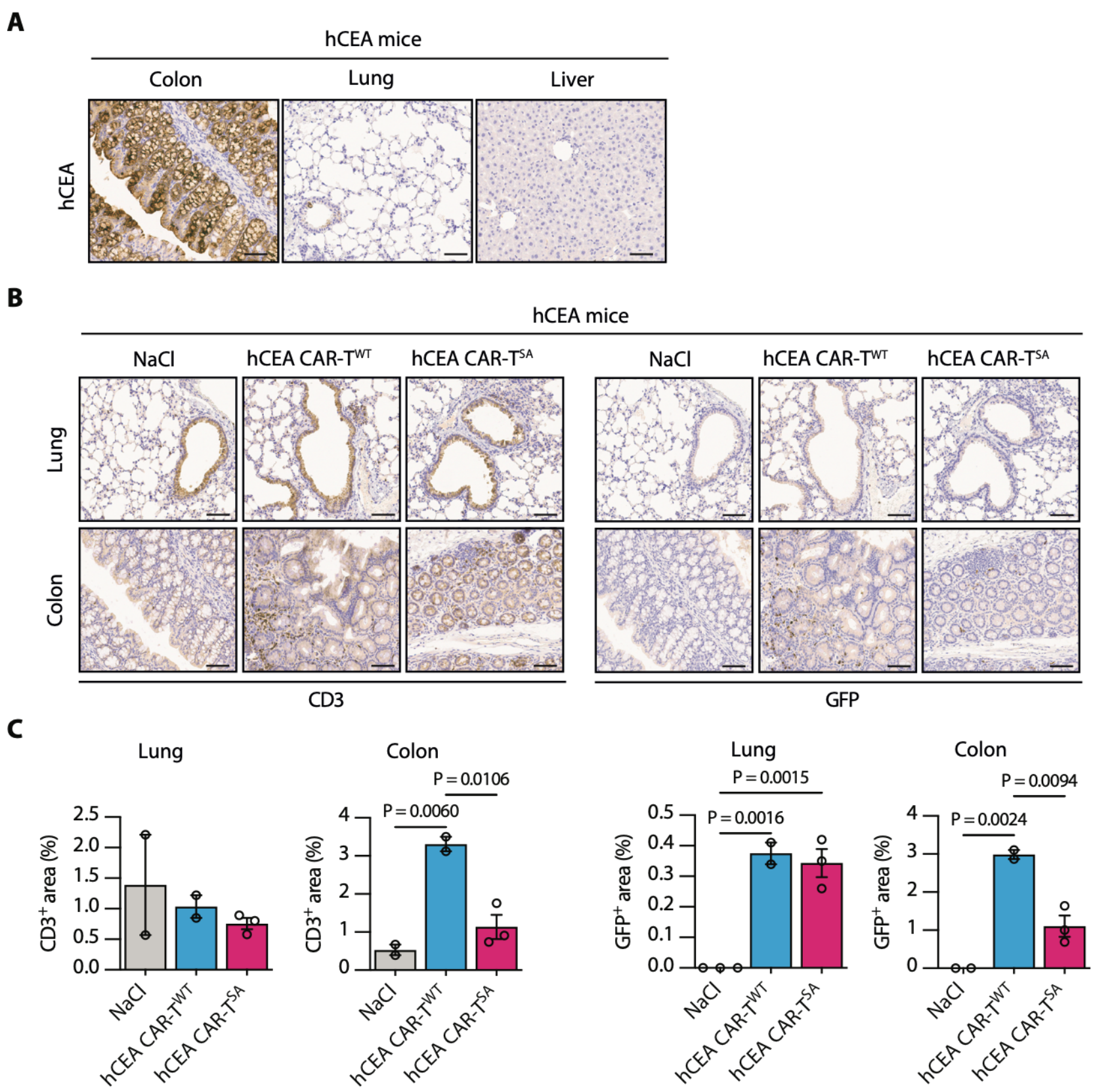

Fig. S10. On-target/off-tumor reactivity of hCEA CAR-T cells in non-tumoral tissues and organs physiologically expressing hCEA.

(**A**) Representative immunohistochemistry (IHC) for hCEA in colon, lung, and liver sections from healthy hCEA-transgenic mice. Scale bar, 200 µm.

(**B**) Representative IHC for CD3 (left) and GFP (right) in lung and colon sections from hCEA-transgenic mice treated with NaCl, hCEA CAR-T^{WT}, or hCEA CAR-T^{SA}. Scale bar, 200 µm.

(**C)** Quantification of $CD3^+$ area (%) and GFP^+ area (%) in lung and colon sections shown in (B). Each dot represents one mouse. Data are shown as mean ± SEM. One-way ANOVA followed by Tukey's multiple-comparisons test; P values are shown.

Table S1. Demographic, histological, and clinical oncological characteristics of the patients enrolled in "IFNAR1" study.

**Clinicopathological features**	**Number of patients (N=44)**
Age [median; (range)]	72 (33-90)
Sex (N, %)	
Male	27 (61.4)
Female	17 (38.6)
Primary tumor resection (N, %)	
Yes	44 (100)
No	0 (0)
Primary tumor sidedness (N, %)	
Rectum and left colon *	30 (68.2)
Right colon ^	13 (29.5)
Total colon	1 (2.3)
Grade (N, %)	
G1	1 (2.3)
G2	26 (59)
G3	7 (16)
Unknown	10 (22.7)
KRAS mutant (N, %)	
Mutant	15 (34.1)
Wild-type	9 (20.4)
Unknown	20 (45.5)
Mismatch repair (MMR) status (N, %)	
MSI	0 (0)
MSS	13 (29.5)
Unknown	31 (70.5)
Neoadjuvant chemotherapy (N, %)	
Yes	25 (56.8)
No	19 (43.2)
TRG grade (N, %)	
1	2 (4.5)
2	5 (11.4)
3	6 (13.6)
4	0 (0)
Not Applicable	31 (70.5)

Keys: ^ Located in caecum, ascending colon, liver flexure and transverse colon. * Located in splenic flexure, descending colon and sigmoid colon. MSS, microsatellite stable; MSI, microsatellite instable; TRG, tumor regression grade.

Table S2. Demographic, histological, and clinical oncological characteristics of the patients enrolled in "ACC_ORG-LiMeT" study.

**Clinicopathological features**	**Number of patients (N=15)**
Age [median; (range)]	66.2 (51-81)
Sex (N, %)	
Male	10 (66.7)
Female	5 (33.3)
Primary tumor resection (N, %)	
Yes	15 (100)
No	0 (0)
Primary tumor sidedness (N, %)	
Rectum and left colon *	13 (86.7)
Right colon ^	2 (13.3)
Total colon	0 (0)
Grade (N, %)	
G1	0 (0)
G2	8 (53.3)
G3	6 (40)
Unknown	1 (6.7)
KRAS mutant (N, %)	
Mutant	5 (33.3)
Wild-type	6 (40)
Unknown	4 (26.7)
Mismatch repair (MMR) status (N, %)	
MSI	1 (6.7)
MSS	3 (20.0)
Unknown	11 (73.3)
Neoadjuvant chemotherapy (N, %)	
Yes	6 (40.0)
No	9 (60.0)
TRG grade (N, %)	
1	0 (0)
2	2 (13.3)
3	1 (6.7)
4	5 (33.3)
Not Applicable	7 (46.7)

Keys: ^ Located in caecum, ascending colon, liver flexure and transverse colon. * Located in splenic flexure, descending colon and sigmoid colon. MSS, microsatellite stable; MSI, microsatellite instable; TRG, tumor regression grade.

Table S3 List of Antibodies for Immunohistochemistry & Immunofluorescence

Primary Antibody for Immunohistochemistry	Vendor	CAT#	RRID#
Anti-human-IFNAR1 (dilution 1:50)	Sigma-Aldrich	HPA018015	AB_1851433
Anti-mouse-F4/80 (dilution 1:200)	Bio-Rad	MCA497	AB_2098196
Anti-human-CD66/CEA/CEACAM5 (dilution 1:200)	NSJ Bioreagents	V3599	
Anti-mouse-phospho-STAT1(Tyr701) (dilution 1:100)	Cell Signaling	9167	AB_561284
Anti-mouse-CD3 (dilution 1:100)	Abcam	ab21703	AB_446487
Anti-mouse-Ki-67 (dilution 1:200)	Thermo Fisher Scientific	MA5-14520	AB_10979488
Anti-mouse Cleaved Caspase-3 (Asp175) (dilution 1:100)	Cell Signaling	9661	AB_2341188
Anti-GFP (dilution 1:100)	Thermo Fisher Scientific	A-11122	AB_221569
**Primary Antibody for Immunofluorescence**	**Vendor**	**CAT#**	**RRID#**
Anti-human-IFNAR1 (dilution 1:50)	Sigma-Aldrich	HPA029226	
Anti-human-CD4 (dilution 1:50)	Abcam	AB183685	AB_2686917
Anti-human-CD8 (dilution 1:50)	Thermo Fisher Scientific	14-0195-82	AB_2637159
Anti-human-MUM1/IRF4 (clone MUM1p)	Thermo Fisher Scientific	MA1-25525	AB_794981
Anti-human/mouse-PAN-IFNα (dilution 1:50)	Thermo Fisher Scientific	PA5-119649	AB_2913222
Anti-human-IFNε (dilution 1:50)	R&D	MAB9147	AB_3659338
Anti-mouse-IFNAR1 (dilution 1:50)	Sino Biological	50469-R110	AB_2860509
Anti-mouse-CD31/PECAM1 (dilution 1:300)	R&D Systems	AF3628	AB_2161028
Anti-mouse-CD8 (dilution 1:100)	Biolegend	100702	AB_312741
Anti-mouse-p-STAT1(Tyr701) (dilution 1:100)	Cell Signaling	9167	AB_561284
Anti-mouse Cleaved Caspase-3 (Asp175) (dilution 1:100)	Cell Signaling	9661	AB_2341188
Anti-GFP (dilution 1:100)	Rockland	600901215	AB_1537402
**Secondary Antibody for Immunofluorescence (dilution)**	**Vendor**	**CAT#**	**RRID#**
Donkey anti-Rabbit IgG (H+L), Alexa Fluor 488 (dilution 1:200)	Thermo Fisher Scientific	A32790	AB_2762833
Donkey anti-Rabbit IgG, Alexa Fluor 647 (dilution 1:200)	Thermo Fisher Scientific	A-31573	AB_2536183
Donkey anti-Goat IgG (H+L), Alexa Fluor 546 (dilution 1:200)	Thermo Fisher Scientific	A-11056	AB_2534103
Donkey anti-Rat IgG (H+L), Alexa Fluor 647 (dilution 1:200)	Thermo Fisher Scientific	A48272	AB_2893138

Donkey anti-mouse IgG (H+L) AF594 (dilution 1:200)	Thermo Fisher Scientific	A-21203	AB_2535789
Goat anti-chicken IgY (H+L) Alexa Fluor 488 (dilution 1:200)	Thermo Fisher Scientific	A-11039	AB_2534096

Table S4. List of Reagents & Commercial assays

Reagent Name	Vendor	CAT#
Recombinant mouse IFNα, carrier-free	Biolegend	752808
Alzet osmotic pumps 1004	Alzet	10104846
VeriKine-HS Mouse IFNα All-Subtype ELISA Kit	PBL Assay Sci	42115-1
B16-Blue™ IFN-α/β Cells	InvivoGen	bb-ifnt1
QUANTI-Blue™ Solution	InvivoGen	rep-qbs
QUANTI-Luc™: Luciferase Detection Reagent	InvivoGen	rep-qlc4lg1
Percoll®	Sigma-Aldrich	P4937-500ML
ACK Lysing Buffer	Thermo Fisher	A1049201
Zinc formalin fixative	Sigma-Aldrich	Z2902-3.75L
RPMI-1640 Medium	Thermo Fisher	61870044
Matrigel Matrix	BD Bioscience	354248
NH2-SIINFEKL-COOH (SL-8 peptide)	Proimmune	GMPT5995
Lipofectamine 2000	Thermo Fisher Scientific	11668019
Polybrene (Hexadimethrine bromide)	Sigma-Aldrich	TR-1003-G
Pan T Cell Isolation Kit II, mouse	Miltenyi Biotec	130-095-130
LS Columns	Miltenyi Biotec	130-042-401
Dynabeads™ Mouse T-Activator CD3/CD28	Thermo Fisher	11452D
Recombinant human IL-2	R&D Systems	BT-002-010
Recombinant murine IL-7	R&D Systems	207-IL-005
Recombinant murine IL-15	R&D Systems	247-ILB-005
7-AAD Viability staining solution	Biolegend	420404
Live/Dead fixable near-IR dead cell stain kit, dilution 1:500	Thermo Fisher Scientific	L34975
Live/Dead fixable Green dead cell stain kit, dilution 1:100	Thermo Fisher	L34970
Brefeldin A	Sigma-Aldrich	B7651
Foxp3/Transcription Factor Staining buffer set	Thermo Fisher	00-5523-00
UltraComp eBeads™ Compensation Beads	Thermo Fisher	01-2222-42
ReliaPrep™ RNA Cell Miniprep System	Promega	Z6011
ReliaPrep™ RNA Tissue Miniprep System	Promega	Z6111
RNeasy Mini Kit	Qiagen	74104
QIAamp DNA Mini kit	Qiagen	51304
TURBO DNA-free™ kit	Ambion	AM1907
SuperScript™ IV VILO™ Master Mix	Thermo Fisher	11756050
TaqMan™ Fast Advanced Master Mix	Thermo Fisher	4444963
SYBR™ Green PCR Master Mix	Thermo Fisher	4364344
SD-208 inhibitor	Aurogene	S7624
LY2228820 inhibitor	Aurogene	S1494
Opal 6-Plex Detection kit (Opal 480, Opal 520, Opal 620, Opal 690)	Quanterix	NEL811001KT
ProCyte Kit	Idexx	9926306-00

Table S5. List of Oligonucleotides for molecular biology.

Oligonucleotides	Vendor	CAT#
Taqman probe IFNA1	Thermo Fisher Sci	Hs00256882_s1
Taqman probe IFNA2	Thermo Fisher Sci	Hs00265051_s1
Taqman probe IFNA4	Thermo Fisher Sci	Hs01681284_sH
Taqman probe IFNA5	Thermo Fisher Sci	Hs04186137_sH
Taqman probe IFNA6	Thermo Fisher Sci	Hs00819627_s1
Taqman probe IFNA7	Thermo Fisher Sci	Hs01652729_s1
Taqman probe IFNA8	Thermo Fisher Sci	Hs00266883_s1
Taqman probe IFNA10	Thermo Fisher Sci	Hs03406429_gH
Taqman probe IFNA13	Thermo Fisher Sci	Hs04190680_gH
Taqman probe IFNA14	Thermo Fisher Sci	Hs00353663_s1
Taqman probe IFNA16	Thermo Fisher Sci	Hs03005057_sH
Taqman probe IFNA17	Thermo Fisher Sci	Hs00819693_sH
Taqman probe IFNA21	Thermo Fisher Sci	Hs00353738_s1
Taqman probe IFNB1	Thermo Fisher Sci	Hs01077958_s1
Taqman probe IFNE	Thermo Fisher Sci	Hs00703565_s1
Taqman probe IFNK	Thermo Fisher Sci	Hs00737883_m1
Taqman probe IFNW1	Thermo Fisher Sci	Hs00958789_s1
Taqman probe IFNAR1	Thermo Fisher Sci	Hs01066116_m1
Taqman probe *Ifna1/Ifna6/Ifna5*	Thermo Fisher Sci	Mm03030145_gH
Taqman probe *Ifna2*	Thermo Fisher Sci	Mm00833961_s1
Taqman probe *Ifna4*	Thermo Fisher Sci	Mm00833969_s1
Taqman probe *Ifna5*	Thermo Fisher Sci	Mm00833976_s1
Taqman probe *Ifna6*	Thermo Fisher Sci	Mm01703458_s1
Taqman probe *Ifna7*	Thermo Fisher Sci	Mm02525960_s1
Taqman probe *Ifna9*	Thermo Fisher Sci	Mm00833983_s1
Taqman probe *Ifna11/Ifna2*	Thermo Fisher Sci	Mm04207507_gH
Taqman probe *Ifna12*	Thermo Fisher Sci	Mm00616656_s1
Taqman probe *Ifna13*	Thermo Fisher Sci	Mm01731013_s1
Taqman probe *Ifna14*	Thermo Fisher Sci	Mm01703465_s1
Taqman probe *Ifna15*	Thermo Fisher Sci	Mm01267666_sH
Taqman probe *Ifna16*	Thermo Fisher Sci	Mm01703434_s1
Taqman probe *Ifnab*	Thermo Fisher Sci	Mm00833443_s1
Taqman probe *Ifnb1*	Thermo Fisher Sci	Mm00439552_s1
Taqman probe *Ifne*	Thermo Fisher Sci	Mm00616542_s1
Taqman probe *Ifnk*	Thermo Fisher Sci	Mm02529417_s1
Taqman probe *Ifnz*	Thermo Fisher Sci	Mm02525738_g1
Taqman probe *Irgm1*	Thermo Fisher Sci	Mm00492596_m1
Taqman probe *IFNAR1*	Thermo Fisher Sci	Mm00439544_m1
Forward IFNa4-407L (NM_010504)	Eurofins	AGCCTGTGTGATGCAGGAA
Reverse IFNa4(502R) (NM_010504)	Eurofins	GGCACAGAGGCTGTGTTTCT
Forward IFNa2(457L) (NM_010503)	Eurofins	ACCTGAGAGAGAAGAAACACAGC
Reverse IFNa2(527R) (NM_010503)	Eurofins	GCAGCAAGTTGACTGAGGAA

Forward IFNb1(8L) (NM_010510)	Eurofins	CTGGCTTCCATCATGAACAA
Reverse IFNb1(62R) (NM_010510)	Eurofins	AGAGGGCTGTGGTGGAGAA
Forward mB actin FW	Eurofins	AAGAGAAGGGTTACCCGGGATA
Reverse mB actin RW	Eurofins	CCTAAGGCCAACCGTGAAAA
Forward B18R fw	Eurofins	CAGCATGTATGTTCGGAGGC
Reverse B18R rv	Eurofins	TTAGAAACCTGTCGCCGTCT
Forward GAPDH 348F	Eurofins	TTCACCACCATGGAGAAGGC
Reverse GAPDH 584R	Eurofins	GGCATGGACTGTGGTCATGA
Forward ISG15 (23L) NM_015783.3	Eurofins	GGGGGAGAGTCGATCCAG
ReverseISG15(143R) NM_015783.3	Eurofins	CCCCAGCATCTTCACCTTTA
Forward IFIT3-(16L) NM_010501.2	Eurofins	TGAACTGCTCAGCCCACA
Reverse IFIT3(91R) NM_010501.2	Eurofins	TCCCGGTTGACCTCACTC
Forward IFIT1 (886L) NM_008331.3	Eurofins	TCTAAACAGGGCCTTGCAG
Reverse IFIT1(949R) NM_008331.3	Eurofins	GCAGAGCCCTTTTTGATAATGT

Table S6. List of Antibodies for Flow Cytometry.

Primary Antibody	Vendor	CAT#	RRID#
Anti-human-CD66/CEA/CEACAM5 APC (clone REA876)	Miltenyi Biotec	130114218	AB_2726490
Anti-mouse CD16/CD32 (Mouse BD Fc Block™) Clone 2.4G2	BD Biosciences	553141	AB_394656
Anti-mouse-CD11b BV510 (clone M1/70)	Biolegend	101263	AB_2629529
Anti-mouse-CD11b BV510 (clone M1/70)	Biolegend	101224	AB_755986
Anti-mouse-CD11c AF647 (clone N418)	Biolegend	117312	AB_389328
Anti-mouse-CD11c PE-Cy7 (clone N418)	Thermo Fisher Scientific	25011482	AB_469590
Anti-mouse-CD19 BUV737 (clone 1D3)	BD Biosciences	612781	AB_2870110
Anti-mouse-CD19 PB (clone 6D5)	Biolegend	115523	AB_439718
Anti-mouse-CD206 BV711 (clone C068C2)	Biolegend	141727	AB_2565822
Anti-mouse-CD25 APC-Cy7 (clone PC61)	Biolegend	102026	AB_830745
Anti-mouse-CD25/IL2Ra BV510 (clone PC61)	BD Biosciences	563037	AB_2737969
Anti-mouse-CD279/PD-1 BUV737(clone 29F.1A12)	Biolegend	135231	AB_2566158
Anti-mouse-CD279/PD1 PECy5 (clone EH12.2H7)	Biolegend	329971	AB_2910394
Anti-mouse-CD3 APC-Cy7 (clone 145-2C11)	Thermo Fisher Scientific	17003182	AB_469315
Anti-mouse-CD31 BV421 (clone MEC13.3)	BD Biosciences	562939	AB_2665476
Anti-mouse-CD326 (EpCAM) APC-Cy7 (clone G8.8)	Biolegend	118218	AB_2098648
Anti-mouse-CD366/Tim-3 BUV615 (clone RMT3-23)	BD Biosciences	753150	AB_3686849
Anti-mouse-CD4 AF488 (clone GK 1.5)	Thermo Fisher Scientific	53004182	AB_469893
Anti-mouse-CD4 BV750 (clone RM 4-5)	BD Biosciences	747344	AB_2872045
Anti-mouse-CD4 eFluor450 (clone RM 4-5)	Thermo Fisher Scientific	48-0042-82	AB_1272194
Anti-mouse-CD44 BUV563 (clone IM7)	BD Biosciences	741227	AB_2870781
Anti-mouse-CD44 BV510 (clone IM7)	Biolegend	103044	AB_2650923
Anti-mouse-CD45 APC (clone 30-F11)	Biolegend	103112	AB_312977
Anti-mouse-CD45 APC-Cy7 (clone 30-F11)	Biolegend	103116	AB_312981
Anti-mouse-CD45 BUV395 (clone 30-F11)	BD Biosciences	564279	AB_2651134
Anti-mouse-CD45.1 PerCP/Cy5.5 (clone A20)	Biolegend	110728	AB_893346
Anti-mouse-CD45.2 PB (clone 104)	Biolegend	109820	AB_492872
Anti-mouse-CD45R/B220 AF647 (clone RA3-6B2)	Biolegend	103229	AB_492875
Anti-mouse-CD62L AF488 (clone MEL-14)	Biolegend	104420	AB_493376
Anti-mouse-CD62L BV650 (clone MEL14)	Biolegend	104453	AB_2800559
Anti-mouse-CD69 APC (clone H1.2F3)	Biolegend	104514	AB_492843
Anti-mouse-CD8a AF488 (clone 53-6.7)	Biolegend	100723	AB_389304
Anti-mouse-CD8a APC (clone 53-6.7)	Biolegend	100712	AB_312751
Anti-mouse-CD8a APC-Cy7 (clone 53-6.7)	Biolegend	100714	AB_312753
Anti-mouse-CD8a BV711 (clone 53-6.7)	BD Biosciences	563046	AB_2737972
Anti-mouse-CD8a PE-Cy7 (clone 53-6.7)	Biolegend	100722	AB_312761

Anti-mouse-CD8a PerCP/Cy5.5 (clone 53-6.7)	Biolegend	100733	AB_2075239
Anti-mouse-F4/80 AF647 (clone BM8)	Biolegend	123122	AB_893480
Anti-mouse-F4/80 PECy7 (clone BM8)	Biolegend	123114	AB_893478
Anti-mouse-Granzyme B AF647 (clone GB11)	Biolegend	515406	AB_2566333
Anti-mouse-I-A/I-E PB (clone M5/114.15.2)	Biolegend	107620	AB_493527
Anti-mouse-IFNAR1 APC (clone MAR1-5A3)	Biolegend	127313	AB_2122746
Anti-mouse-IFNAR1 PE (clone MAR1-5A3)	Biolegend	127312	AB_2248800
Anti-mouse-IFNγ PE-CF594 (clone XMG1.2)	BD Biosciences	562333	AB_11154588
Anti-mouse-IFNγ Pacific Blue (clone XMG1.2)	Biolegend	505818	AB_893526
Anti-mouse-IgG1 PE (clone A85-1)	BD Biosciences	550083	AB_393553
Anti-mouse-Ki-67 PeCy7 (clone B56)	BD Biosciences	561283	na
Anti-mouse-Ly6C FITC (clone AL-21)	BD Biosciences	561085	AB_10584332
Anti-mouse-Ly6G APC-Cy7 (clone 1A8)	Biolegend	127623	AB_10645331
Anti-mouse I-A/I-E Alexa Fluor 700 (clone M5/114.15.2)	Biolegend	107622	AB_493727
Anti-mouse-NGFR AF647 (clone C40-1457)	BD Biosciences	560326	AB_1645403
Anti-mouse-Nk1.1 BV421 (clone PK136)	Biolegend	108741	AB_2562561
Anti-mouse-PD-L1 PECy7 (clone MIH5)	Thermo Fisher Scientific	25598282	AB_2573509
Anti-mouse-pSTAT(Y701) AF488 (clone 4a)	BD Biosciences	612596	AB_399879
Anti-mouse-TCRbeta PECy7 (clone KT11)	Biolegend	125916	AB_2800611
Anti-mouse-TIM-3 PECy7 (clone RMT3-23)	Biolegend	119716	AB_2571933
Anti-mouse-TNF-α BV510 (clone MP6-XT22)	Biolegend	506339	AB_2563127

Data S1. Source data for Fig. 1 and Fig. S1.
Data S2. Source data for Fig. 2 and Fig. S2.
Data S3. Source data for Fig. 3 and Fig. S3.
Data S4. Source data for Fig. 4 and Fig. S4.
Data S5. Source data for Fig. 5.
Data S6. Source data for Fig. 6 and Fig. S6.
Data S7. Source data for Fig. 7 and Fig. S7.
Data S8. Source data for Fig. 8 and Figs. S8 to S10.
